# Risk of cancer in periodontal disease: an umbrella review of meta-analyses

**DOI:** 10.3389/fimmu.2026.1839715

**Published:** 2026-07-10

**Authors:** Jing Yuan, Shixuan Wang, Siqi Xia, Simin Lou, Hujia Yang, Jiaojiao Wei, Yun Chen, Luobin Yu, Yuxuan Du, Lulin Yu, Leitao Sun, Shuhua Wang

**Affiliations:** 1 School/hospital of Stomatology, Zhejiang Chinese Medical University, Hangzhou, China; 2 The First Affiliated Hospital of Zhejiang Chinese Medical University (Zhejiang Provincial Hospital of Chinese Medicine), Hangzhou, Zhejiang, China; 3 Academy of Chinese Medical Science, Zhejiang Chinese Medical University, Hangzhou, China

**Keywords:** cancer, cancer risk, periodontal disease, periodontitis, umbrella review

## Abstract

**Objective:**

This study aims to summarize and appraise the credibility of existing epidemiological evidence on the association between periodontal diseases (PDs) and the risk of site-specific cancers.

**Methods:**

PubMed, Embase, and Cochrane Library were searched from inception to June 2026. Meta-analyses of observational studies investigating PDs and cancer risk were included. Each association was classified into five levels according to a predefined criterion, which included statistical significance, heterogeneity, sample size, and bias assessment. AMSTAR 2 was used to evaluate the quality of studies. Corrected covered area and the author’s own meta-analysis were performed to ensure a rigorous methodology.

**Results:**

In total, 66 associations from 38 meta-analyses investigating 14 cancer types were appraised, including head and neck cancer (HNC), oral, lung, breast, esophageal, gastric, pancreatic, liver, colorectal, bladder, kidney, prostate, melanoma, and hematopoietic and lymphatic cancers. Among 23 main associations, highly suggestive evidence was identified in associations between PDs (odds ratio [OR], 2.42; 95% confidence interval [CI], 1.85–3.17) in HNC, and PDs in oral cancer (OR, 2.94; 95% CI, 2.13–4.07). Another seven associations were recommended as suggestive evidence (class III). Fourteen associations were classified as weak (class IV) or showing no significant evidence (class V).

**Conclusion:**

Associations between PDs and site-specific cancers demonstrated varying levels of evidence. This analysis highlighted strong correlations in HNC and oral cancer, while failing to show credible evidence for the remaining 12 cancers. Overall, our study yielded novel insights into oral health interventions, calling for the incorporation of periodontal health into public health policy.

**Systematic Review Registration:**

https://www.crd.york.ac.uk/PROSPERO/view/CRD42024585375, identifier CRD42024585375.

## Introduction

Periodontal disease (PD) is a chronic infectious disorder that affects the supporting tissues of the teeth, gingiva, periodontal ligament, cementum, and alveolar bone. Severe periodontitis affects approximately 10% of the global population, making it an important contributor to the global burden of chronic diseases (1, 2). Bacteria-related inflammation and progressive periodontal tissue destruction, coupled with the cross-talk between environmental and hereditary factors, characterize the entire disease course of PD, from gingivitis, a mild inflammatory condition confined to the gingiva, to periodontitis, which involves the destruction of deeper periodontal tissues, and ultimately to tooth loss, the most severe consequence of advanced PD (3).

Accumulating epidemiological evidence has confirmed associations between PDs and systemic diseases, including cardiovascular diseases (4), and metabolic syndromes (5, 6). Beyond that, emerging evidence has suggested a potential association between PDs and various cancers. As reported, individuals with severe periodontitis were reported to have a 24% higher risk of cancer compared with their healthy counterparts (7).

Previous studies have suggested that PD-related inflammation could increase the levels of C-reactive protein, interleukin (IL)-6, and IL-1β (8, 9). Together with the periodontal pathogens, inflammatory mediators and microbial products may enter the bloodstream, potentially creating a systemic inflammatory environment that may favor carcinogenesis (10–12).

Epidemiologic studies have reported associations between PDs and cancer, whereas credible associations remain unclear. This is due to inconsistent findings and methodological heterogeneity. To address these gaps, an umbrella review was conducted to systematically synthesize and critically appraise existing evidence linking PDs to site-specific cancer risk. This study aims to identify robust and credible evidence, as well as to provide an evidence-based framework for future mechanistic research.

## Methods

The study protocol was registered in PROSPERO (CRD42024585375). We followed a standardized methodology (13), and the results were reported in accordance with the Preferred Reporting Items for Systematic Reviews and Meta-Analyses (PRISMA) (14) and Meta-analysis of Observational Studies in Epidemiology (MOOSE) (15) guidelines.

### Literature search

A systematic search was conducted in PubMed, Embase, and the Cochrane Library from inception to June 2026. To ensure comprehensive identification of relevant studies, we used key search terms and variations of text words related to PDs (including gingivitis, periodontitis, periodontal indices, disease classifications, and related synonyms), cancer, and meta-analysis. The complete search strategy is provided in the **Supplementary Table 2**. We also manually screened the reference lists of eligible reviews to identify additional relevant studies. Two authors independently conducted the literature search. Any disagreements were resolved through discussion with a third investigator.

### Inclusion and exclusion criteria

Articles were included if they met the following inclusion criteria: (1) systematic reviews and meta-analyses of observational epidemiological studies investigating the association between PDs and the risk of site-specific cancers; (2) providing effect estimates of the included primary studies, including hazard ratio (HR), odds ratio (OR) and risk ratio (RR), together with corresponding 95% confidence intervals (CIs) to enable re-analysis; and (3) reporting the number of cases and sample size for each primary study included in the meta-analysis.

The excluded criteria were as follows: (1) articles reporting only cancer prognosis; (2) articles investigating the association between oral interventions and cancer risk; (3) articles focusing on benign tumor or precancerous lesions (e.g., polyp); (4) studies focusing on overall cancer risk rather than site-specific cancers; (5) articles failed to provide forest plots; (6) articles without accessible full-text; and (7) narrative reviews, rapid reviews, experimental studies, commentaries, case reports, protocols, letters and posters.

Eligibility was assessed independently by two reviewers, and any disagreements were resolved through discussion or consultation with a third reviewer.

### Definition of periodontal diseases

In this umbrella review, periodontal exposures were classified according to the exposure definitions adopted in the original meta-analyses. No attempt was made to harmonize or redefine the exposure categories. Eligible exposures included clinically diagnosed periodontal conditions (primarily gingivitis and periodontitis) and clinical or radiographic indicators of periodontal inflammation or tissue destruction, such as alveolar bone loss (ABL). Tooth loss was not considered a surrogate marker of PDs because it may result from multiple causes unrelated to periodontal disease, potentially introducing exposure misclassification. No restrictions were imposed on the method of PD assessment, which could include self-reported measures, clinical examinations, dental records, or radiographic evaluations.

### Data extraction

Data were independently extracted by two reviewers, and disagreements were resolved through discussion and consensus. From each meta-analysis, we extracted the first author’s name, publication year, specific exposure (e.g., PDs, periodontitis, and other periodontal indicators), cancer type, number of primary studies included in each meta-analysis, measurement and definition of PDs, study design of the primary studies, effect estimates (HRs, RRs, and ORs) with 95% CIs, and the number of cases and total sample size.

When specific information was missing, conflicting, or unclear in the meta-analyses, we retrieved the relevant information from the original studies or directly contacted the corresponding authors for clarification. When discrepancies were identified between the primary studies and the meta-analyses, priority was given to data reported in the original studies.

### Management of overlapping

When multiple meta-analyses addressed the same research question (i.e., the same periodontal exposure and cancer outcome), all eligible articles were initially retained to ensure comprehensive evidence identification. Since direct inclusion of all overlapping meta-analyses may result in repeated incorporation of the same primary studies and consequently bias the synthesized evidence, overlap was subsequently quantified using the corrected covered area (CCA) (16). CCA is a validated method for assessing the degree of overlap among systematic reviews and meta-analyses and was calculated based on a citation matrix of included primary studies. According to established criteria, overlap was classified as slight (0%–5%), moderate (6%–10%), high (11%–15%), or critically high (> 15%). For associations with critically high overlap, it was considered substantial, indicating that the available meta-analyses were largely based on the same body of primary evidence. In these cases, the most comprehensive and methodologically robust meta-analysis was selected as the primary evidence source. For associations showing slight, moderate, or high overlap (CCA < 15%), additional evaluation was undertaken when meaningful discrepancies in the included primary studies were identified across reviews. In such circumstances, an author’s own meta-analysis (AOM) was performed to maximize evidence completeness while minimizing duplication of data (17). During the AOM process, all potentially eligible primary studies were reassessed independently by three investigators in accordance with the MOOSE guidelines. Studies derived from overlapping populations or the same research centers were carefully evaluated, and duplicate populations were excluded to avoid double-counting. The detailed selection process is shown in **Supplementary Figure 1**.

### Data analysis

#### Stratified and subgroup analyses

We additionally performed stratified analyses combining estimates by study design (cohort or case-control studies), region (Asia, Europe, America, or Africa), adjustment variables (whether adjusted for smoking and alcohol), and evaluation of PDs (self-report or clinical examination). Furthermore, subgroup analysis was conducted based on follow-up duration, when it was available.

#### Estimation of summary effect

For each association, we recalculated and adjusted the summary effect and corresponding 95% CI, along with the associated *p*-value by using a random effects model (REM) (18). When available, the most fully adjusted effect estimates were extracted.

#### Assessment of heterogeneity

*I*^2^ values were used to evaluate the proportion of heterogeneity (19, 20). Substantial inconsistency indicated the presence of genuine heterogeneity or bias. We then calculated the corresponding 95% CI to evaluate the uncertainty of heterogeneity estimates. *I*^2^ values of 50% were considered to indicate moderate heterogeneity, while values of 75% or more were considered to indicate high heterogeneity.

#### Estimation of prediction intervals

When more than three original articles were included, we calculated 95% prediction intervals (PIs), which could account for heterogeneity and reflect the range in which the effect estimates of future studies may fall (21). If the 95% PIs excluded the null, it suggested a statistically significant range of effect estimates.

#### Largest study significance

We assessed whether effect estimates from the largest research article included in the pooled analyses exhibited a significant *p*-value (22). Given the statistical power involved, this assessment was expected to yield the most reliable and precise estimates.

#### Assessment of small study effects

The presence of small study effects was assessed by Egger’s regression asymmetry test, which could reflect the presence of publication bias, genuine heterogeneity, or chance (23). The presence of small study effects was interpreted as indicating that smaller studies could show different, often larger, effect estimates than large studies.

#### Evaluation of excess significance

We assessed excess significance bias by testing whether the number of studies observed in the published literature with nominally statistically significant results (“positive” studies, *p* < 0.05) differed from the expected number of statistically significant studies (24). The sum of the statistical power estimates, using the noncentral t distribution algorithm, was applied to provide the expected number of statistically significant studies in each meta-analysis. The plausible effect size of the tested association determined the power estimates for each composed study and was assumed to be the effect of the largest study in each meta-analysis. Sensitivity analyses were then performed using the generalized fixed and random effects estimates as alternative plausible effect sizes. *p* ≤ 0.10 was considered as statistical evidence of the presence of excess significance for an individual meta-analysis.

#### Assessment of credibility ceilings

To address potential methodological limitations in observational studies, which may result in misleading precision of combined effect estimates, we conducted an assessment of credibility ceilings (25, 26). The core assumption of this approach is that each observational study has a probability c (the credibility ceiling) that the true effect estimate differs in direction from the point estimate. We re-estimated the pooled effect size and the heterogeneity among studies across a range of credibility ceiling values.

### Quality assessment

The methodological quality of the included systematic reviews was assessed using the A Measurement Tool to Assess Systematic Reviews (AMSTAR) 2 tool (27). It consists of 16 items, each of which was rated as “yes”, “partial yes”, or “no” according to the AMSTAR 2 guidance. The critical domains included a clear protocol, a comprehensive literature search, reasons for exclusion, risk of bias assessment, appropriate methods for meta-analysis, interpretation of risk of bias, and assessment of publication bias. Based on the guidance, reviews were classified as high, moderate, low, or critically low quality.

### Evidence classification

The associations between PDs and site-specific cancers were categorized into convincing (class I), highly suggestive (class II), suggestive (class III), weak (class IV), or not significant (class V) based on the strength and validity of the evidence, heterogeneity assessment, sample size, and bias assessment (**Table 1**) (28, 29).

**Table 1:** Level of evidence for grading levels.

Criteria	Convincing (class I)	Highly suggestive (class II)	Suggestive (class III)	Weak (class IV)	No significance (class V)
*p*-value from REM	< 10^−6^	< 10^−6^	< 10^−3^	< 0.05	> 0.05
Sample size	> 1,000 cases	> 1,000 cases	> 1,000 cases	–	–
*p*-value from the largest study	< 0.05	< 0.05	–	–	–
Heterogeneity (*I*^2^)	< 50%	–	–	–	–
95% prediction interval (PI)	Excludes the null	–	–	–	–
Small study effects	Not detected	–	–	–	–
Excess significance bias	Not detected	–	–	–	–
10% credibility ceiling	Excludes the null	–	–	–	–

*REM*, randomized effect model.

## Results

### Characteristics of included studies

**Figure 1:**
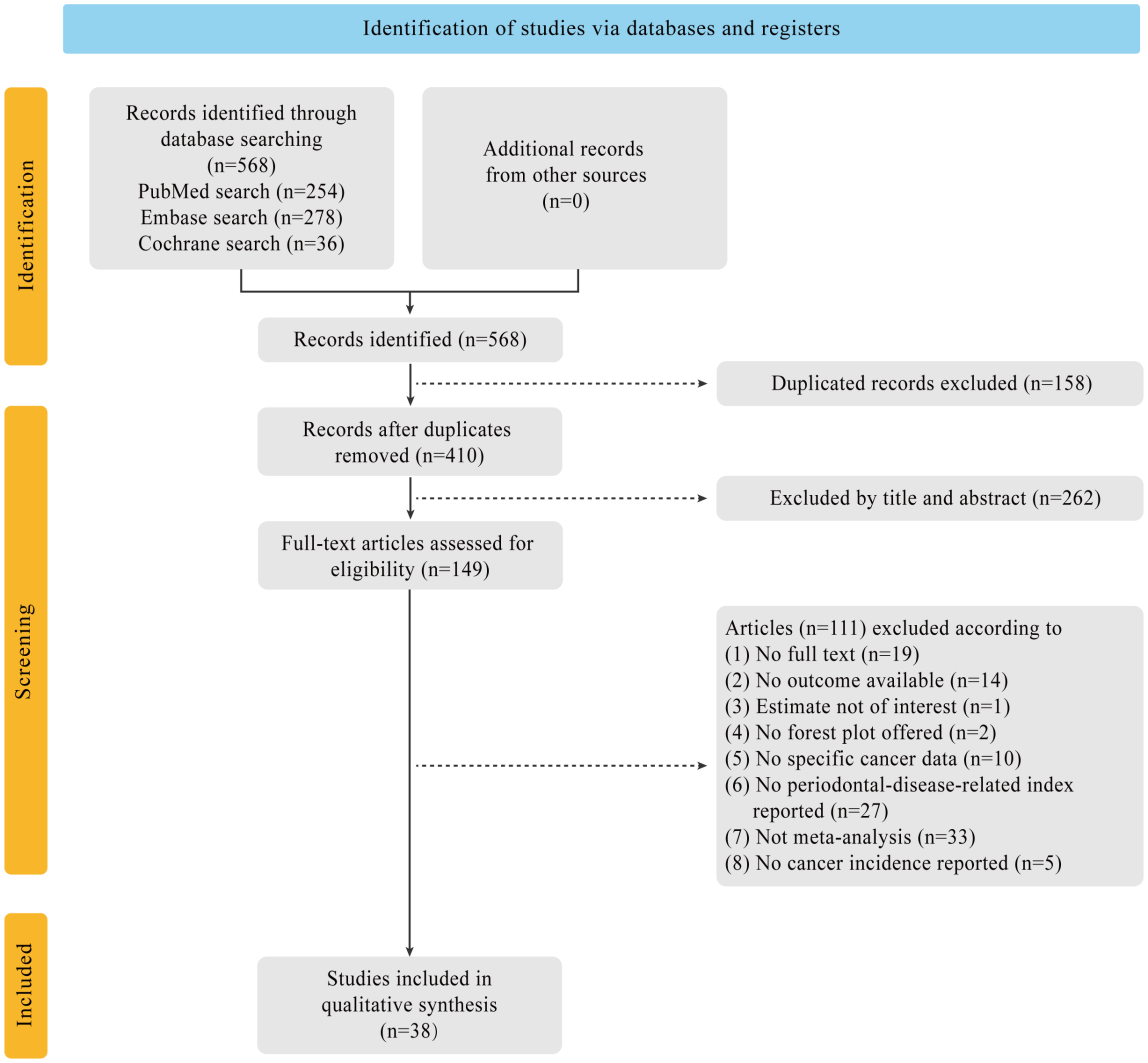
Flowchart of study selection. Overview of the literature search and study selection process, including record identification, screening, full-text assessment, and final inclusion of eligible meta-analyses in the umbrella review.

The study selection process is summarized in **Figure 1**. In total, 568 articles were identified from three databases up to June 2026. After removal of 158 duplicates, 410 unique articles underwent title and abstract screening, of which 261 were excluded. The remaining 149 articles were assessed as full-texts, resulting in the exclusion of 111 articles for predefined reasons. Finally, 38 eligible articles were incorporated, yielding 65 synthesized effect estimates. The included studies were published between 2013 and 2026. All 65 associations were recalculated and analyzed. Following assessment of CCA and application of predefined selection criteria, 23 independent associations were identified for the primary analyses and credibility evaluation.

In total, 14 specific cancer types were included in the study, including head and neck cancers (HNC) (six articles, 16%), oral cancer (seven articles, 18%), lung cancer (eight articles, 21%), breast cancer (four articles, 11%), esophageal cancer (three articles, 8%), gastric cancer (four articles, 11%), pancreatic cancer (five articles, 13%), liver cancer (two articles, 5%), colorectal cancer (CRC) (eight articles, 21%), bladder cancer (three articles, 8%), kidney cancer (one article, 3%), prostate cancer (eight articles, 21%), melanoma (one article, 3%), and hematopoietic and lymphatic cancers (HLCs) (two articles, 5%). Among those, seven (18%) articles investigated multiple cancer sites with individual synthesized estimates. Of the 38 eligible articles, PDs were the most frequently examined exposure (29 studies, 76%), followed by periodontitis (seven studies, 18%) and ABL (one study, 3%). One study evaluated both PDs and ABL. Thirteen articles (34%) provided explicit diagnostic definitions of PDs, whereas 33 (87%) reported the methods used to ascertain periodontal status. The majority combined clinical examination with self-reported periodontal measures (28 studies, 74%), while only four studies (11%) excluded self-reported assessments. The detailed characteristics of all included studies are presented in **Supplementary Table 3**.

### Overlap assessment and rationale for the author’s own meta-analysis

Given the existence of multiple meta-analyses on the same topic, we conducted CCA to assess overlap. The results were subsequently integrated with methodological quality assessments to determine whether an AOM was required to provide the most comprehensive and up-to-date evidence synthesis.

The detailed citation matrices and CCA calculations are presented in **Supplementary Table 4**. Overall, 17 unique exposure–outcome associations were covered by more than one meta-analysis, including associations of PDs or periodontitis with gastric, lung, colorectal, pancreatic, prostate, liver, esophageal, bladder, breast, oral, and HNC, as well as associations between ABL and HNC.

Among these 17 associations, 15 (88%) demonstrated critically high overlap, indicating that the included meta-analyses largely synthesized the same body of primary evidence. Therefore, the most comprehensive association with the highest methodological quality was selected for the primary analysis, and no AOM was considered necessary. Only two associations showed moderate overlap: periodontitis and pancreatic cancer, and PDs and HNC. Moderate CCA suggested substantial differences in the primary studies included across articles. Consequently, these associations underwent further evaluation, including verification of study eligibility, assessment of methodological quality, and additional evidence synthesis through an AOM.

For the association between periodontitis and pancreatic cancer, two eligible meta-analyses (30, 31) reported consistent findings and demonstrated moderate overlap. Using the higher-quality review (31) as the reference, three additional eligible studies (32–34) that had not been incorporated into the selected meta-analysis were identified and included in the AOM. For the association between PDs and HNC, four eligible meta-analyses (35–38) were identified, all reporting directionally consistent results but exhibiting moderate overlap. Compared with the review with the highest methodological quality (37), nine additional eligible studies (39–47) were identified and subsequently incorporated into the AOM to maximize evidence completeness.

### Description and overview of the results

Among 23 primary associations, the median number of included original studies was seven (range: two to 18). Fourteen associations (61%) pertained to PDs and cancer risk, while eight associations (35%) pertained to periodontitis, and one (4%) to ABL. About 18 associations (78%) were statistically significant (*p*-value < 0.05). At a *p*-value threshold of 10^−3^, 10 (43%) associations remained significant, whereas at a threshold of 10^−6^, three (13%) associations remained significant. High heterogeneity (*I*^2^ ≥ 75%) was identified in seven associations (30%), while moderate heterogeneity (75% ≥ *I*^2^ ≥ 50%) was observed in eight main associations (35%). Furthermore, a statistically significant *p*-value of the largest study was found in 14 associations (61%). The 95% PIs excluded the null value in two associations (9%). No evidence of small study effects was observed in 17 associations (74%). At a 10% credibility ceiling, the null value was excluded in 12 associations (52%). Additionally, no evidence of excess significance bias was observed in six associations (26%).

**Figure 2:**
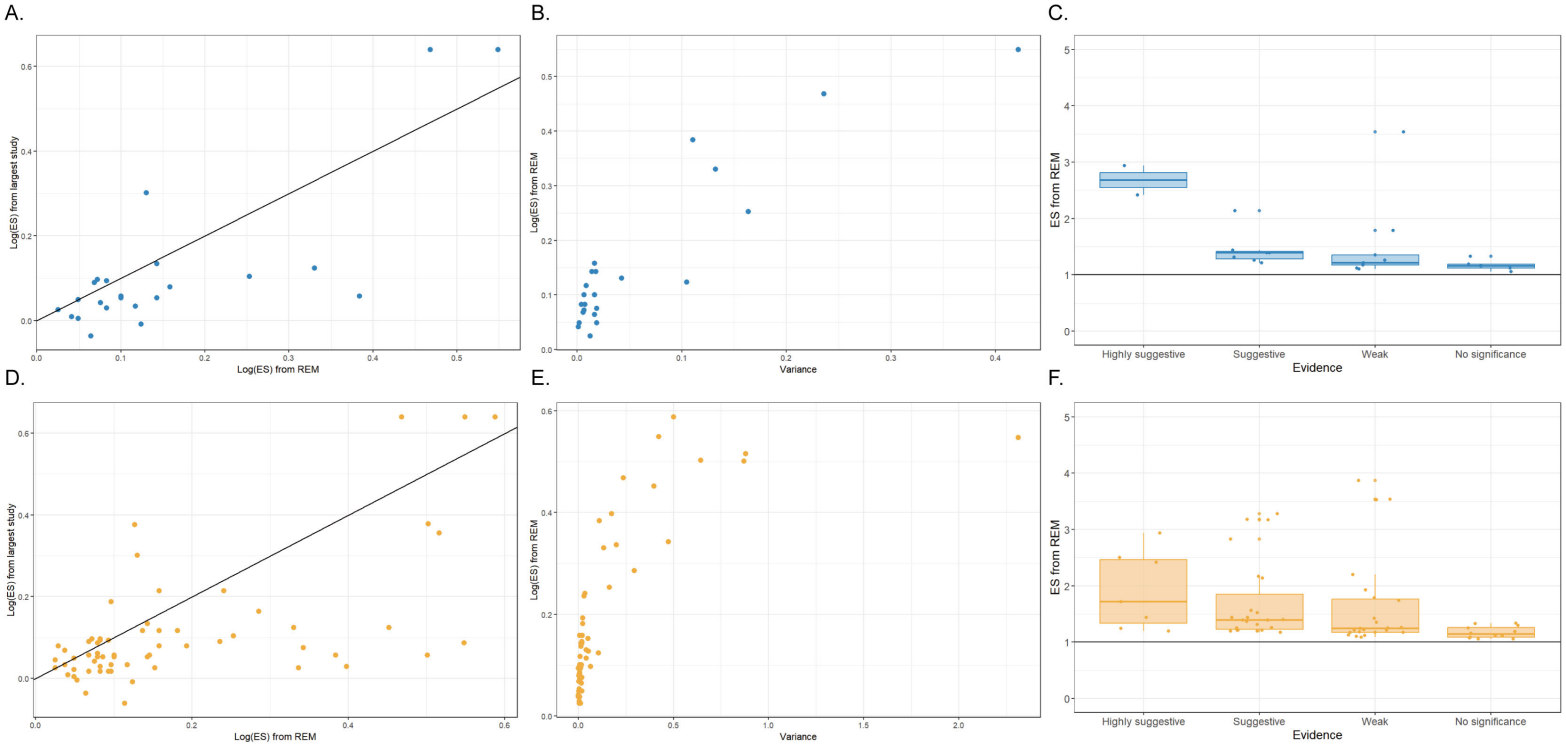
Distribution and robustness of effect estimates across primary and synthesized associations. **(A)** Comparison between the logarithm-transformed effect estimates from the largest individual study and the corresponding random-effects meta-analysis estimates for the 23 primary associations. The diagonal line indicates equality between the two estimates. **(B)** Relationship between the variance of the pooled estimate and the logarithm-transformed random-effects summary effect size for the 23 primary associations. **(C)** Distribution of effect sizes across evidence credibility categories among the 23 primary associations. Boxes represent the interquartile range (IQR), center lines indicate medians, and points denote individual associations. **(D)** Comparison between the logarithm-transformed effect estimates from the largest individual study and the corresponding random-effects summary estimates for all 65 synthesized associations. The diagonal line represents equality between the two estimates. **(E)** Relationship between the variance of the pooled estimate and the logarithm-transformed random-effects summary effect size for all 65 synthesized associations. **(F)** Distribution of effect sizes according to evidence credibility categories across all 65 synthesized associations. Boxes represent the IQR, center lines indicate medians, and points correspond to individual associations. ES, estimated size; REM, randomized effect model.

The estimates derived from the largest studies tended to be more conservative than those from random-effects estimates. Among the 23 primary associations, 14 (61%) produced pooled estimates exceeding those of the largest individual study (**Figure 2A**), while summary effect sizes ranged from 1.06 to 3.54 (**Figure 2B**). Similar patterns were observed across all 65 synthesized associations: 47 (72%) yielded summary estimates exceeding those from the largest study, with random-effects magnitudes ranging from 1.06 to 3.87 (**Figures 2D, E**). These findings suggest that pooled estimates were frequently influenced by smaller studies reporting stronger associations and therefore warrant careful credibility assessment. The resulting evidence-level distributions for the 23 primary associations and all 65 synthesized associations are presented in **Figures 2C, F**, respectively. A descriptive overview of all 65 synthesized associations extracted from the eligible reviews is presented in **Supplementary Table 5**.

### Strength of epidemiological evidence

**Figure 3:**
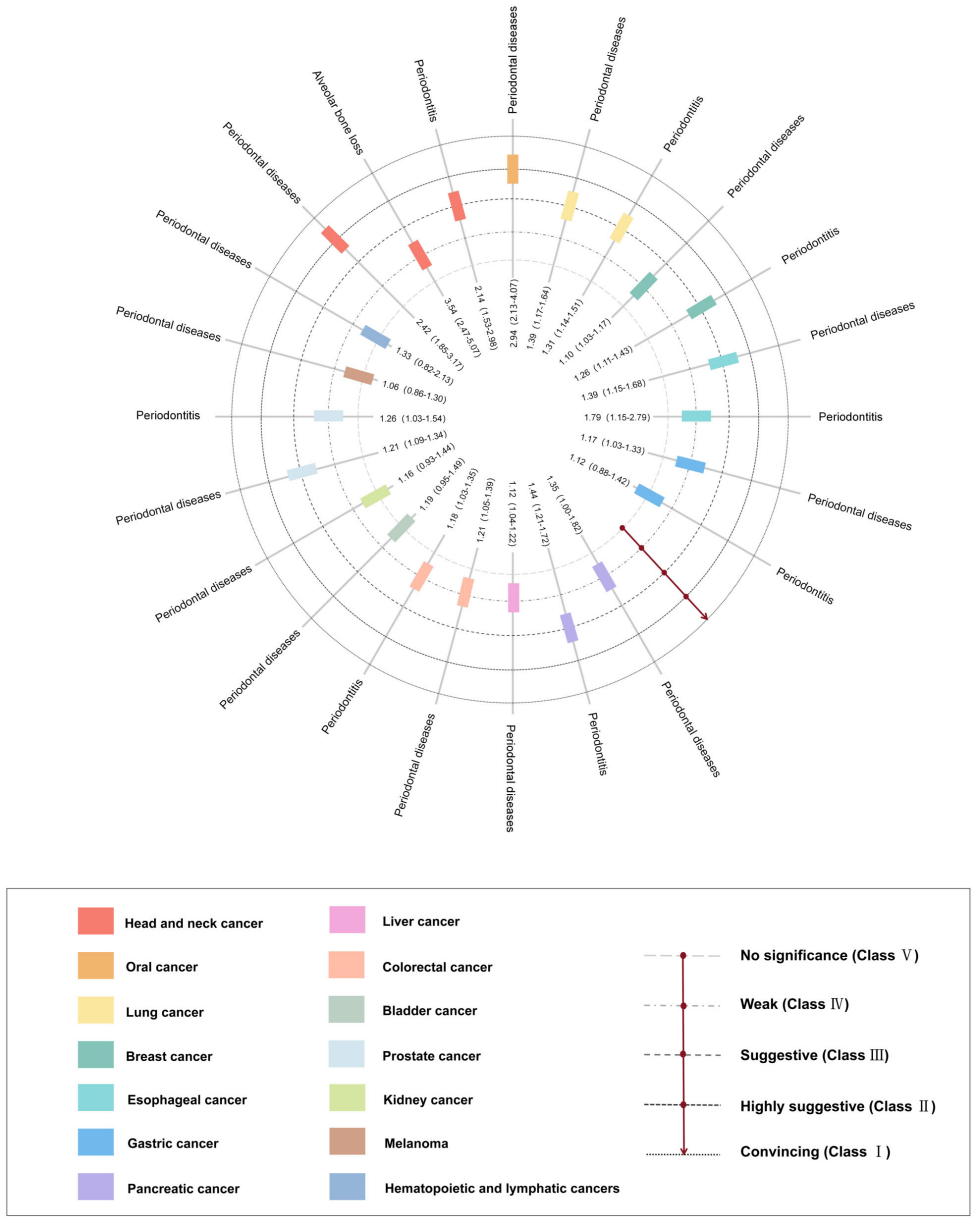
Evidence map of the main associations between periodontal diseases and site-specific cancer. Evidence map summarizing the magnitude, direction, and credibility of associations between periodontal conditions and site-specific cancer risk. Each bar represents a pooled effect estimate with its 95% confidence interval, color-coded according to cancer type. Radial position indicates the evidence credibility level, ranging from convincing to nonsignificant.

Among 23 primary associations, two associations (9%) were classified as highly suggestive, and seven (30%) were classified as suggestive evidence. Nine associations (39%) were classified as weak evidence, while five (22%) associations were ranked as not significant. The summary of primary evidence is illustrated in **Figure 3** and **Supplementary Figure 2**.

Among 14 associations investigating PDs and site-specific cancer risk, two highly suggestive pieces of evidence were identified in HNC (OR, 2.42; 95% CI, 1.85–3.17) and oral cancer (OR, 2.94; 95% CI, 2.13–4.07). Additionally, three pieces of suggestive evidence suggested a positive link in lung (HR, 1.39; 95% CI, 1.17–1.64), esophageal (HR, 1.39; 95% CI, 1.15–1.68), and prostate cancer (HR, 1.21; 95% CI, 1.09–1.34). Another five associations were considered weak evidence, encompassing breast (RR, 1.10; 95% CI, 1.03–1.17), gastric (RR, 1.17; 95% CI, 1.03–1.33), pancreatic (HR, 1.35; 95% CI, 1.00–1.82), liver (RR, 1.12; 95% CI, 1.04–1.22), and CRC (HR, 1.21; 95% CI, 1.05–1.39). Additionally, four associations in bladder (HR, 1.19; 95% CI, 0.95–1.49), kidney (RR, 1.16; 95% CI, 0.93–1.44), melanoma (RR, 1.06; 95% CI, 0.86–1.30), and HLC (HR, 1.33; 95% CI, 0.82–2.13) failed to show statistical significance.

In total, eight associations investigated periodontitis and cancer risk. Four significant associations in HNC (RR, 2.14; 95% CI, 1.53–2.98), lung (RR, 1.31; 95% CI, 1.14–1.51), breast (HR, 1.26; 95% CI, 1.11–1.43), and pancreatic cancer (HR, 1.44; 95% CI, 1.21–1.72) were supported with suggestive evidence. Weak evidence was found in three cancer types, including esophageal (HR, 1.79; 95% CI, 1.15–2.79), CRC (HR, 1.18; 95% CI, 1.03–1.35), and prostate cancer (HR, 1.26; 95% CI, 1.03–1.54). Additionally, no significance was discovered in gastric cancer (HR, 1.12; 95% CI, 0.88–1.42). For the association between ABL and HNC, statistically significant evidence was identified, supported by weak evidence classification (OR, 3.54; 95% CI, 2.47–5.07).

### Subgroup analysis

When stratified by study design, 35 associations were extracted from eligible meta-analyses, yielding four associations with convincing evidence, three with highly suggestive evidence, two with suggestive evidence, 11 with weak evidence, and 14 with no significant evidence (**Supplementary Table 6**). When analyses were restricted to cohort studies, three associations were supported by convincing evidence, including the associations of PDs with lung and prostate cancer. When analyses were restricted to case-control studies, only one convincing piece of evidence supported the association between periodontitis and lung cancer. When exploring the risk of CRC, the case-control group showed highly suggestive evidence, while the corresponding cohort group failed to show statistical significance.

Regional subgroup analyses identified 83 associations in total (**Supplementary Table 7**). Convincing evidence was observed in the association between PDs and prostate cancer in Asian populations and between PDs and lung cancer in American populations. In American populations, three highly suggestive associations were identified, mainly for lung cancer in individuals with PDs or periodontitis. Evidence from European populations was more limited, with only one highly suggestive association observed between PDs and lung cancer.

After restricting analyses to studies adjusted for both smoking and alcohol consumption, 24 associations were identified (**Supplementary Table 8**). After adjusting, five associations remained supported by highly suggestive evidence, primarily involving increased risks of HNC, oral, lung, and prostate cancers among individuals with PDs.

Both self-report and clinical examination were adopted when conducting the meta-analysis. When stratified by the method of evaluating periodontal condition, 29 associations were found (**Supplementary Table 9**). One convincing piece of evidence supported the association between PDs and prostate cancer in the clinical examination group, while the corresponding self-report group was supported with weak evidence. Moreover, one highly suggestive piece of evidence supported the association between periodontitis and lung cancer in the self-report group, while the corresponding clinical examination group showed weak evidence.

When stratified by follow-up duration, 20 associations were identified (**Supplementary Table 10**). A highly suggestive association between PDs and breast cancer was observed in studies with shorter follow-up durations. However, this association was not statistically significant in studies with longer follow-up periods.

### Quality assessment of meta-analyses

The AMSTAR 2 tool was applied to assess the methodological quality of the meta-analyses. Among 38 studies, 11 (29%) were rated as high, while three (8%) were classified as moderate. Fifteen (39%) studies were considered as low, and nine (24%) were determined as critically low. Among seven critical domains, a lack of a preregistered protocol (*n* = 22, 58%) was the main reason for noncompliance. Additionally, 19 (50%) articles failed to assess the potential impact of risk of bias in individual studies. Detailed evaluation of each meta-analysis is presented in **Supplementary Figure 3** and **Supplementary Table 11**.

## Discussion

### Current evidence and mechanisms

To our knowledge, this is the first umbrella review to systematically summarize and critically appraise the current epidemiological evidence on the association between PDs and the risk of 14 site-specific cancers. Among the 23 primary associations, only two associations were supported by highly suggestive evidence. These meta-analyses provided highly suggestive evidence supporting an association between PDs and both HNC and oral cancer and were consistent with the findings reported by Nwizu et al. (48). For the remaining 12 cancer types, current epidemiological evidence was insufficient to support robust associations with PDs, with most associations supported only by suggestive or weak evidence.

The oral cavity harbors the second most diverse microbial ecosystem in the human body (49), and plays an essential role in maintaining oral microbial homeostasis. Chronic inflammation is widely acknowledged as a key factor in the development of cancer hallmarks (50). Taken together, several mechanisms have been proposed to account for the positive associations identified in our review.

**Figure 4:**
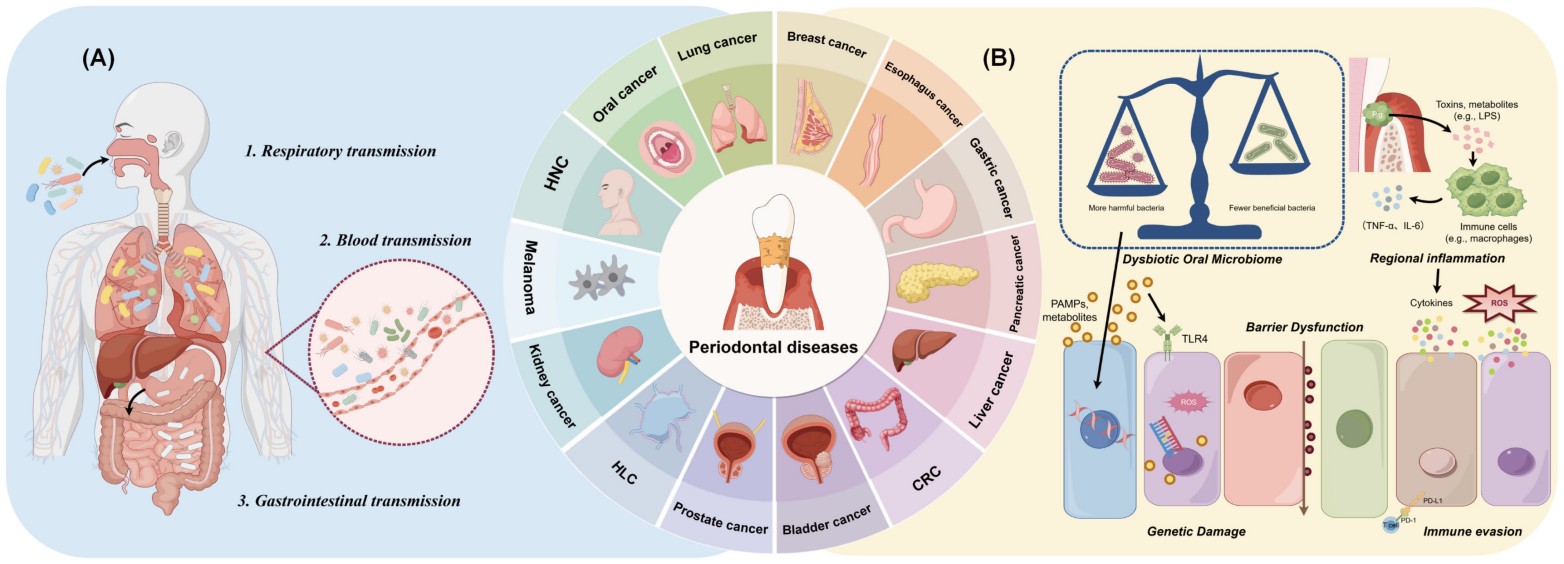
Mechanistic evidence of periodontal diseases and site-specific cancers. **(A)** Primary transmission routes of oral microbiota. **(B)** Inflammation-driven oncogenesis induced by dysbiotic oral microbiota. HNC, head and neck cancer; CRC, colorectal cancer; HLC, hematopoietic and lymphatic cancer; IL-6, interleukin-6; LPS, lipopolysaccharide; PAMPs, pathogen-associated molecular patterns; TLR4, Toll-like receptor 4; TNF-α, tumor necrosis factor-alpha; ROS, reactive oxygen species; D-L1, programmed death-ligand 1.

The pathogenic effect of the oral microbiome may extend beyond the oral cavity and influence distant organs via aspiration (36), hematogenous dissemination (51), and ingestion (52), thereby potentially contributing to carcinogenesis at distant sites (**Figure 4A**). Scannapieco and colleagues speculated that bronchitis and other respiratory infections may be partially attributable to the aspiration of oral pathogens (53). A study also indicated that oral bacteria constitute the main source of lung pathogens, providing support for this hypothesis (54). Furthermore, Abed and colleagues, using a mouse model, demonstrated that oral bacteria enter circulation through ulcerated periodontal pockets, potentially leading to transient bacteremia and subsequent gut colonization (51). Regarding the ingestion route, successful colonization depends on several permissive host factors (e.g., impaired gastric acid barrier or disruption of intestinal mucosal integrity) (55). Even with repeated microbial exposure via dietary intake, successful intestinal colonization still requires conducive host conditions.

Oral bacteria have also been hypothesized to contribute directly to carcinogenesis. Fusobacterium nucleatum has been shown to engage Toll-like receptor 4 (TLR4), activating the NF-κB signaling pathway and increasing the production of pro-inflammatory cytokines (e.g., IL-1β, tumor necrosis factor-alpha [TNF-α]) (56). Concurrently, it also binds T-cell immunoreceptor with Ig and ITIM domains (TIGIT) and carcinoembryonic antigen-related cell adhesion molecule 1 (CEACAM1), suppressing natural killer (NK) cell cytotoxicity and T-cell activation—potentially contributing to chronic inflammation and immune evasion (56). Similarly, *Porphyromonas gingivalis* has been reported to interfere with apoptotic signaling pathways by disrupting mitochondrial intrinsic apoptosis and blocking caspase cascade activation (57).

Interaction between chronic inflammation and immune dysregulation is thought to play an important role in carcinogenesis (**Figure 4B**). As well-elucidated in various studies, these elevated inflammatory mediators may contribute to carcinogenesis by stimulating oncogenic mutations, producing tumor-promoting mediators, inducing tumor cell proliferation, and activating tumor angiogenesis (58). Oral microbial dysbiosis—characterized by pathogenic overgrowth and release of pathogen-associated molecular patterns (PAMPs)—may elicit chronic inflammation through activation of host receptors, potentially leading to oxidative stress and genetic damage (59). This inflammatory milieu, sustained by pro-inflammatory cytokines (TNF-α, IL-6) and reactive oxygen species (ROS) (60), may facilitate malignant transformation by enhancing proliferative signaling, suppressing apoptotic pathways, and compromising epithelial barrier integrity. Increased mucosal permeability may facilitate microbial translocation into deeper tissues, exacerbating tissue damage and potentially contributing to an immunosuppressive microenvironment in which tumor cells may evade immune surveillance through upregulation of checkpoint ligands (e.g., PD-L1) (61). Collectively, these mechanisms provide biologically plausible explanations for the observed association between PDs and cancers.

Beyond inflammation and the direct effect of microbial invasion, oral microorganisms may also contribute through the production of carcinogenic metabolites, disrupting cellular homeostasis and cellular physiology (62). Ethanol-metabolizing oral microorganisms can convert alcohol into acetaldehyde, a genotoxic substance (53). Likewise, nitrate-reducing oral microorganisms may increase endogenous nitrosamine formation (63).

### Sources of heterogeneity and confounding

Substantial heterogeneity was observed across the majority of our results. One potential explanation is the considerable variability in the definition and assessment of PDs across primary studies. PD was defined using diverse criteria, including periodontitis and ABL. These measures capture different dimensions of periodontal destruction and may therefore identify distinct populations, limiting comparability across studies and contributing to between-study heterogeneity. Furthermore, inconsistent definitions of PDs may lead to the classification of individuals with different degrees of periodontal destruction into the same exposure category, potentially obscuring severity-specific associations and attenuating the true relationship between PDs and cancer risk. In addition, periodontal status was assessed using both clinical examinations and self-reported measures. Although self-reported measurement of PDs has been validated for its reliability in numerous studies ranging from systematic reviews to validation studies (64–66), certain constraints remain difficult to overcome. Restricted to education level, self-reported PDs are associated with inadequate and inaccurate assessment of periodontal condition and severity, which may undermine the potential association between PDs and cancers. According to the subgroup analysis in the assessment method, no clear differences were observed for HNC or oral cancer. However, this finding should be interpreted with caution due to the limited number of available studies. Future studies should adopt standardized periodontal diagnostic criteria and consistently report assessment-specific findings to improve comparability across studies and facilitate more robust evidence synthesis.

Smoking and alcohol consumption represent the major sources of confounding in studies investigating PDs and cancers, since they are considered to share common pathways with PDs and carcinogenesis (67, 68). Pooled analyses from the INHANCE Consortium (69) and subsequent meta-analyses (70, 71) have consistently demonstrated strong independent and synergistic effects of tobacco and alcohol exposure on cancer risk. Beyond that, recent research indicated that smoking played a crucial role in changing the composition and abundance of oral microbes since an accumulation of pathogenic microbes, like *Treponema denticola* and *Actinomyces graevenitzii*, was detected in smokers, highlighting the existence of a balance disruption in the oral microenvironment (72, 73). Given that, we further conducted subgroup analyses. Consistent with previous findings (74), highly suggestive evidence for the associations between PDs and both HNC and oral cancer remained even after adjustment. These findings indicate that the observed associations are unlikely to be fully explained by tobacco and alcohol exposure alone. Nevertheless, these results should be interpreted with caution, as adjustment strategies varied across studies. These underscore the need for future large-scale prospective studies with a comprehensive assessment of these confounders to further clarify the independent contribution of PDs to carcinogenesis.

Follow-up duration may represent another important source of heterogeneity. Given the long latency period of carcinogenesis, insufficient follow-up may fail to capture the cumulative effects of chronic periodontal inflammation on cancer development. However, evidence regarding the impact of follow-up duration remains limited. Among the included meta-analyses, only one study performed subgroup analyses according to follow-up length, and no convincing or highly suggestive associations were identified in studies with longer follow-up periods. Nevertheless, this study was rated as having low methodological quality according to AMSTAR 2, which limits confidence in the findings. Therefore, the extent to which follow-up duration influences the observed associations remains uncertain. The scarcity of follow-up-specific analyses highlights an important evidence gap. Future prospective studies with adequate follow-up are warranted to better characterize the temporal relationship between PDs and cancer risk.

Apart from the aforementioned reasons, in the HNC group, a lack of subsite analysis may also lead to high heterogeneity. HNC is cancer arising from the oral cavity, pharynx, and larynx. Except for oral cancer, it was difficult to conduct further exploration of associations between PDs and pharyngeal cancer as well as laryngeal cancer, due to data insufficiency.

### Current stance and future challenges

Our review provides suggestive evidence for future research investigating the association between PDs and cancer. The use of diverse periodontal definitions and assessment methods has hindered the development of standardized clinical tools and contributed substantially to heterogeneity (75, 76). To standardize the assessment of PDs, objective and stable indicators that remain unchanged and irreversible, like ABL, CAL, and the community periodontal treatment needs index (CPITN), should be applied (77). Future studies should adopt standardized periodontal diagnostic criteria and incorporate objective clinical measurements, longitudinal assessments, and mechanistic biomarkers to improve exposure characterization and clarify the role of PDs in carcinogenesis.

After reviewing existing findings, an interesting assumption arose: whether timely interventional treatment could mitigate the elevated cancer risk associated with PDs remains unclear. Furthermore, whether routine dental care can reduce cancer development remains an important unanswered question. Several studies have reported associations between oral hygiene behaviors, including the use of dental floss and mouthwash products, and oral cancer risk (78). Conversely, a statistically significant association between frequent tooth brushing and a reduced risk of HNC was reported in Sato’s study, especially among smokers and drinkers (79). Similarly, a study from Taiwan revealed that patients who underwent periodontal treatment exhibited a reduced risk of overall cancer, compared to those who did not undergo treatment (80). Although previous studies have reported promising findings, there is still an urgent need for more well-designed, high-quality clinical trials to ascertain the additional benefits of oral interventions.

Growing microbiome research has suggested that specific bacteria may act as key mediators of carcinogenesis. This was particularly salient for the oral-gut axis—a conceptual framework addressing microbial crosstalk between two of the most diverse microbial ecosystems in the human body. Mounting evidence has implicated the effects of oral microorganisms in gastrointestinal malignancies, especially in CRC. Compared with healthy controls, elevated levels of *Fusobacterium nucleatum* and *Porphyromonas gingivalis* were detected in fecal samples of CRC patients (81, 82), through activation of the NF-κB and/or Wnt/β-catenin signaling pathways (56, 83). Considering that the translocation and colonization of oral microorganisms required pre-existing gut dysbiosis (e.g., intestinal disorder, or antibiotic effects) and the breach of gastrointestinal barrier thresholds (84, 85), detection of PD-related microorganisms in feces failed to accurately reflect the condition in the oral cavity. Notably, Rezasoltani et al. reported elevated salivary *Fusobacterium nucleatum* DNA in CRC patients (86), showing potential diagnostic efficacy. Salivary samples captured genuine dysbiosis in the oral cavity while controlling for confounding from gut-derived specimens. However, a paucity of such studies precluded quantitative synthesis through meta-analyses. We therefore urge larger-scale cohorts to assess the value of oral microorganisms sampled directly from saliva as diagnostic biomarkers and thus further elucidate the mechanism of the oral-gut axis.

### Limitations

Several limitations should be considered in this umbrella review. Firstly, subgroup analyses based on covariate adjustment were limited due to various adjustment strategies across studies. Consequently, smoking and alcohol consumption remain important shared risk factors for PDs and cancers. Secondly, substantial variability existed in PD’s definition and assessment, which may introduce exposure misclassification and obscure potential severity-specific associations. Thirdly, in order to maximize the comprehensiveness of the evidence base, cross-sectional studies were included and constituted the majority of studies rather than cohort studies. The strength of evidence may have been overestimated given the characteristics of observational studies. Fourth, despite a comprehensive literature search being conducted, updated observational studies that have not been synthesized in any meta-analyses may exist. However, given the volume of included studies, it appears unlikely to dramatically affect the overall findings in this study. Furthermore, nine primary pieces of evidence were based on low/critically low-quality articles, mainly due to protocol registration. This methodological weakness may affect the credibility assessments in this umbrella review. Therefore, these findings should be interpreted with greater caution.

## Conclusion

This umbrella review demonstrates that PDs are associated with an increased risk of several site-specific cancers, and the strength of evidence varies across cancer types. Highly suggestive evidence was observed for HNC and oral cancer. Given the high global burden and modifiable nature of PDs, these findings support closer integration of periodontal care into multidisciplinary cancer prevention and population health strategies.

## Data Availability

The original contributions presented in the study are included in the article/**Supplementary Material**. Further inquiries can be directed to the corresponding authors.
