# Supplementary material for: Risk of cancer in periodontal disease: an umbrella review of meta-analyses
Source: Front Immunol. 2026 Jul 10;17:1839715. doi: 10.3389/fimmu.2026.1839715 (PMC13395657; doi:10.3389/fimmu.2026.1839715)

# Supplementary Materials

**CONTENT**

**STable 1. PRISMA checklist.**

**STable 2. The detailed search strategy for all three databases incl. search terms used for the systematic literature searches.**

**STable 3. Characteristics of included meta-analyses.**

**STable 4. Assessment of corrected covered area.**

**STable 4.1 Corrected covered area on periodontal diseases and gastric cancer**

**STable 4.2 Corrected covered area on periodontal diseases and lung cancer**

**STable 4.3 Corrected covered area on periodontitis and lung cancer**

**STable 4.4 Corrected covered area on periodontal diseases and colorectal cancer**

**STable 4.5 Corrected covered area on periodontitis and colorectal cancer**

**STable 4.6 Corrected covered area on periodontal diseases and pancreatic cancer**

**STable 4.7 Corrected covered area on periodontitis and pancreatic cancer**

**STable 4.8 Corrected covered area on periodontal diseases and prostate cancer**

**STable 4.9 Corrected covered area on periodontitis and prostate cancer**

**STable 4.10 Corrected covered area on periodontal diseases and liver cancer**

**STable 4.11 Corrected covered area on periodontal diseases and esophageal cancer**

**STable 4.12 Corrected covered area on periodontal diseases and bladder cancer**

**STable 4.13 Corrected covered area on periodontal diseases and breast cancer**

**STable 4.14 Corrected covered area on periodontal diseases and head and neck cancer**

**STable 4.15 Corrected covered area on alveolar bone loss and head and neck cancer**

**STable 4.16 Corrected covered area on periodontal diseases and oral cancer**

**STable 4.17 Corrected covered area on periodontal diseases and hematopoietic and lymphatic cancers**

**STable 5. Recalculation and evaluation of the associations between periodontal diseases and cancer risk.**

**STable 6. Subgroup analysis of periodontal diseases and cancer stratified by study design.**

**STable 7. Subgroup analysis of periodontal diseases and cancer stratified by region.**

**STable 8. Subgroup analysis of periodontal diseases and cancer stratified by adjustment variables.**

**STable 9. Subgroup analysis of periodontal diseases and cancer stratified by the method used to evaluate periodontal diseases.**

**STable 10. Subgroup analysis of periodontal diseases and cancer stratified by follow-up duration.**

**STable 11. AMSTAR-2 assessment of 38 eligible articles.**

**SFig 1. Schematic overview of overlap management, author’s own meta-analysis, and primary evidence selection.**

**SFig 2. Characteristics of main associations in periodontal diseases and site-specific cancers.**

**SFig 3. Quality assessment of 38 eligible studies using AMSTAR-2 tool.**

**STable 1. PRISMA checklist.**

| **Section and Topic** | **Item #** | **Checklist item** | **Location where item is reported** |
| --- | --- | --- | --- |
| **TITLE** | | |  |
| Title | 1 | Identify the report as a systematic review. | Page 1 |
| **ABSTRACT** | | |  |
| Abstract | 2 | See the PRISMA 2020 for Abstracts checklist. | Page 2 |
| **INTRODUCTION** | | |  |
| Rationale | 3 | Describe the rationale for the review in the context of existing knowledge. | Page 3 |
| Objectives | 4 | Provide an explicit statement of the objective(s) or question(s) the review addresses. | Page 3,4 |
| **METHODS** | | |  |
| Eligibility criteria | 5 | Specify the inclusion and exclusion criteria for the review and how studies were grouped for the syntheses. | Page 5,6 |
| Information sources | 6 | Specify all databases, registers, websites, organisations, reference lists and other sources searched or consulted to identify studies. Specify the date when each source was last searched or consulted. | Page 5 |
| Search strategy | 7 | Present the full search strategies for all databases, registers and websites, including any filters and limits used. | STable 1 |
| Selection process | 8 | Specify the methods used to decide whether a study met the inclusion criteria of the review, including how many reviewers screened each record and each report retrieved, whether they worked independently, and if applicable, details of automation tools used in the process. | Page 5,6 |
| Data collection process | 9 | Specify the methods used to collect data from reports, including how many reviewers collected data from each report, whether they worked independently, any processes for obtaining or confirming data from study investigators, and if applicable, details of automation tools used in the process. | Page 6 |
| Data items | 10a | List and define all outcomes for which data were sought. Specify whether all results that were compatible with each outcome domain in each study were sought (e.g. for all measures, time points, analyses), and if not, the methods used to decide which results to collect. | Page 6 |
|  | 10b | List and define all other variables for which data were sought (e.g. participant and intervention characteristics, funding sources). Describe any assumptions made about any missing or unclear information. | Page 6 |
| Study risk of bias assessment | 11 | Specify the methods used to assess risk of bias in the included studies, including details of the tool(s) used, how many reviewers assessed each study and whether they worked independently, and if applicable, details of automation tools used in the process. | Page 9 |
| Effect measures | 12 | Specify for each outcome the effect measure(s) (e.g. risk ratio, mean difference) used in the synthesis or presentation of results. | Page 7 |
| Synthesis methods | 13a | Describe the processes used to decide which studies were eligible for each synthesis (e.g. tabulating the study intervention characteristics and comparing against the planned groups for each synthesis (item #5)). | Page 7 |
|  | 13b | Describe any methods required to prepare the data for presentation or synthesis, such as handling of missing summary statistics, or data conversions. | Page 7 |
|  | 13c | Describe any methods used to tabulate or visually display results of individual studies and syntheses. | Page 8 |
|  | 13d | Describe any methods used to synthesize results and provide a rationale for the choice(s). If meta-analysis was performed, describe the model(s), method(s) to identify the presence and extent of statistical heterogeneity, and software package(s) used. | Page 9 |
|  | 13e | Describe any methods used to explore possible causes of heterogeneity among study results (e.g. subgroup analysis, meta-regression). | Page 7 |
|  | 13f | Describe any sensitivity analyses conducted to assess robustness of the synthesized results. | Page 8,9 |
| Reporting bias assessment | 14 | Describe any methods used to assess risk of bias due to missing results in a synthesis (arising from reporting biases). | Page 9 |
| Certainty assessment | 15 | Describe any methods used to assess certainty (or confidence) in the body of evidence for an outcome. | Table 1 |
| **RESULTS** | | |  |
| Study selection | 16a | Describe the results of the search and selection process, from the number of records identified in the search to the number of studies included in the review, ideally using a flow diagram. | Page 10 |
|  | 16b | Cite studies that might appear to meet the inclusion criteria, but which were excluded, and explain why they were excluded. | Figure 2 |
| Study characteristics | 17 | Cite each included study and present its characteristics. | STable 2 |
| Risk of bias in studies | 18 | Present assessments of risk of bias for each included study. | STable 5 |
| Results of individual studies | 19 | For all outcomes, present, for each study: (a) summary statistics for each group (where appropriate) and (b) an effect estimate and its precision (e.g. confidence/credible interval), ideally using structured tables or plots. | STable 4 |
| Results of syntheses | 20a | For each synthesis, briefly summarise the characteristics and risk of bias among contributing studies. | STable 4 |
|  | 20b | Present results of all statistical syntheses conducted. If meta-analysis was done, present for each the summary estimate and its precision (e.g. confidence/credible interval) and measures of statistical heterogeneity. If comparing groups, describe the direction of the effect. | Figure 3 |
|  | 20c | Present results of all investigations of possible causes of heterogeneity among study results. | STable 4 |
|  | 20d | Present results of all sensitivity analyses conducted to assess the robustness of the synthesized results. | STable 4 |
| Reporting biases | 21 | Present assessments of risk of bias due to missing results (arising from reporting biases) for each synthesis assessed. | STable 4 |
| Certainty of evidence | 22 | Present assessments of certainty (or confidence) in the body of evidence for each outcome assessed. | Figure 3 |
| **DISCUSSION** | | |  |
| Discussion | 23a | Provide a general interpretation of the results in the context of other evidence. | Page 16,17 |
|  | 23b | Discuss any limitations of the evidence included in the review. | Page 20,21 |
|  | 23c | Discuss any limitations of the review processes used. | Page 23 |
|  | 23d | Discuss implications of the results for practice, policy, and future research. | Page 21,22 |
| **OTHER INFORMATION** | | |  |
| Registration and protocol | 24a | Provide registration information for the review, including register name and registration number, or state that the review was not registered. | Page 4 |
|  | 24b | Indicate where the review protocol can be accessed, or state that a protocol was not prepared. | Page 4 |
|  | 24c | Describe and explain any amendments to information provided at registration or in the protocol. | Non-applicable |
| Support | 25 | Describe sources of financial or non-financial support for the review, and the role of the funders or sponsors in the review. | Page 24 |
| Competing interests | 26 | Declare any competing interests of review authors. | Page 24 |
| Availability of data, code and other materials | 27 | Report which of the following are publicly available and where they can be found: template data collection forms; data extracted from included studies; data used for all analyses; analytic code; any other materials used in the review. | Page 24 |

**STable 2. The detailed search strategy for all three databases incl. search terms used for the systematic literature searches**

| **Database** | **Search Term** | **Result** |
| --- | --- | --- |
| **PubMed** | #1: (periodontal diseas*[Title/Abstract]) OR (gingival diseas*[Title/Abstract]) OR (gingivitis[Title/Abstract]) OR (periodontitis[Title/Abstract]) OR (periodontal inflammation[Title/Abstract]) OR (furcation defects[Title/Abstract]) OR (probing depth[Title/Abstract]) OR (clinical attachment loss[Title/Abstract]) OR (CAL[Title/Abstract]) OR (bleeding on probing[Title/Abstract]) OR (BOP[Title/Abstract]) OR (subgingival calculus[Title/Abstract]) OR (SDCI[Title/Abstract]) OR (plaque index[Title/Abstract]) OR (gingival bleeding index[Title/Abstract]) OR (GBI[Title/Abstract]) OR (gingival index[Title/Abstract]) OR (periodontal pocket*[Title/Abstract]) OR (tooth mobility[Title/Abstract]) OR (chronic periodontitis[Title/Abstract]) OR (aggressive periodontitis[Title/Abstract]) OR (necrotizing ulcerative periodontitis[Title/Abstract]) OR (juvenile periodontitis[Title/Abstract]) OR (adult periodontitis[Title/Abstract]) OR (rapidly progressive periodontitis[Title/Abstract]) OR (diabetic periodontitis[Title/Abstract]) OR (periodontal attachment loss[Title/Abstract]) | 93,853 |
|  | #2: (((((cancer[Title/Abstract]) OR (carcinoma[Title/Abstract])) OR (tumor[Title/Abstract])) OR (adenomas[Title/Abstract])) OR (neoplas*[Title/Abstract])) OR (maligna*[Title/Abstract]) | 4,371,580 |
|  | #3: #1 AND #2 | 6,602 |
|  | #4: (((((systematic review[Publication Type]) OR (meta analysis[Publication Type])) OR (meta-analysis[Publication Type])) OR (systematic review[Title/Abstract])) OR (meta analysis[Title/Abstract])) OR (meta-analysis[Title/Abstract]) | 593,535 |
|  | #5: #3 AND #4 | 254 |
| **EMBASE** | #1: 'periodontal diseas*':ti,ab OR 'gingival diseas*':ti,ab OR gingivitis:ti,ab OR periodontitis:ti,ab OR 'periodontal inflammation':ti,ab OR 'furcation defect*':ti,ab OR 'probing depth':ti,ab OR 'clinical attachment loss':ti,ab OR CAL:ti,ab OR 'bleeding on probing':ti,ab OR BOP:ti,ab OR 'subgingival calculus':ti,ab OR SDCI:ti,ab OR 'plaque index':ti,ab OR 'gingival bleeding index':ti,ab OR GBI:ti,ab OR 'gingival index':ti,ab OR 'periodontal pocket*':ti,ab OR 'tooth mobility':ti,ab OR 'chronic periodontitis':ti,ab OR 'aggressive periodontitis':ti,ab OR 'necrotizing ulcerative periodontitis':ti,ab OR 'juvenile periodontitis':ti,ab OR 'adult periodontitis':ti,ab OR 'rapidly progressive periodontitis':ti,ab OR 'diabetic periodontitis':ti,ab OR 'periodontal attachment loss':ti,ab | 107,287 |
|  | #2: cancer:ab,ti OR carcinoma:ab,ti OR tumor:ab,ti OR adenomas:ab,ti OR neoplas*:ab,ti OR maligna*:ab,ti | 6,113,440 |
|  | #3: #1 AND #2 | 8,768 |
|  | #4: 'systematic review':ab,ti OR 'meta analysis':ab,ti OR 'meta-analysis':ab,ti | 673,899 |
|  | #5: #3 AND #4 | 278 |
| **Cochrane Library** | #1: (periodontal diseas*):ti,ab,kw OR (gingival diseas*):ti,ab,kw OR (gingivitis):ti,ab,kw OR (periodontitis):ti,ab,kw OR (periodontal inflammation):ti,ab,kw OR (furcation defect*):ti,ab,kw OR (probing depth):ti,ab,kw OR (clinical attachment loss):ti,ab,kw OR (CAL):ti,ab,kw OR (bleeding on probing):ti,ab,kw OR (BOP):ti,ab,kw OR (subgingival calculus):ti,ab,kw OR (SDCI):ti,ab,kw OR (plaque index):ti,ab,kw OR (gingival bleeding index):ti,ab,kw OR (GBI):ti,ab,kw OR (gingival index):ti,ab,kw OR (periodontal pocket*):ti,ab,kw OR (tooth mobility):ti,ab,kw OR (chronic periodontitis):ti,ab,kw OR (aggressive periodontitis):ti,ab,kw OR (necrotizing ulcerative periodontitis):ti,ab,kw OR (juvenile periodontitis):ti,ab,kw OR (adult periodontitis):ti,ab,kw OR (rapidly progressive periodontitis):ti,ab,kw OR (diabetic periodontitis):ti,ab,kw OR (periodontal attachment loss):ti,ab,kw | 27,930 |
|  | #2: (cancer):ti,ab,kw OR (carcinoma):ti,ab,kw OR (tumor):ti,ab,kw OR (adenomas):ti,ab,kw OR (neoplas*):ti,ab,kw OR (maligna*):ti,ab,kw | 309,323 |
|  | #3: #1 AND #2 | 1,127 |
|  | #4: (systematic review):ti,ab,kw OR (meta analysis):ti,ab,kw OR (meta-analysis):ti,ab,kw | 32,162 |
|  | #5: #3 AND #4 | 36 |
| **Time** | **20 Jun 2025** | |

**STable 3. Characteristics of included meta-analyses.**

| **Author, year** | **PMID** | **Country** | **Search** | **Risk of bias** | **Total number of original studies/patients** | **Included study design (n)** | **Exposure of interest** | **Cancer type** | **Comparison** | **Effect estimate** | **Definition of PDs/periodontitis** | **Measurement of PD/periodontitis** |
| --- | --- | --- | --- | --- | --- | --- | --- | --- | --- | --- | --- | --- |
| Aguiar, 2024 | 38301538 | Brazil | PubMed, Embase, WoS, Scopus, Lilacs and Opengrey databases ranging from 1961 to 2022 | NOS (6-9) | 9/382,951 | Case-controls (3)  Cohorts (6) | PDs | Gastric cancer | (+) vs (-) | RR | The presence of PDs was considered if one of these clinical conditions occurs: gingivitis, periodontitis and tooth loss | Clinical examination and self-report |
| Bai, 2023 | 36538375 | China | PubMed, Embase, WoS, Cochrane Library from 1977 to 2021 | NOS (3-6) | 44/52,863 | Case-controls (40)  Cohorts (4) | PDs | HNC | (+) vs (-) | OR | - | Clinical examination and self-report. |
| Chen, 2020 | 32802852 | China | PubMed and Embase from 2003 to 2019 | NOS (4-8) | 12/ 224,058 | Case-controls (3)  Cohorts (9) | PDs | Lung cancer | (+) vs (-) | RR | - | Clinical examination and self-report |
| Gopinath, 2020 | 32674369 | Malaysia | PubMed, Scopus and Embase from 2000 to 2019 | NOS (6-8) | 9/2,564 | Case-controls (9) | PDs | HNC | (+) vs (-) | OR | PDs were defined as the presence of gingivitis and/or periodontitis. | Clinical assessment, self-report, and radiographic assessment. |
|  |  |  |  |  |  |  | Alveolar bone loss |  |  |  |  |  |
| Guo, 2021 | 33650836 | China | Medline, Embase, WoS and Cochrane Library from 2003 to 2019 | NOS (5-9) | 9/440,911 | Cohorts (9) | PDs | Prostate cancer | (+) vs (-) | HR | PDs were defined as the presence of periodontitis, tooth loss or gingivitis caused by periodontitis. | Clinical examination, self-report and radiographic parameters. |
| Kesharani, 2022 | 36686995 |  | PubMed, Medline, Embase, Scopus and WoS from 1952 to 2020 | NOS (5-9) | 12/410,510 | Case-controls (4)  Cohorts (8) | Periodontitis | Lung cancer | (+) vs (-) | RR | Periodontitis was defined regardless of the depth and severity of periodontitis. | - |
| Li, 2021 | 33179280 | China | PubMed, Embase, Cochrane Library and WoS from 2003 to 2018 | NOS (4-8) | 7/337,894 | Cohorts (7) | PDs | CRC | (+) vs (-) | RR/HR | Studies involved only tooth loos or oral health instead of PDs were excluded. | Clinical examination (Russell Index, CDC-AAP, and ACH), self-report and medical record. |
| Li, 2022 | 36016605 | China | PubMed, Embase, Cochrane Library from 2003 to 2018 | NOS (4-8) | 11/444,123 | Cohorts (11) | PDs | Urogenital cancer | (+) vs (-) | HR | PDs were defined as periodontitis, gingivitis, periimplantitis. | Clinical examination, self-report and radiographic examinations. |
| Li, 2023 | 37308334 | China | PubMed, Embase, Cochrane Library and WoS from 2003 to 2018 | NOS (4-8) | 12/245,874 | Case-controls (10)  Cohorts (2) | PDs | Oral cancer | (+) vs (-) | OR | PDs were defined as alveolar bone loss, probing pocket depth, clinical attachment loss, bleeding on probing. | Clinical examination and self-report. |
| Ma, 2020 | 33029095 | China | PubMed, Embase, Cochrane Library and WoS from 2008 to 2018 | NOS (6-8) | 10/1,156,131 | Cohorts (10) | Periodontitis | Esophageal cancer | (+) vs (-) | HR | - | Clinical examination and self-report. |
|  |  |  |  |  |  |  |  | Prostate cancer |  |  |  |  |
|  |  |  |  |  |  |  |  | Hematologic malignancy |  |  |  |  |
| Ma, 2024 | 38742908 | China | PubMed, Embase, Cochrane Library and WoS from 2008 to 2018 | NOS (5-9) | 16/31,857 | Case-control (16) | PDs | Oral cancer | (+) vs (-) | OR | PDs were defined as the presence of alveolar bone loss, clinical attachment loss, and tooth loss | Clinical diagnosis (ICD-9-CM) of periodontitis. |
| Mahuili, 2023 | 37398714 | India | PubMed, Embase, Cochrane Library, Scopus and Google Scholar from 1990 to 2021 | NOS (5-9) | 27/20,483 | Case-control (16) | PDs | Oral cancer | (+) vs (-) | OR | PDs were defined as the presence of bleeding gums, mobile teeth, periodontitis, tooth loss, and edentulous state. | - |
| Maisonneuve, 2017 | 28453689 | USA | PubMed, WoS, and Google Scholar from 2003 to 2016 | NR | 8/322,455 | Case-controls (1)  Cohorts (7) | PDs | Pancreatic cancer | (+) vs (-) | RR | PDs were defined as the presence of gingivitis, periodontitis, tooth loss and edentulism. | Clinical measurement and self-report. |
| Shao, 2018 | 30619743 | China | PubMed and CNKI from 2003 to 2018 | NR | 11/173,162 | Case-controls (1)  Cohorts (10) | PDs | Breast cancer | (+) vs (-) | RR | - | Clinical measurement, self-report and medical records. |
| Shi, 2018 | 29974484 | China | PubMed, Cochrane Library, WoS, CBM, CNKI, VIP, Wanfang from 2003 to 2018 | NOS (5-8) | 7/166,283 | Cohorts (7) | PDs | Breast cancer | (+) vs (-) | RR | - | Clinical measurement and self-report. |
| Verma, 2023 | 37090288 | India | PubMed, Science Direct, Medline, Cochrane Library, and Embase from 2010 to 2022 | NOS (5-8) | 7/194,850 | Case-controls (2)  Cohorts (5) | PDs | Lung cancer | (+) vs (-) | RR | - | - |
| Vu, 2021 | 33876587 | Korea | PubMed, Cochrane Library from 2007 to 2019 | NOS (4-7) | 5/1,378 | Case-controls (5) | Alveolar bone loss | HNC | (+) vs (-) | OR | - | Clinical measurement and self-report |
| Wang, 2020 | 32583879 | China | PubMed, Embase, Medline, CENTRAL, and ClinicalTrails.gov from 2003 to 2016 | NOS (6-8) | 8/167,256 | Case-controls (2)  Cohorts (6) | PDs | Lung cancer | (+) vs (-) | HR/OR | - | Clinical diagnosis and medical records. |
| Wang, 2022 | 36389427 | China | PubMed, Embase, WoS, CNKI, and Wanfang from 2003 to 2016 | NOS (6-8) | 27/1,747,729 | Cohorts (27) | Periodontitis | Breast cancer | (+) vs (-) | HR | - | Clinical diagnosis and medical records. |
|  |  |  |  |  |  |  |  | Prostate cancer |  |  |  |  |
|  |  |  |  |  |  |  |  | Lung cancer |  |  |  |  |
|  |  |  |  |  |  |  |  | CRC |  |  |  |  |
| Wang, 2024 | 39185624 | China | PubMed, Embase, WoS, Google Scholar from 2008 to 2023 | NOS (6-8) | 19/16,620,011 | Cohorts (19) | PDs | Esophageal cancer | (+) vs (-) | HR | PD were defined as the presence of gingivitis, periodontitis. Studies with edentulous participants were excluded. | Clinical measurement, self-report and medical records. |
|  |  |  |  |  |  |  |  | CRC |  |  |  |  |
|  |  |  |  |  |  |  |  | Pancreatic cancer |  |  |  |  |
|  |  |  |  |  |  |  |  | Liver cancer |  |  |  |  |
| Wei, 2021 | 33247563 | China | PubMed, Embase, Cochrane Library from 2003 to 2020 | NOS (6-9) | 7/294,399 | Cohorts (7) | PDs | Prostate cancer | (+) vs (-) | RR | - | Clinical measurement, self-report and medical records. |
| Wu, 2020 | 31880294 | China | PubMed, WoS, Medline from 2010 to 2018 | NOS (7-9) | 6/214,004 | Case-controls (1)  Cohorts (5) | PDs | HLC | (+) vs (-) | RR | PDs were defined as the presence of gingivitis, and periodontitis. | Clinical measurement and self-report. |
| Xie, 2018 | 30083109 | China | PubMed, Scopus, ScienceDirect, and CNKI from 2008 to 2017 | NR | 5/298,476 | Cohorts (5) | PDs | Bladder cancer | (+) vs (-) | HR | PDs were defined as the presence of periodontitis. | Clinical measurement and self-reported. |
| Xuan, 2021 | 33269543 | China | PubMed, WoS, CNKI, CBM, Wanfang and VIP from 2003 to 2019 | NOS (6-8) | 13/634,744 | Case-controls (2)  Cohorts (11) | PDs | CRC | (+) vs (-) | RR | PDs were defined as the presence of gingivitis, and periodontitis. | Clinical measurement and self-reported. |
| Yao, 2014 | 24756759 | China | PubMed, Embase, WoS, Wiley Online Library and Wanfang from 2005 to 2010 | Cochrane Handbook 5.0 Quality (NR) | 5/3,183 | Case-controls (5) | PDs | Oral cancer | (+) vs (-) | OR | PDs were defined as the presence of alveolar bone loss, tooth mobility. | Clinical measurement and self-report. |
| Ye, 2016 | 28230025 | China | PubMed and Wanfang from 1990 to 2013 | NR | 11/5,317 | Case-controls (11) | PDs | Oral cancer | (+) vs (-) | OR | - | - |
| Zeng, 2013 | 24194957 | China | PubMed, Embase, and Cochrane Library from 2005 to 2010 | NR | 7/57,924 | Case-controls (5)  Cohorts (2) | PDs | HNC | (+) vs (-) | OR | PDs were defined as the presence of alveolar bone loss, tooth mobility, clinical attachment loss. | Clinical measurement and self-report. |
| Zeng, 2016 | 27294431 | China | PubMed, Scopus, and ScienceDirect from 2003 to 2014 | NR | 5/321,420 | Cohorts (5) | PDs | Lung cancer | (+) vs (-) | HR | PDs were defined as the presence of alveolar bone loss, tooth mobility, periodontitis, clinical attachment loss. | Clinical diagnosis and medical records. |
| Zhang, 2020 | 31697412 | China | WoS, PubMed, Cochrane Library, CNKI, CBM and Wanfang from 2003 to 2018 | NOS (7-9) | 9/455,183 | Cohorts (9) | Periodontitis | CRC | (+) vs (-) | HR | - | Clinical measurement and self-report. |
|  |  |  |  |  |  |  |  | Pancreatic cancer |  |  |  |  |
|  |  |  |  |  |  |  |  | Stomach cancer |  |  |  |  |
|  |  |  |  |  |  |  |  | Esophageal cancer |  |  |  |  |
|  |  |  |  |  |  |  |  | Liver cancer |  |  |  |  |
| Zhang, 2023 | 37689446 | China | Medline, Embase, PubMed, Cochrane Library and WoS from 2003 to 2022 | NOS (6-9) | 25/931,181 | Case-controls (11)  Cohorts (14) | PDs | Lung cancer | (+) vs (-) | OR/HR | Microbes were detected from subgingival plaque. | Clinical measurement, self-report, and medical records. |
| Espejo-Carrera, 2025 | 40192116 | Peru | PubMed, WoS, Scopus, Embase, and BVS from 2008 to 2022 | NOS (7-9) | 8/937,930 | Prospective (7)  Retrospective (1) | Periodontitis | CRC | (+) vs (-) | RR | Periodontitis was defined using clinical, radiographic  assessment, and epidemiological periodontal indicators. | Hospital records and self-report. |
| Wu, 2025 | 39928298 | China | PubMed, Embase, Cochrane Library, and WoS from 2003 to 2022 | NOS (6-8) | 7/157,934 | Prospective (5)  Case-control (1) | PDs | CRC | (+) vs (-) | OR | - | Hospital records, clinical measurement and self-report. |
| Chauca-Bajana, 2026 | 41744919 | Ecuador | Rayyan QCRI platform, PubMed, Embase, WoS, Cochrane Library, ClinicalTrials.gov, AIM, LILACS, IMEMR, IMSEAR, and WPRIM from 2007 to 2019 | NOS (6 studies were with concerns) | 6/12,825 | Case-control (4)  Retrospective (2) | Periodontitis | Oral and/or oropharyngeal cancer | (+) vs (-) | HR | Periodontitis (gingivitis and/or periodontitis), defined by clinical and/or  radiographic assessment (e.g., ABL, tooth mobility,and gingival recession) or by validated self-reported measures; classifications based on severity, extent, or duration of periodontitis were accepted. | Hospital records, clinical measurement and self-report. |
| Duan, 2026 | 41889038 | China | PubMed, and WoS from 2007 to 2024 | NOS (7-9) | 26/16,257,141 | Retrospective (26) | PDs | CRC | (+) vs (-) | RR | - | Clinical measurement and self-report. |
|  |  |  |  |  |  |  |  | Pancreatic cancer |  |  |  |  |
|  |  |  |  |  |  |  |  | Gastric cancer |  |  |  |  |
|  |  |  |  |  |  |  |  | Esophageal cancer |  |  |  |  |
|  |  |  |  |  |  |  |  | Liver cancer |  |  |  |  |
|  |  |  |  |  |  |  |  | Prostate cancer |  |  |  |  |
|  |  |  |  |  |  |  |  | Melanoma |  |  |  |  |
|  |  |  |  |  |  |  |  | Lung cancer |  |  |  |  |
|  |  |  |  |  |  |  |  | Kidney cancer |  |  |  |  |
|  |  |  |  |  |  |  |  | Hematologic cancer |  |  |  |  |
|  |  |  |  |  |  |  |  | HNC |  |  |  |  |
|  |  |  |  |  |  |  |  | Breast cancer |  |  |  |  |
|  |  |  |  |  |  |  |  | Bladder cancer |  |  |  |  |
| Li, 2026 | 41800498 | China | PubMed, Embase, CENTRAL, CNKI, and WanFang databases from 2003 to 2023 | NOS (6-9) | 16/1,212,459 | Retrospective cohorts (13)  Cross-sectional (2)  Case-control (1) | PDs | Prostate cancer | (+) vs (-) | HR | PDs were defined as the presence of periodontitis, or gingivitis. | Hospital records, clinical measurement and self-report. |
| Ridho, 2025 | <https://urologiacolombiana.com>/en/2025-en/periodontal-disease-and-prostate-cancer-a-systematic-review-and-meta-analysis/ | - | PubMed, Scopus, ScienceDirect, and Google Scholar from 2003 to 2023 | NOS (7-9) | 9/621,982 | Prospective cohorts (6)  Retrospective cohorts (3) | PDs | Prostate cancer | (+) vs (-) | OR | - | Hospital records, clinical measurement and self-report. |
| Cueva, 2026 | 42074956 | Ecuador | Rayyan QCRI platform, PubMed, Embase, WoS, Scopus, Cochrane Library, ClinicalTrials.gov and  the five regional WHO databases (AIM, LILACS, IMEMR, IMSEAR, WPRIM) from 2003 to 2022 | NOS (all were rated as low risk of bias) | 8/476,245 | Prospective cohorts (5)  Retrospective cohorts (2)  Case-control (1) | Periodontitis | Pancreatic cancer | (+) vs (-) | HR | Periodontitis: diagnosed clinically, radiographically, or through a history of tooth loss attributable to PDs | Hospital records, clinical measurement and self-report. |
| Manzaba, 2026 | 42074964 | Ecuador | Rayyan QCRI platform, PubMed, Embase, WoS, Scopus, Cochrane Library, ClinicalTrials.gov and  the five regional WHO databases (AIM, LILACS, IMEMR, IMSEAR, WPRIM) from 2007 to 2019 | NOS (1 was rated as low risk, 4 were rated as some concerns) | 5/2,098 | Case-control (5) | PDs | Oral squamous cell carcinoma | (+) vs (-) | HR | PDs were defined using clinical and/or radiographic criteria such as chronic or aggressive periodontitis, clinical attachment loss, probing depth, or alveolar bone loss | - |

**STable 4. Assessment of corrected covered area.**

**STable 4.1 Corrected covered area on periodontal diseases and gastric cancer**

| **Association: PDs and gastric cancer** | | | |
| --- | --- | --- | --- |
| **Meta-analysis** | Aguiar, 2024 | Wang, 2024 | Duan, 2026 |
| **Original study** |  |  |  |
| Watabe, 1998 | • |  |  |
| Hiraki, 2008 | • |  |  |
| Michaud, 2008 | • | • | • |
| Arora, 2010 | • | • |  |
| Shakeri, 2013 | • |  |  |
| Wen, 2013 | • |  |  |
| Chung, 2015 | • |  |  |
| Nwizu, 2017 | • | • | • |
| Chou, 2018 | • |  | • |
| Lee, 2023 |  | • |  |
| Kim, 2022 |  | • | • |
| Lo, 2021 |  | • | • |
| Lee, 2020 |  | • |  |
| Zhang, 2022 |  |  | • |
| Kang, 2023 |  |  | • |
| **Total** | **9** | **7** | **7** |
| **Grand total (N)** | **23** | | |
| **Rows (r)** | **15** | | |
| **Columns (c)** | **3** | | |
| **CCA** | **26.67%** | | |
| **Degree of overlap** | **Critically high** | | |

**STable 4.2 Corrected covered area on periodontal diseases and lung cancer**

| **Association: PDs and lung cancer** | | | | | | |
| --- | --- | --- | --- | --- | --- | --- |
| **Meta-analysis** | Zeng, 2016 | Chen, 2020 | Wang, 2022 | Zhang, 2023 | Verma, 2023 | Duan, 2026 |
| **Original study** |  |  |  |  |  |  |
| Guven, 2019 |  | • |  |  |  |  |
| Yoon, 2019 |  | • | • | • | • |  |
| Heikkila, 2018 |  | • |  |  |  |  |
| Michaud, 2018 |  | • | • | • | • |  |
| Nwizu, 2017 |  | • | • | • | • |  |
| Chrysanthakopoulos, 2016 |  | • | • | • | • |  |
| Arora, 2010 | • | • | • | • | • | • |
| Michaud, 2008 | • | • | • |  |  | • |
| Hujoel, 2003 | • | • | • |  |  |  |
| Mischaud, 2016 |  |  |  | • | • |  |
| Mai, 2016 |  |  | • | • | • | • |
| Wen, 2014 | • |  |  |  |  |  |
| Mai, 2014 | • |  |  |  |  | • |
| Kim, 2022 |  |  |  | • |  | • |
| Kim, 2024 |  |  |  |  |  | • |
| Kang, 2023 |  |  |  |  |  | • |
| **Total** | **5** | **9** | **8** | **8** | **7** | **7** |
| **Grand total (N)** | **44** | | | | | |
| **Rows (r)** | **16** | | | | | |
| **Columns (c)** | **6** | | | | | |
| **CCA** | **35.00%** | | | | | |
| **Degree of overlap** | **Critically high** | | | | | |

**STable 4.3 Corrected covered area on periodontitis and lung cancer**

| **Association: periodontitis and lung cancer** | | |
| --- | --- | --- |
| **Meta-analysis** | Wang, 2022 | Kesharani, 2022 |
| **Original study** |  |  |
| Michaud, 2008 | • | • |
| Mai, 2014 | • | • |
| Wen, 2014 | • | • |
| Chrysanthakopoulos, 2016 | • | • |
| Mai, 2016 | • |  |
| Michaud, 2016 | • | • |
| Dizdar, 2017 | • |  |
| Han, 2017 | • |  |
| Nwizu, 2017 | • | • |
| Michaud, 2018 | • | • |
| Guven, 2019 | • |  |
| Lu, 2019 | • |  |
| Tai, 2019 | • | • |
| Yoon, 2019 | • | • |
| Hujoel, 2003 |  | • |
| Hiraki, 2008 |  | • |
| Arora, 2009 |  | • |
| **Total** | **14** | **12** |
| **Grand total (N)** | **26** | |
| **Rows (r)** | **17** | |
| **Columns (c)** | **2** | |
| **CCA** | **52.94%** | |
| **Degree of overlap** | **Critically high** | |

**STable 4.4 Corrected covered area on periodontal diseases and colorectal cancer**

| **Association: PDs and CRC** | | | | | |
| --- | --- | --- | --- | --- | --- |
| **Meta-analysis** | Li, 2021 | Xuan, 2021 | Wang, 2024 | Wu, 2025 | Duan, 2026 |
| **Original study** |  |  |  |  |  |
| Arora, 2010 | • | • | • | • | • |
| Ahn, 2012 | • |  |  |  |  |
| Michaud, 2016 | • | • | • |  | • |
| Mai, 2016 | • |  | • | • | • |
| Heravi, 2017 | • | • | • | • | • |
| Michaud, 2018 | • | • |  | • | • |
| Hu, 2018 | • | • | • |  | • |
| Nwizu, 2017 |  | • | • | • | • |
| Michaud, 2008 |  | • | • |  | • |
| Lee, 2018 |  | • |  |  |  |
| Hwang, 2014 |  | • |  |  |  |
| Kim, 2019 |  | • |  |  |  |
| Mai, 2014 |  | • |  |  |  |
| Lee, 2023 |  |  | • |  |  |
| Fu, 2022 |  |  | • |  |  |
| Kim, 2022 |  |  | • |  | • |
| Lu, 2019 |  |  | • |  |  |
| Hujoel, 2003 |  |  |  | • |  |
| Janati, 2022 |  |  |  | • |  |
| Chou, 2018 |  |  |  |  | • |
| Kang, 2023 |  |  |  |  | • |
| **Total** | **7** | **11** | **11** | **7** | **11** |
| **Grand total (N)** | **47** | | | | |
| **Rows (r)** | **21** | | | | |
| **Columns (c)** | **5** | | | | |
| **CCA** | **30.95%** | | | | |
| **Degree of overlap** | **Critically high** | | | | |

| **Association: periodontitis and CRC** | | | |
| --- | --- | --- | --- |
| **Meta-analysis** | Zhang, 2020 | Wang, 2022 | Espejo-Carrera, 2025 |
| **Original study** |  |  |  |
| Michaud, 2008 | • | • | • |
| Arora, 2010 | • | • | • |
| Mai, 2016 |  | • | • |
| Michaud, 2016 |  | • | • |
| Han, 2017 |  | • |  |
| Momen-Heravi, 2017 | • | • | • |
| Nwizu, 2017 | • | • | • |
| Chou, 2018 | • | • |  |
| Hu, 2018 |  | • |  |
| Lee, 2018 |  | • |  |
| Michaud, 2018 | • | • | • |
| Guven, 2019 |  | • |  |
| Lu, 2019 |  | • |  |
| Kim, 2022 |  |  | • |
| **Total** | **6** | **13** | **8** |
| **Grand total (N)** | **27** | | |
| **Rows (r)** | **14** | | |
| **Columns (c)** | **3** | | |
| **CCA** | **46.43%** | | |
| **Degree of overlap** | **Critically high** | | |

**STable 4.5 Corrected covered area on periodontitis and colorectal cancer**

**STable 4.6 Corrected covered area on periodontal diseases and pancreatic cancer**

| **Association: PDs and pancreatic cancer** | | | |
| --- | --- | --- | --- |
| **Meta-analysis** | Maisonneuv, 2017 | Wang, 2024 | Duan, 2026 |
| **Original study** |  |  |  |
| Lee, 2023 |  | • |  |
| Kim, 2022 |  | • | • |
| Yu, 2022 |  | • |  |
| Lee, 2020 |  | • |  |
| Gerlovin, 2019 |  | • | • |
| Nwizu, 2017 |  | • | • |
| Chang, 2016 | • | • | • |
| Michaud, 2016 |  | • |  |
| Arora, 2010 | • | • | • |
| Michaud, 2008 |  | • | • |
| Hujoel, 2003 | • |  |  |
| Michaud, 2006 | • |  | • |
| Ahn, 2012 | • |  |  |
| Huang, 2016 | • |  |  |
| Chou, 2018 |  |  | • |
| Kang, 2023 |  |  |  |
| **Total** | **6** | **10** | **9** |
| **Grand total (N)** | **25** | | |
| **Rows (r)** | **16** | | |
| **Columns (c)** | **2** | | |
| **CCA** | **28.13%** | | |
| **Degree of overlap** | **Critically high** | | |

**STable 4.7 Corrected covered area on periodontitis and pancreatic cancer**

| **Association: periodontitis and pancreatic cancer** | | |
| --- | --- | --- |
| **Meta-analysis** | Zhang, 2020 | Cueva, 2026 |
| **Original study** |  |  |
| Michaud, 2008 | • | • |
| Arora, 2010 | • |  |
| Nwizu, 2017 | • |  |
| Chou, 2018 | • |  |
| Chang, 2016 |  | • |
| Yu, 2022 |  | • |
| Gerlovin, 2019 |  | • |
| Heikkila, 2018 |  | • |
| Michaud, 2013 |  | • |
| Fan, 2018 |  | • |
| Stolzenberg-Solomon, 2003 |  | • |
| **Total** | **4** | **8** |
| **Grand total (N)** | **12** | |
| **Rows (r)** | **11** | |
| **Columns (c)** | **2** | |
| **CCA** | **9.09%** | |
| **Degree of overlap** | **Moderate** | |

**STable 4.8 Corrected covered area on periodontal diseases and prostate cancer**

| **Association: PDs and prostate cancer** | | | | | | |
| --- | --- | --- | --- | --- | --- | --- |
| **Meta-analysis** | Wei, 2021 | Guo, 2021 | Li, 2022 | Ridho, 2025 | Duan, 2026 | Li, 2026 |
| **Original study** |  |  |  |  |  |  |
| Arora, 2010 | • | • | • | • | • | • |
| Chung, 2020 | • |  | • | • |  |  |
| Heikkila, 2018 | • |  | • | • |  |  |
| Hujoel, 2003 | • | • | • | • |  |  |
| Lee, 2017 | • | • |  | • | • |  |
| Michaud, 2016 | • | • | • | • | • | • |
| Michaud, 2018 | • | • | • | • | • | • |
| Dizdar, 2017 |  | • |  |  |  |  |
| Guven, 2019 |  | • |  |  |  |  |
| Hwang, 2014 |  | • |  |  |  |  |
| Wen, 2014 |  | • |  |  |  |  |
| Kim, 2020 |  |  | • |  |  | • |
| Chen, 2023 |  |  |  | • |  | • |
| Beger-Luedde, 2023 |  |  |  |  |  | • |
| Meurman, 2022 |  |  |  | • |  |  |
| **Total** | **7** | **9** | **7** | **9** | **4** | **6** |
| **Grand total (N)** | **42** | | | | | |
| **Rows (r)** | **15** | | | | | |
| **Columns (c)** | **6** | | | | | |
| **CCA** | **36.00%** | | | | | |
| **Degree of overlap** | **Critically high** | | | | | |

**STable 4.9 Corrected covered area on periodontitis and prostate cancer**

| **Association: periodontitis and prostate cancer** | | |
| --- | --- | --- |
| **Meta-analysis** | Ma,2020 | Wang, 2022 |
| **Original study** |  |  |
| Michaud, 2008 |  | • |
| Arora, 2010 | • | • |
| Michaud, 2016 | • | • |
| Dizdar, 2017 |  | • |
| Lee, 2017 | • | • |
| Michaud, 2018 | • | • |
| Guven, 2019 |  | • |
| **Total** | **4** | **7** |
| **Grand total (N)** | **11** | |
| **Rows (r)** | **7** | |
| **Columns (c)** | **2** | |
| **CCA** | **57.15%** | |
| **Degree of overlap** | **Critically high** | |

**STable 4.10 Corrected covered area on periodontal diseases and liver cancer**

| **Association: PDs and liver cancer** | | | |
| --- | --- | --- | --- |
| **Meta-analysis** | Al-Maweri, 2021 | Wang, 2024 | Duan, 2026 |
| **Original study** |  |  |  |
| Lee, 2023 |  | • |  |
| Kim, 2022 |  | • | • |
| Nwizu, 2017 | • | • | • |
| Kang, 2023 |  |  | • |
| Yang, 2017 | • |  |  |
| Thistle, 2018 | • |  |  |
| Ansai, 2013 | • |  |  |
| Jordao, 2019 | • |  |  |
| **Total** | **5** | **3** | **3** |
| **Grand total (N)** | **11** | | |
| **Rows (r)** | **8** | | |
| **Columns (c)** | **3** | | |
| **CCA** | **18.75%** | | |
| **Degree of overlap** | **Critically high** | | |

**STable 4.11 Corrected covered area on periodontal diseases and esophageal cancer**

| **Association: PDs and esophageal cancer** | | |
| --- | --- | --- |
| **Meta-analysis** | Wang, 2024 | Duan, 2026 |
| **Original study** |  |  |
| Zhang, 2022 | • |  |
| Kim, 2022 | • | • |
| Lo, 2021 | • |  |
| Lee, 2020 | • |  |
| Nwizu, 2017 | • | • |
| Lee, 2014 | • |  |
| Michaud, 2008 | • | • |
| Kang, 2023 |  | • |
| Arora, 2010 |  | • |
| Hannah Oh, 2020 |  | • |
| **Total** | **7** | **6** |
| **Grand total (N)** | **13** | |
| **Rows (r)** | **10** | |
| **Columns (c)** | **2** | |
| **CCA** | **30.00%** | |
| **Degree of overlap** | **Critically high** | |

**STable 4.12 Corrected covered area on periodontal diseases and bladder cancer**

| **Association: PDs and bladder cancer** | | | |
| --- | --- | --- | --- |
| **Meta-analysis** | Xie, 2018 | Li, 2022 | Duan, 2026 |
| **Original study** |  |  |  |
| Michaud, 2008 | **•** |  | **•** |
| Arora, 2010 | **•** | **•** | **•** |
| Wen, 2014 | **•** |  |  |
| Nwizu, 2017 | **•** | **•** | **•** |
| Michaud, 2016 |  | **•** |  |
| Kim, 2022 |  |  | **•** |
| Kang, 2023 |  |  | **•** |
| Hannah Oh, 2020 |  |  | **•** |
| **Total** | **4** | **3** | **6** |
| **Grand total (N)** | **13** | | |
| **Rows (r)** | **8** | | |
| **Columns (c)** | **3** | | |
| **CCA** | **31.25%** | | |
| **Degree of overlap** | **Critically high** | | |

**STable 4.13 Corrected covered area on periodontal diseases and breast cancer**

| **Association: PDs and breast cancer** | | |  |
| --- | --- | --- | --- |
| **Meta-analysis** | Shao, 2018 | Shi, 2018 | Duan, 2026 |
| **Original study** |  |  |  |
| Hujoel, 2003 | • | • |  |
| Arora, 2010 | • | • | • |
| Soder, 2011 | • | • |  |
| Chung, 2016 | • | • | • |
| Mai, 2016 | • |  | • |
| Dizdar, 2017 | • | • |  |
| Han, 2017 | • |  |  |
| Nwizu, 2017 | • | • | • |
| Sfreddo, 2017 | • |  |  |
| Heikkila, 2018 | • |  |  |
| Michaud, 2018 | • | • |  |
| Freudenheim, 2016 |  |  | • |
| Jia, 2020 |  |  | • |
| Farhat, 2021 |  |  | • |
| **Total** | **11** | **7** | **7** |
| **Grand total (N)** | **25** | | |
| **Rows (r)** | **14** | | |
| **Columns (c)** | **3** | | |
| **CCA** | **39.29%** | | |
| **Degree of overlap** | **Critically high** | | |

**STable 4.14 Corrected covered area on periodontal diseases and head and neck cancer**

| **Association: PDs and HNC** | | | | |
| --- | --- | --- | --- | --- |
| **Meta-analysis** | Zeng, 2013 | Gopinath, 2020 | Bai, 2023 | Duan, 2026 |
| **Original study** |  |  |  |  |
| Rosenquist, 2005 | • | • |  |  |
| Tezal, 2007 | • | • |  |  |
| Rezende, 2008 | • | • |  |  |
| Tezal, 2009 | • | • |  |  |
| Moergel, 2013 |  | • |  |  |
| Moraes, 2016 |  | • |  |  |
| Laprise, 2016 |  | • |  |  |
| Chung, 2016 |  | • |  | • |
| Shin, 2019 |  | • |  |  |
| Sharma, 2020 |  |  | • |  |
| Saira, 2019 |  |  | • |  |
| Bornigen, 2017 |  |  | • |  |
| Eliot, 2013 |  |  | • |  |
| Winn, 1991 |  |  | • |  |
| Tezal, 2005 | • |  |  |  |
| Guha, 2007 | • |  |  |  |
| Michaud, 2008 | • |  |  | • |
| Divaris, 2010 | • |  |  |  |
| Nwizu, 2017 |  |  |  | • |
| Kim, 2022 |  |  |  | • |
| Kang, 2023 |  |  |  | • |
| **Total** | **8** | **9** | **5** | **5** |
| **Grand total (N)** |  | **27** | | |
| **Rows (r)** |  | **21** | | |
| **Columns (c)** |  | **4** | | |
| **CCA** |  | **9.52%** | | |
| **Degree of overlap** |  | **Moderate** | | |

**STable 4.15 Corrected covered area on alveolar bone loss and head and neck cancer**

| **Association: alveolar bone loss and HNC** | | |
| --- | --- | --- |
| **Meta-analysis** | Gopinath, 2020 | Vu, 2021 |
| **Original study** |  |  |
| Tezal, 2007 | • | • |
| Tezal, 2009 | • | • |
| Moergel, 2013 | • | • |
| Moraes, 2016 |  | • |
| Shin, 2019 | • | • |
| Rosenquist, 2005 | • |  |
| **Total** | **5** | **5** |
| **Grand total (N)** | **10** | |
| **Rows (r)** | **6** | |
| **Columns (c)** | **2** | |
| **CCA** | **66.67%** | |
| **Degree of overlap** | **Critically high** | |

**STable 4.16 Corrected covered area on periodontal diseases and oral cancer**

| **Association: PDs and oral cancer** | | | | | | | |
| --- | --- | --- | --- | --- | --- | --- | --- |
| **Meta-analysis** | Yao, 2014 | Ye, 2016 | Bai, 2023 | Mahuili, 2023 | Li, 2023 | Ma, 2024 | Zambrano, 2026 |
| **Original study** |  |  |  |  |  |  |  |
| Mazul, 2016 |  |  |  | • |  |  |  |
| Shewale, 2021 |  |  |  | • |  |  |  |
| Farquhar, 2017 |  |  |  | • |  |  |  |
| LF Garrote, 2001 |  | • |  | • |  |  |  |
| Guha, 2007 |  | • |  | • |  |  |  |
| Balaram, 2002 |  |  |  | • |  |  |  |
| Laprise, 2016 |  |  |  | • | • | • | • |
| Gupta, 2017 |  |  |  | • |  |  |  |
| Chang, 2013 |  |  |  | • |  |  |  |
| Tezal, 2009 | • | • |  | • |  | • |  |
| Lissowska, 2003 |  |  |  | • |  |  |  |
| Moergel, 2013 |  |  |  | • |  | • | • |
| Eliot, 2013 |  |  | • | • | • |  |  |
| Tezal, 2007 | • | • |  | • |  | • | • |
| Marques, 2008 |  |  |  | • |  |  |  |
| Saira, 2019 |  |  |  | • | • |  |  |
| Talamini, 2000 |  | • |  | • |  |  |  |
| Shin, 2019 |  |  |  | • | • | • | • |
| Gyorgy, 2021 |  |  |  |  | • |  |  |
| Chen, 2021 |  |  |  |  |  | • |  |
| Rosenquist, 2005 | • | • |  |  |  | • |  |
| Moraes, 2016 |  |  |  |  |  | • | • |
| Rezende, 2008 | • |  |  |  |  |  |  |
| Davaris, 2010 | • | • |  |  |  |  |  |
| Zheng, 1990 |  | • |  |  |  |  |  |
| Marshall, 1992 |  | • |  |  |  |  |  |
| Bundgaard, 1995 |  | • |  |  |  |  |  |
| Ansai, 2013 |  | • |  |  |  |  |  |
| Sharma, 2020 |  |  | • |  |  |  |  |
| **Total** | **5** | **11** | **2** | **18** | **5** | **8** | **5** |
| **Grand total (N)** | **64** | | | | | | |
| **Rows (r)** | **29** | | | | | | |
| **Columns (c)** | **7** | | | | | | |
| **CCA** | **20.11%** | | | | | | |
| **Degree of overlap** | **Critically high** | | | | | | |

**STable 4.17 Corrected covered area on periodontal diseases and hematopoietic and lymphatic cancers**

| **Association: PDs and HLC** | | |
| --- | --- | --- |
| **Meta-analysis** | Wu, 2020 | Duan, 2026 |
| **Original study** |  |  |
| Kristinsson, 2010 | • |  |
| Chung, 2015 | • | • |
| Mai, 2016 | • | • |
| Bertrand, 2017 | • | • |
| Nwizu, 2017 | • |  |
| Michaud, 2018 | • |  |
| Kang, 2023 |  | • |
| **Total** | **5** | **4** |
| **Grand total (N)** | **9** | |
| **Rows (r)** | **6** | |
| **Columns (c)** | **2** | |
| **CCA** | **50.00%** | |
| **Degree of overlap** | **Critically high** | |

**STable 5. Recalculation and evaluation of the associations between periodontal diseases and cancer incidence.**

| **Exposure** | **Authors, publication year** | **N studies** | **Cases/Sample size** | **Random-effect model** | | | **I^2^ (%)** | **Largest study**  **ES (95% CI)** | **95% PI** | **Egger’s P value** | **10% Credibility ceiling** | **Excess significance** | | **Evidence grade** |
| --- | --- | --- | --- | --- | --- | --- | --- | --- | --- | --- | --- | --- | --- | --- |
|  |  |  |  | **ES** | **ES (95% CI)** | **P value** |  |  |  |  |  | **O/E** | **P value** |  |
| **Head and neck cancer** | | | | | | | | | | | | | | |
| **Periodontal diseases** | AOM | 18 | 12,524/156,693 | OR | 2.42 (1.85-3.17) | 1.392e-10 | 92 (90-94) | 1.14 (1.07-1.23) | (0.76-7.66) | 0.000 | 1.280 (1.125-1.457) | 16/4.7463 | 9.981e-08 | Highly suggestive |
|  | Gopinath, 2020 | 9 | 6,629/82,844 | OR | 3.17 (1.78-5.64) | 0.00008492 | 93 (90-95) | 1.14 (1.07-1.23) | (0.44-22.95) | 0.007 | 1.464 (1.048-2.045) | 8/1.8887 | 2.754e-05 | Suggestive |
|  | Bai, 2023 | 5 | 1,452/3,257 | OR | 2.20 (1.19-4.06) | 0.01162028 | 95 (91-96) | 1.19 (0.98-1.46) | (0.21-23.32) | 0.219 | 1.296 (1.036-1.620) | 4/4.9640 | 0.03549 | Weak |
|  | Zeng, 2013 | 7 | 4,942/71,722 | OR | 2.83 (1.83-4.38) | 2.837e-06 | 89 (81-92) | 1.33 (1.07-1.65) | (0.63-12.67) | 0.125 | 1.433 (1.083-1.896) | 7/4.9770 | 0.315 | Suggestive |
|  | Duan, 2026 | 5 | 1,453/1,069,853 | RR | 1.07 (0.94-1.23) | 0.29968942 | 42 (0-78) | 1.20 (1.09-1.33) | (0.75-1.54) | 0.503 | 1.015 (0.905-1.138) | 1/1.840491 | 0.6581 | No significance |
| **Alveolar bone loss** | Gopinath, 2020 | 5 | 773/1,755 | OR | 3.54 (2.47-5.07) | 6.641e-12 | 42 (0-77) | 4.36 (3.16-6.02) | (1.29-9.67) | 0.613 | 2.572 (1.219-5.427) | 4/4.9400 | 0.05858 | Weak |
|  | Vu, 2021 | 5 | 626/1,378 | OR | 3.87 (2.70-5.53) | 1.273e-13 | 39 (0-77) | 4.36 (3.16-6.02) | (1.46-10.28) | 0.657 | 3.478 (1.417-8.537) | 5/4.8452 | 1 | Weak |
| **Periodontitis** | Chauca-Bajana, 2026 | 6 | -/12,825 | HR | 2.14 (1.53-2.98) | 7.175e-06 | 75 (25-87) | 1.33 (1.07-1.65) | (0.76-6.05) | 0.042 | 1.590 (1.132-2.234) | 6/4.7887 | 0.6077 | Suggestive |
| **Oral cancer** | | | | | | | | | | | | | | |
| **Periodontal diseases** | Ma, 2024 | 8 | 3,017/7,518 | OR | 2.94 (2.13-4.07) | 6.763e-11 | 52 (0-76) | 4.36 (3.16-6.01) | (1.22-7.12) | 0.703 | 2.254 (1.415-3.591) | 7/8.7083 | 0.0325 | Highly suggestive |
|  | Li, 2023 | 5 | 1,994/2,889 | OR | 3.28 (1.87-5.74) | 0.00003264 | 87 (69-93) | 2.27 (1.64-3.13) | (0.41-26.19) | 0.730 | 2.215 (1.212-4.049) | 5/4.5977 | 1 | Suggestive |
|  | Mahuili, 2023 | 18 | 5,560/11,998 | OR | 2.50 (1.80-3.46) | 3.935e-08 | 91 (88-93) | 1.07 (1.00-1.13) | (0.62-10.09) | 0.000 | 1.357 (1.116-1.649) | 14/18.6488 | 2.02e-05 | Highly suggestive |
|  | Yao, 2014 | 5 | 1,191/3,183 | OR | 3.53 (1.52-8.23) | 0.00347164 | 93 (87-95) | 1.22 (0.94-1.58) | (0.15-82.36) | 0.374 | 1.311 (0.981-1.752) | 3/4.8662 | 0.3174 | Weak |
|  | Ye, 2016 | 11 | 1,321/5,317 | OR | 2.17 (1.45-3.25) | 0.00015166 | 92 (87-94) | 1.06 (1.01-1.13) | (0.51-9.20) | 0.008 | 1.186 (1.014-1.387) | 8/4.8662 | 0.07071 | Suggestive |
|  | Bai, 2023 | 2 | 713/1,480 | OR | 1.93 (1.11-3.34) | 0.01944039 | - | 1.46 (0.99-2.15) | - | - | 1.579 (0.923-2.701) | 1/1.9510 | 0.0484 | Weak |
|  | Manzaba, 2026 | 5 | 987/2,098 | HR | 3.18 (1.94-5.21) | 4.643e-06 | 59 (0-83) | 2.39 (1.50-3.80) | (0.68-14.79) | 0.107 | 2.422 (1.236-4.745) | 5/4.9939 | 1 | Suggestive |
| **Lung cancer** | | | | | | | | | | | | | | |
| **Periodontal diseases** | Zhang, 2023 | 8 | 5,040/931,181 | HR | 1.39 (1.17-1.64) | 0.00011813 | 55 (0-77) | 1.13 (1.01-1.26) | (0.91-2.11) | 0.085 | 1.212 (1.043-1.408) | 5/8.6438 | 0.0002633 | Suggestive |
|  | Chen, 2020 | 9 | 2,835/224,058 | RR | 1.37 (1.16-1.63) | 0.00028926 | 63 (0-80) | 1.31 (1.14-1.51) | (0.84-2.24) | 0.768 | 1.267 (1.049-1.529) | 6/6.0018 | 1 | Suggestive |
|  | Verma, 2023 | 7 | 23,811/108,056 | RR | 1.52 (1.26-1.85) | 0.00001706 | 82 (59-90) | 1.31 (1.19-1.44) | (0.83-2.80) | 0.548 | 1.370 (1.129-1.663) | 5/6.2506 | 0.1675 | Suggestive |
|  | Wang, 2020 | 8 | 2,454/167,256 | HR | 1.44 (1.28-1.61) | 6.346e-10 | 5 (0-58) | 1.31 (1.14-1.51) | (1.21-1.71) | 0.030 | 1.446 (1.162-1.799) | 5/7.522 | 0.009512 | Highly suggestive |
|  | Zeng, 2016 | 5 | 1,649/321,420 | HR | 1.25 (1.11-1.41) | 0.00024645 | 30 (0-74) | 1.08 (0.92-1.27) | (0.92-1.69) | 0.367 | 1.156 (1.012-1.321) | 3/5 | 2.2e-16 | Suggestive |
|  | Duan, 2026 | 7 | 6,137/1,132,860 | RR | 1.30 (0.95-1.77) | 0.10587134 | 95 (92-96) | 0.87 (0.79-0.95) | (0.46-3.65) | 0.474 | 1.084 (0.918-1.281) | 4/4.9460096 | 0.426 | No significance |
| **Periodontitis** | Wang, 2022 | 14 | 4,629/997,679 | RR | 1.31 (1.14-1.51) | 0.00010928 | 63 (23-78) | 1.08 (0.92-1,27) | (0.87-1.99) | 0.426 | 1.165 (1.027-1.322) | 8/7.4490 | 0.7965 | Suggestive |
|  | Kesharani, 2022 | 12 | 5,037/410,510 | RR | 1.72 (1.41-2.10) | 7.261e-08 | 90 (85-93) | 1.23 (1.09-1.38) | (0.83-3.59) | 0.640 | 1.311 (1.144-1.542) | 9/11.0549 | 0.06274 | Highly suggestive |
| **Breast cancer** | | | | | | | | | | | | | | |
| **Periodontal diseases** | Duan, 2026 | 7 | 13,308/374,483 | RR | 1.10 (1.03-1.17) | 0.00261528 | 50 (0-77) | 1.02 (0.94-1.10) | (0.93-1.30) | 0.600 | 1.043 (0.991-1.098) | 3/0.52854853 | 0.01195 | Weak |
|  | Shao, 2018 | 11 | 3,953/173,162 | RR | 1.22 (1.06-1.40) | 0.00426437 | 51 (0-74) | 1.13 (1.03-1.23) | (0.88-1.70) | 0.095 | 1.140 (1.015-1.279) | 4/3.5138 | 0.7525 | Weak |
|  | Shi, 2018 | 7 | 3,471/166,283 | RR | 1.20 (1.10-1.31) | 0.00007648 | 18 (0-65) | 1.13 (1.03-1.23) | (1.01-1.43) | 0.080 | 1.190 (1.037-1.365) | 3/2.4982 | 0.7057 | Suggestive |
| **Periodontitis** | Wang, 2022 | 11 | >1,000/242,510 | HR | 1.26 (1.11-1.43) | 0.00042415 | 76 (51-85) | 1.13 (1.04-1.23) | (0.86-1.84) | 0.325 | 1.107 (1.018-1.203) | 6/9.8516 | 0.003332 | Suggestive |
| **Esophageal cancer** | | | | | | | | | | | | | | |
| **Periodontal diseases** | Wang, 2024 | 7 | NA/6,181,524 | HR | 1.39 (1.15-1.68) | 0.00055922 | 60 (0-81) | 1.36 (1.21-1.52) | (0.82-2.37) | 0.687 | 1.198 (1.002-1,432) | 4/7 | 2.2e-16 | Suggestive |
|  | Duan, 2026 | 6 | 2,637/1,092,434 | RR | 1.21 (1.09-1.35) | 0.00055026 | 26 (0, 70) | 1.04 (0.87-1.24) | (0.95-1.55) | 0.838 | 1.121 (0.993-1.265) | 2/0.44309333 | 0.06698 | Suggestive |
| **Periodontitis** | Ma, 2020 | 4 | NA/845,258 | HR | 1.79 (1.15-2.79) | 0.00939925 | 60 (0-85) | 1.27 (0.89-1.82) | (0.32-10.06) | 0.118 | 1.383 (1.026-1.863) | 2/4 | 2.2e-16 | Weak |
| **Gastric cancer** | | | | | | | | | | | | | | |
| **Periodontal diseases** | Aguiar, 2024 | 9 | 7,489/382,951 | RR | 1.17 (1.03-1.33) | 0.01742115 | 42 (0-73) | 1.23 (1.18-1.29) | (0.86-1.59) | 0.586 | 1.073 (0.948-1.215) | 2/3.0817 | 0.7273 | Weak |
|  | Wang, 2024 | 7 | 12,657/4,986,976 | HR | 1.13 (1.01-1.26) | 0.0324435 | 75 (34-87) | 0.99 (0.96-1.02) | (0.83-1.53) | 0.057 | 1.054 (0.968-1.148) | 2/7 | 2.2e-16 | Weak |
|  | Duan, 2026 | 7 | 1,669/6,243,547 | RR | 1.17 (1.00, 1.37) | 0.04308769 | 44 (0-75) | 1.04 (0.86, 1.26) | (0.79-1.75) | 0.415 | 1.068 (0.943-1.210) | 2/0.44094277 | 0.0674 | Weak |
| **Periodontitis** | Zhang, 2020 | 4 | 463/197,249 | HR | 1.12 (0.88-1.42) | 0.36717454 | 0 (0-68) | 1.01 (0.68-1.49) | (0.66-1.89) | 0.980 | 1.077 (0.838-1.384) | 0/4 | 2.2e-16 | No significance |
| **Pancreatic cancer** | | | | | | | | | | | | | | |
| **Periodontal diseases** | Wang, 2024 | 10 | NA/10,296,531 | HR | 1.35 (1.00-1.82) | 0.04832781 | 97 (97-98) | 2.00 (1.90-2.11) | (0.46-3.99) | 0.584 | 1.012 (0.922-1.110) | 4/10 | 2.2e-16 | Weak |
|  | Maisonneuv, 2017 | 6 | 653/322,455 | RR | 1.74 (1.41-2.15) | 3.206e-07 | 0 (0-61) | 1.64 (1.19-2.26) | (1.29-2.34) | 0.020 | 1.771 (1.216-2.580) | 3/4.5416 | 0.1586 | Weak |
|  | Duan, 2026 | 9 | 1,853/1,398,153 | RR | 1.25 (1.03-1.50) | 0.02225104 | 62 (0-80) | 1.04 (0.85-1.27) | (0.72-2.15) | 0.215 | 1.050 (0.931-1.185) | 4/0.55067141 | 0.0674 | Weak |
| **Periodontitis** | AOM | 11 | -/1,030,433 | HR | 1.44 (1.21-1.72) | 0.00005429 | 60 (2-78) | 1.20 (1.11-1.29) | (0.87-2.38) | 0.066 | 1.209 (1.011-1.444) | 8/10.9343 | 3.392e-05 | Suggestive |
|  | Cueva, 2026 | 8 | 7,845/881,559 | HR | 1.56 (1.29-1.89) | 5.810e-06 | 55 (0-78) | 1.20 (1.11-1.29) | (0.94-2.59) | 0.001 | 1.391 (1.128-1.715) | 7/7.9343 | 0.06384 | Suggestive |
|  | Zhang, 2020 | 4 | NA/197,249 | HR | 1.25 (0.84-1.85) | 0.26779335 | 73 (0-88) | 1.54 (1.16-2.04) | (0.24-6.63) | 0.826 | 1.060 (0.764-1.470) | 2/4 | 2.2e-16 | No significance |
| **Liver cancer** | | | | | | | | | | | | | | |
| **Periodontal diseases** | Duan, 2026 | 3 | 3,449/979,240 | RR | 1.12 (1.04-1.22) | 0.00372495 | 0 (0-73) | 1.12 (1.01-1.25) | (0.67-1.88) | 0.070 | 1.129 (1.002-1.273) | 1/1.5120062 | 0.622 | Weak |
|  | Wang, 2024 | 3 | NA/3,918,799 | HR | 1.09 (1.04-1.13) | 0.00020667 | 0 (0-73) | 1.08 (1.03-1.13) | (0.82-1.44) | 0.040 | 1.097 (0.999-1.204) | 1/2.9648 | 0.0004098 | Weak |
| **Colorectal cancer** | | | | | | | | | | | | | | |
| **Periodontal diseases** | Wang, 2024 | 11 | 126,733/5,042,335 | HR | 1.21 (1.05-1.39) | 0.00721114 | 89 (83-92) | 1.07 (1.04-1.11) | (0.76-1.91) | 0.316 | 1.066 (0.998-1.139) | 5/10.9364 | 1.684e-11 | Weak |
|  | Li, 2021 | 7 | 3,884/337894 | HR | 1.44 (1.18-1.76) | 0.00033293 | 54 (0-78) | 1.64 (1.50-1.80) | (0.86-2.42) | 0.662 | 1.231 (1.019-1.486) | 3/7 | 2.2e-16 | Suggestive |
|  | Xuan, 2021 | 11 | 122,363/610,811 | RR | 1.21 (1.06-1.38) | 0.00404947 | 84 (72-89) | 1.25 (1.15-1.36) | (0.77-1.90) | 0.342 | 1.052 (0.963-1.150) | 5/7.3997 | 0.1943 | Weak |
|  | Wu, 2025 | 7 | 24,348/157,934 | OR | 1.42 (1.04-1.94) | 0.02611782 | 74 (30-86) | 1.06 (0.89-1.27) | (0.57, 3.57) | 0.331 | 1.115 (0.980, 1.270) | 1/5.9873 | 5.622e-05 | Weak |
|  | Duan, 2026 | 11 | 11,034/1,283,990 | RR | 1.24 (1.05-1.47) | 0.01046061 | 88 (81-92) | 1.04 (0.96-1.13) | (0.71, 2.16) | 0.698 | 1.047 (0.985-1.113) | 5/1.1614082 | 0.003503 | Weak |
| **Periodontitis** | Wang, 2022 | 13 | NA/652,568 | HR | 1.18 (1.03-1.35) | 0.01393999 | 79 (63-86) | 1.25 (1.17-1.34) | (0.76-1.85) | 0.381 | 1.051 (0.967-1.141) | 4/13.8568 | 2.2e-16 | Weak |
|  | Zhang, 2020 | 6 | NA/992,480 | HR | 1.12 (0.96-1.30) | 0.15604977 | 56 (0-80) | 1.05 (0.90-1.23) | (0.72-1.73) | 0.027 | 1.034 (0.938-1.140) | 2/6 | 2.2e-16 | No significance |
|  | Espejo-Carrera, 2025 | 8 | 6,605/937,930 | RR | 1.34 (0.96-1.89) | 0.08815446 | 95 (93-96) | 2.38 (2.17-2.60) | (0.42, 4.34) | 0.202 | 1.079 (0.949-1.227) | 2/5.7431 | 0.008131 | No significance |
| **Bladder cancer** | | | | | | | | | | | | | | |
| **Periodontal diseas**es | Li, 2022 | 3 | 712/101,135 | HR | 1.19 (0.95-1.49) | 0.13443534 | 0 (0-73) | 1.10 (0.81-1.49) | (0.27-5.16) | 0.873 | 1.166 (0.916-1.483) | 0/3 | 2.2e-16 | No significance |
|  | Xie, 2018 | 4 | 1,094/298,476 | HR | 1.09 (0.95-1.26) | 0.22697859 | 0 (0-68) | 1.17 (0.96-1.43) | (0.80-1.48) | 0.736 | 1.077 (0.924-1.255) | 0/4 | 2.2e-16 | No significance |
|  | Duan, 2026 | 6 | 2,637/1,092,434 | RR | 1.21 (1.09-1.35) | 0.00055026 | 26 (0-70) | 1.04 (0.87-1.24) | (0.95-1.55) | 0.838 | 1.121 (0.993-1.265) | 2/0.44309333 | 0.003503 | Suggestive |
| **Kidney cancer** | | | | | | | | | | | | | | |
| **Periodontal diseas**es | Duan, 2026 | 4 | 1,197/1,027,615 | RR | 1.16 (0.93-1.44) | 0.19312021 | 65 (0-86) | 0.92 (0.76-1.12) | (0.46-2.89) | 0.452 | 1.068 (0.884-1.290) | 1/0.50265817 | 0.4156 | No significance |
| **Prostate cancer** | | | | | | | | | | | | | | |
| **Periodontal diseases** | Li, 2026 | 6 | -/882,540 | HR | 1.21 (1.09-1.34) | 0.00032701 | 48 (0-75) | 1.24 (1.16-1.32) | (0.94-1.55) | 0.965 | 1.140 (1.007-1.290) | 4/8 | < 2.2e-16 | Suggestive |
|  | Wei, 2021 | 7 | 3,552/294,399 | RR | 1.17 (1.06-1.29) | 0.00111914 | 6 (0-61) | 1.14 (1.01-1.31) | (1.01-1.36) | 0.078 | 1.140 (1.017-1.278) | 3/2.4373 | 0.7005 | Weak |
|  | Guo, 2021 | 9 | 3,353/440,911 | HR | 1.40 (1.16-1.70) | 0.00055068 | 76 (47-86) | 1.14 (1.01-1.31) | (0.77-2.57) | 0.088 | 1.149 (1.029-1.284) | 5/9 | 2.2e-16 | Suggestive |
|  | Li, 2022 | 7 | 6,240/273,305 | HR | 1.24 (1.17-1.31) | 5.310e-14 | 0 (0-58) | 1.24 (1.16-1.32) | (1.15-1.34) | 0.728 | 1.211 (1.053-1.394) | 4/7 | 2.2e-16 | Highly Suggestive |
|  | Duan, 2026 | 4 | 1,869/275,876 | RR | 1.17 (1.07-1.29) | 0.00084002 | 0 (0-68) | 1.14 (1.00-1.30) | (0.95-1.44) | 0.116 | 1.170 (1.031-1.328) | 1/1.3436628 | 1 | Suggestive |
|  | Ridho, 2025 | 9 | -/621,982 | HR | 1.20 (1.12-1.28) | 2.317e-07 | 1 (0, 55) | 1.22 (1.10-1.35) | (1.10-1.30) | 0.110 | 1.159 (1.042-1.289) | 3/8.8913 | 2.527e-10 | Highly Suggestive |
| **Periodontitis** | Wang, 2022 | 7 | -/97,420 | HR | 1.26 (1.03-1.54) | 0.02287093 | 73 (24-86) | 1.14 (0.99-1.31) | (0.68-2.32) | 0.144 | 1.100 (0.956-1.266) | 3/6.4315 | 0.001245 | Weak |
|  | Ma, 2020 | 4 | -/229,256 | HR | 1.20 (1.09-1.32) | 0.0002239 | 0 (0-68) | 1.15 (1.01-1.31) | (0.97-1.47) | 0.095 | 1.193 (1.035-1.375) | 3/4 | 2.2e-16 | Suggestive |
| **Melanoma** | | | | | | | | | | | | | | |
| **Periodontal diseases** | Duan, 2026 | 2 | 711/49,712 | RR | 1.06 (0.86-1.30) | 0.60096343 | - | 1.06 (0.86-1.30) | - | - | 1.056 (0.861-1.296) | 0/0.18634197 | 1 | No significance |
| **Hematopoietic and lymphatic cancers** | | | | | | | | | | | | | | |
| **Periodontal diseases** | Duan, 2026 | 4 | 1,536/295,274 | RR | 1.33 (0.82-2.13) | 0.24527011 | 96 (94-97) | 0.98 (0.87-1.10) | (0.15-11.45) | 0.940 | 1.056 (0.852-1.310) | 2/0.22180356 | 0.01711 | No significance |
|  | Wu, 2020 | 6 | 2,851/214,004 | HR | 1.06 (0.93-1.22) | 0.38070122 | 40 (0-69) | 1.11 (0.95-1.30) | (0.77-1.48) | 0.060 | 1.057 (0.936-1.193) | 2/ 0.8827 | 0.2191 | No significance |
|  |  |  |  |  |  |  |  |  |  |  |  |  |  |  |

**Abbreviation:** OR, odds ratio; RR, risk ratio; HR, hazard ratio; N, included original studies; ES, estimate size; CI, confidence interval; PI, predictive interval.

**STable 6. Subgroup analysis of periodontal diseases and cancer stratified by study design.**

| **Exposure** | **Authors, publication year** | **Study design** | **N studies/total studies** | **Cases/Sample size** | **Random-effect model** | | | **I^2^ (%)** | **Largest study**  **ES (95% CI)** | **95% PI** | **Egger’s P value** | **10% Credibility ceiling** | **Excess significance** | | **Evidence grade** |
| --- | --- | --- | --- | --- | --- | --- | --- | --- | --- | --- | --- | --- | --- | --- | --- |
|  |  |  |  |  | **ES** | **ES (95% CI)** | **P value** |  |  |  |  |  | **O/E** | **P value** |  |
| **Head and neck cancer** | | | | | | | | | | | | | | | |
| **Periodontal diseases** | Zeng, 2013 | Cohort | 2 | 249/62,304 | OR | 2.23 (0.58, 8.61) | 0.24479103 | - | 1.15 (0.73, 1.81) | - | - | 1.404 (0.542, 3.638) | 1/1.9874 | - | Not significance |
|  |  | Case-control | 7 | 4,760/57,688 | OR | 2.45 (1.52, 3.94) | 0.00022478 | 90 (81, 93) | 1.33 (1.07, 1.65) | (0.49, 12.13) | 0.337 | 1.385 (1.043, 1.840) | 5/6.884 | 0.005456 | Suggestive |
| **Lung cancer** | | | | | | | | | | | | | | | |
| **Periodontal diseases** | Zhang, 2023 | Case-control | 2 | 467/2,215 | OR | 1.68 (1.11, 2.56) | 0.01502385 | - | 1.53 (1.20, 1.94) | - | - | 1.671 (0.918, 3.040) | 2/1.5453 | 1 | Weak |
|  |  | Cohort | 6 | 4,573/928,966 | HR | 1.32 (1.10, 1.58) | 0.00240305 | 53 (0, 78) | 1.13 (1.01, 1.26) | (0.85, 2.04) | 0.235 | 1.186 (1.016, 1.385) | 3/4.703 | 0.2272 | Weak |
|  | Chen, 2020 | Cohort | 6 | 1,690/173,468 | RR | 1.30 (1.00, 1.69)) | 0.05179339 | 72 (5, 86) | 1.31 (1.14, 1.51) | (0.57, 2.97) | 0.960 | 1.208 (0.947, 1.540) | 3/2.9146 | 1 | Not significance |
|  |  | Case-control | 2 | 467/2,215 | RR | 1.67 (0.90, 2.83) | 0.05647603 | - | 1.44 (1.09, 1.91) | - | - | 1.551 (0.919, 2.620) | 2/1.5357 | 1 | Not significance |
|  | Zeng, 2016 | Prospective cohort | 4 | 1,406/167,854 | HR | 1.32 (1.18, 1.48) | 7.979e-07 | 0 (0, 68) | 1.25 (1.06, 1.47) | (1.04, 1.69) | 0.293 | 1.340 (1.057, 1.699) | 3/2.5687 | 1 | Convincing |
|  | Wang, 2020 | Cohort | 6 | 1,987/165,041 | HR | 1.43 (1.25, 1.64) | 2.369e-07 | 9 (0, 65) | 1.31 (1.14, 1.51) | (1.11, 1.84) | 0.136 | 1.424 (1.120, 1.812) | 3/4.3295 | 0.358 | Convincing |
|  |  | Case-control | 2 | 467/2,215 | HR | 1.67 (0.99, 2.83) | 0.05647603 | - | 1.44 (1.09, 1.91) | - | - | 1.551 (0.919, 2.620) | 2/1.5357 | 1 | Not significance |
|  | Verma, 2023 | Cohort | 5 | 3,107/22,254 | RR | 1.64 (1.26, 2.14) | 0.00024358 | 82 (49, 91) | 1.35 (1.14, 1.60) | (0.65, 4.15) | 0.967 | 1.458 (1.109, 1.918) | 4/4.3315 | 0.5121 | Suggestive |
|  |  | Case-control | 2 | 20,704/85,802 | RR | 1.31 (1.19, 1.43) | 1.109e-08 | - | 1.31 (1.19, 1.44) | - | - | 1.287 (0.979, 1.693) | 1/2 | 2.2e-16 | Highly suggestive |
| **Periodontitis** | Kesharaini, 2022 | Cohort | 8 | 3,074/257,483 | RR | 2.07 (1.67, 2.56) | 2.113e-11 | 82 (63, 89) | 1.83 (1.60, 2.09) | (1.05, 4.09) | 0.982 | 1.460 (1.078, 1.978) | 6/7.8159 | 0.01352 | Highly suggestive |
|  |  | Case-control | 4 | 1,963/153,027 | RR | 1.27 (1.17, 1.38) | 1.386e-08 | 0 (0, 68) | 1.23 (1.09, 1.38) | (1.06, 1.53) | 0.837 | 1.256 (1.036, 1.521) | 3/2.5983 | 1 | Convincing |
|  | Wang, 2022 | Prospective | 11 | 4,154/889,734 | RR | 1.31 (1.11, 1.55) | 0.00110048 | 68 (29, 81) | 1.31 (1.14, 1.51) | (0.82, 2.11) | 0.323 | 1.156 (1.002, 1.333) | 6/6.151 | 1 | Weak |
|  |  | Retrospective | 3 | 465/107,945 | RR | 1.35 (1.02, 1.78) | 0.03558636 | 34 (0, 81) | 1.33 (1.08, 1.64) | (0.10, 18.49) | 0.605 | 1.260 (0.831, 1.912) | 2/1.1595 | 0.5636 | Weak |
| **Breast cancer** | | | | | | | | | | | | | | | |
| **Periodontal diseases** | Shao, 2018 | Prospective cohort | 8 | 3,514/130,262 | RR | 1.17 (0.97, 1.41) | 0.09481993 | 48 (0, 75) | 1.13 (1.03, 1.23) | (0.73, 1.87) | 0.222 | 1.110 (0.980, 1.258) | 2/2.103 | 1 | Not significance |
|  |  | Retrospective cohort | 2 | 372/42,699 | RR | 1.41 (0.83, 2.41) | 0.20201845 | - | 1.23 (1.11, 1.36) | - | - | 1.274 (0.936, 1.734) | 1/0.9659 | 1 | Not significance |
| **Periodontitis** | Wang, 2022 | Prospective cohort | 9 | -/219,361 | HR | 1.22 (1.07, 1.40) | 0.00393455 | 79 (55, 87) | 1.13 (1.04, 1.23) | (0.81, 1.83) | 0.541 | 1.094 (1.005, 1.191) | 5/8.3366 | 0.002749 | Weak |
|  |  | Retrospective cohort | 2 | -/23,149 | HR | 1.52 (1.17, 1.98) | 0.00171938 | - | 1.55 (1.14, 2.11) | - | - | 1.491 (0.967, 2.300) | 1/1.8433 | 0.1506 | Weak |
| **Esophagus cancer** | | | | | | | | | | | | | | | |
| **Periodontitis** | Ma, 2020 | Prospective cohort | 3 | -/126,849 | HR | 2.28 (1.16, 4.48) | 0.01678927 | 63 (0, 87) | 1.44 (0.98, 2.11) | (0, 3498.75) | 0.331 | 1.637 (0.978, 2.742) | 2/3 | 2.2e-16 | Weak |
| **Gastric cancer** | | | | | | | | | | | | | | | |
| **Periodontal diseases** | Aguiar, 2024 | Cohort | 6 | 1,698/365,583 | RR | 1.12 (0.95, 1.31) | 0.17001643 | 40 (0, 75) | 1.23 (1.18, 1.29) | (0.75, 1.66) | 0.262 | 1.041 (0.891, 1.217) | 1/0.918 | 1 | Not significance |
|  |  | Case-control | 3 | 5,791/17,368 | RR | 1.34 (0.94, 1.92) | 0.10933528 | 62 (0, 87) | 1.08 (0.87, 1.35) | (0.03, 64.02) | 0.652 | 1.130 (0.920, 1.388) | 1/2.3541 | 0.1191 | Not significance |
| **Colorectal cancer** | | | | | | | | | | | | | | | |
| **Periodontal diseases** | Xuan, 2021 | Cohort | 9 | 117,011/565,436 | RR | 1.18 (0.98, 1.43) | 0.08132084 | 87 (76, 91) | 1.64 (1.50, 1.80) | (0.63, 2.23) | 0.276 | 1.028 (0.940, 1.124) | 3/4.7486 | 0.3228 | Not significance |
|  |  | Case-control | 2 | 5,352/45,375 | RR | 1.31 (1.17, 1.47) | 4.195e-06 | - | 1.25 (1.15, 1.36) | - | - | 1.295 (0.973, 1.725) | 2/1.5097 | 1 | Highly suggestive |
|  | Wu, 2025 | Prospective cohort | 6 | 24,305/132,928 | OR | 1.39 (0.99, 1.95) | 0.05808313 | 78 (36, 88) | 1.06 (0.89, 1.27) | (0.49, 3.92) | 0.449 | 1.103 (0.968, 1.258) | 1/5.6922 | 2.041e-06 | Not significance |
| **Periodontitis** | Wang, 2022 | Prospective cohort | 10 | -/519,077 | HR | 1.22 (1.01, 1.48) | 0.03796073 | 80 (62, 87) | 1.64 (1.49, 1.80) | (0.66, 2.26) | 0.338 | 1.064 (0.967, 1.171) | 3/10.9531 | 2.2e-16 | Weak |
|  |  | Retrospective cohort | 3 | -/133,491 | HR | 1.09 (0.87, 1.37) | 0.46738216 | 73 (0, 90) | 1.25 (1.17, 1.34) | (0.08, 14.04) | 0.389 | 1.013 (0.860, 1.192) | 1/3 | 2.2e-16 | Not significance |
|  | Espejo-Carrera, 2025 | Prospective | 7 | 3,189/224,729 | RR | 1.16 (0.99, 1.35) | 0.05993933 | 53 (0, 78) | 1.34 (1.15, 1.55) | (0.77, 1.74) | 0.569 | 1.063 (0.942, 1.200) | 1/2.3509 | 0.4359 | Not significance |
| **Bladder cancer** | | | | | | | | | | | | | | | |
| **Periodontal diseases** | Xie, 2018 | Prospective cohort | 3 | 906/144,910 | HR | 1.15 (0.98, 1.35) | 0.09516983 | 0 (0, 73) | 1.17 (0.96, 1.43) | (0.40, 3.27) | 0.632 | 1.142 (0.953, 1.368) | 0/0.6785 | 1 | Not significance |
| **Prostate cancer** | | | | | | | | | | | | | | | |
| **Periodontal diseases** | Wei, 2021 | Prospective cohort | 4 | 1,742/34,738 | RR | 1.18 (0.98, 1.42) | 0.08010446 | 35 (0, 78) | 1.01 (0.82, 1.23) | (0.63, 2.20) | 0.200 | 1.111 (0.958, 1.288) | 1/1.5781 | 1 | Not significance |
|  |  | Retrospective cohort | 3 | 1,810/259,661 | RR | 1.18 (1.05, 1.33) | 0.00523032 | 0 (0, 73) | 1.14 (1.01, 1.31) | (0.55, 2.51) | 0.275 | 1.186 (0.990, 1.420) | 2/1.4521 | 0.6134 | Weak |
|  | Guo, 2021 | Prospective cohort | 4 | 1,742/54,060 | HR | 1.27 (1.09, 1.48) | 0.00255133 | 0 (0, 68) | 1.17 (0.94, 1.47) | (0.90, 1.77) | 0.136 | 1.240 (1.034, 1.489) | 1/2.5207 | 0.1462 | Weak |
|  |  | Retrospective cohort | 5 | 1,611/386,851 | HR | 1.51 (1.09, 2.09) | 0.01300463 | 87 (70, 93) | 1.14 (1.01, 1.31) | (0.48, 4.79) | 0.214 | 1.119 (0.934, 1.339) | 4/3.1942 | 0.6599 | Weak |
|  | Li, 2022 | Prospective cohort | 6 | 5,390/193,025 | HR | 1.24 (1.17, 1.31) | 4.308e-13 | 0 (0, 61) | 1.24 (1.16, 1.32) | (1.14, 1.34) | 0.891 | 1.198 (1.033, 1.389) | 3/3.3308 | 1 | Convincing |
| **Hematopoietic and lymphatic cancers** | | | | | | | | | | | | | | | |
| **Periodontal diseases** | Wu, 2020 | Cohort | 5 | 2,204/210,817 | HR | 1.07 (0.93, 1.23) | 0.34024766 | 43 (0, 71) | 1.18 (1.02, 1.37) | (0.76, 1.50) | 0.100 | 1.059 (0.932, 1.202) | 2/0.685 | 0.1465 | Not significance |

**Abbreviation:** OR, odds ratio; RR, risk ratio; HR, hazard ratio; N, included original studies; ES, estimate size; CI, confidence interval; PI, predictive interval.

**STable 7. Subgroup analysis of periodontal diseases and cancer stratified by region.**

| **Exposure** | **Authors, publication year** | **Region** | **N studies** | **Cases/Sample size** | **Random-effect model** | | | **I^2^ (%)** | **Largest study**  **ES (95% CI)** | **95% PI** | **Egger’s P value** | **10% Credibility ceiling** | **Excess significance** | | **Evidence grade** |
| --- | --- | --- | --- | --- | --- | --- | --- | --- | --- | --- | --- | --- | --- | --- | --- |
|  |  |  |  |  | **ES** | **ES (95% CI)** | **P value** |  |  |  |  |  | **O/E** | **P value** |  |
| **Head and neck cancer** | | | | | | | | | | | | | | | |
| **Periodontal diseases** | Zeng, 2013 | America | 4 | 1,804/63,741 | OR | 2.29 (1.11, 4.76) | 0.0256776 | 94 (87, 96) | 1.33 (1.07, 1.65) | (0.07, 71.74) | 0.573 | 1.300 (0.954, 1.770) | 3/3.9918 | 0.008175 | Weak |
|  |  | Latin-America | 2 | 2,163/4,018 | OR | 4.68 (0.75, 29.33) | 0.09937884 | - | 1.94 (1.49, 2.53) | - | - | 2.187 (0.820, 5.830) | 2/1.9998 | 1 | Not significance |
|  |  | Europe | 2 | 924/2,172 | OR | 2.67 (1.67, 4.27) | 0.00003913 | - | 2.89 (1.74, 4.80) | - | - | 2.061 (0.776, 5.474) | 1/1.9999 | 8e-05 | Weak |
|  | Bai, 2023 | America | 3 | 976/2,306 | OR | 1.31 (1.10, 1.56) | 0.00206783 | 16 (0, 77) | 1.19 (0.98, 1.46) | (0.33, 5.21) | 0.190 | 1.262 (1.006, 1.583) | 2/1.8857 | 1 | Weak |
|  |  | Asia | 2 | 476/951 | OR | 4.31 (1.56, 11.90) | 0.00484342 | - | 7.22 (4.92, 10.59) | - | - | 3.099 (0.846, 11.353) | 2/2 | TRUE | Weak |
|  | Duan, 2026 | Asia | 3 | 1,315/955,609 | RR | 1.05 (0.88, 1.26) | 0.57051211 | 71 (0, 89) | 1.20 (1.09, 1.33) | (0.14, 7.75) | 0.432 | 1.003 (0.885, 1.137) | 1/1.4762614 | 1 | Not significance |
|  |  | America | 2 | 138/114,244 | RR | 1.13 (0.80, 1.60) | 0.49347357 | - | 1.15 (0.73, 1.81) | - | - | 1.129 (0.798, 1.598) | 0/0.19722307 | 1 | Not significance |
| **Alveolar bone loss** | Vu, 2021 | America | 2 | 317/578 | OR | 4.51 (3.37, 6.03) | 4.358e-24 | - | 4.36 (3.16, 6.02) | - | - | 4.725 (0.879, 25.408) | 2/1.9997 | 1 | Weak |
|  | Gopinath, 2020 | America | 2 | 317/578 | OR | 4.51 (3.37, 6.03) | 4.358e-24 | - | 4.36 (3.16, 6.02) | - | - | 4.725 (0.879, 25.408) | 2/1.9997 | 1 | Weak |
|  |  | Europe | 2 | 310/753 | OR | 2.30 (1.49, 3.55) | 0.00017477 | - | 2.40 (1.50, 3.80) | - | - | 1.989 (0.806, 4.913) | 1/1.9974 | 0.002598 | Weak |
|  | Zeng, 2013 | Asia | 2 | 833/2,095 | OR | 2.81 (0.68, 11.66) | 0.15405306 | - | 1.40 (1.00, 1.97) | - | - | 1.515 (0.794, 2.890) | 1/2 | 2.2e-16 | Not significance |
|  |  | Europe | 5 | 1,339/3,259 | OR | 1.71 (0.83, 3.52) | 0.14210335 | 79 (32, 89) | 2.11 (1.40, 3.18) | (0.15, 20.19) | 0.545 | 1.219 (0.694, 2.143) | 2/4.5058 | 0.008281 | Not significance |
|  |  | America | 2 | 1,507/51,160 | OR | 1.26 (0.99, 1.59) | 0.05777896 | - | 1.21 (0.94, 1.56) | - | - | 1.259 (0.961, 1.649) | 0/1.231 | 0.1478 | Not significance |
| **Oral cancer** | | | | | | | | | | | | | | | |
| **Periodontal diseases** | Li, 2023 | Asia | 3 | 1,143/1,609 | OR | 4.06 (1.80, 9.15) | 0.00073928 | 90 (67, 95) | 2.27 (1.64, 3.13) | (0, 93202.33) | 0.798 | 2.963 (1.065, 8.244) | 3/3 | TRUE | Suggestive |
|  | Ma, 2024 | Asia | 2 | 1,438/4,300 | OR | 2.69 (1.52, 4.77) | 0.00069334 | - | 2.22 (1.07, 4.60) | - | - | 2.546 (0.901, 7.197) | 2/2 | TRUE | Suggestive |
|  |  | Europe | 2 | 310/753 | OR | 2.30 (1.49, 3.55) | 0.00017477 | - | 2.40 (1.50, 3.80) | - | - | 1.989 (0.806, 4.913) | 1/1.9974 | 0.002598 | Weak |
|  |  | America | 4 | 1,269/2,465 | OR | 3.38 (2.02, 5.65) | 3.429e-06 | 69 (0, 86) | 4.36 (3.16, 6.01) | (0.63, 18.08) | 0.975 | 2.292 (1.212, 4.334) | 4/4.9498 | 0.0492 | Suggestive |
|  | Mahuili, 2023 | Asia | 6 | 1,817/3,815 | OR | 3.36 (2.71, 4.15) | 5.283e-29 | 0 (0, 58) | 3.94 (2.49, 6.25) | (2.54, 4.44) | 0.488 | 3.034 (1.580, 5.826) | 7/7 | TRUE | Convincing |
|  |  | America | 7 | 2,688/4,553 | OR | 1.83 (1.14, 2.95) | 0.01216681 | 93 (90, 95) | 1.07 (1.00, 1.13) | (0.36, 9.31) | 0.130 | 1.108 (0.985, 1.245) | 3/6.278 | 0.003062 | Weak |
|  |  | Europe | 3 | 432/827 | OR | 2.66 (1.75, 4.03)) | 4.419e-06 | 0 (0, 73) | 2.40 (1.50, 3.80) | (0.18, 39.70) | 0.094 | 3.028 (1.107, 8.282) | 2/2.9996 | 0.0003999 | Suggestive |
|  | Yao, 2014 | America | 3 | 1,009/2,631 | OR | 2.96 (1.08, 8.05) | 0.03406405 | 95 (90, 97) | 1.22 (0.94, 1.58) | (0, 935569.45) | 0.570 | 1.563 (0.733, 3.336) | 2/2.9722 | 0.02754 | Weak |
| **Lung cancer** | | | | | | | | | | | | | | | |
| **Periodontal diseases** | Zhang, 2023 | America | 4 | 2,089/200,432 | HR | 1.45 (1.07, 1.96) | 0.01519312 | 45 (0, 78) | 1.31 (1.14, 1.51) | (0.64, 3.31) | 0.491 | 1.291 (0.938, 1.776) | 2/3.7309 | 0.1076 | Weak |
|  | Chen, 2020 | America | 5 | 2,353/135,053 | RR | 1.45 (1.30, 1.63) | 1.341e-10 | 21 (0, 71) | 1.31 (1.14, 1.51) | (1.11, 1.90) | 0.146 | 1.447 (1.111, 1.886) | 5/4.4852 | 1 | Convincing |
|  |  | Europe | 2 | 225/68,473 | RR | 1.60 (0.74, 3.45) | 0.22748844 | - | 1.20 (0.81, 1.80) | - | - | 1.277 (0.835, 1.952) | 1/1.3147 | 1 | Not significance |
|  | Zeng, 2016 | America | 3 | 1,181/137,188 | HR | 1.32 (1.18, 1.48) | 1.667e-06 | 0 (0, 73) | 1.25 (1.06, 1.47) | (0.63, 2.76) | 0.384 | 1.325 (1.019, 1.722) | 3/2.02 | 0.5557 | Highly suggestive |
|  | Wang, 2020 | America | 6 | 2,165/136,390 | HR | 1.43 (1.26, 1.62) | 1.577e-08 | 10 (0, 65) | 1.31 (1.14, 1.51) | (1.14, 1.80) | 0.085 | 1.430 (1.124, 1.820) | 4/4.513 | 0.642 | Highly suggestive |
|  |  | Europe | 2 | 289/30,866 | HR | 1.74 (0.95, 3.17) | 0.07082979 | - | 1.41 (0.81, 2.46) | - | - | 1.522 (0.903, 2.566) | 1/1.5868 | 0.3705 | Not significance |
|  | Verma, 2023 | America | 5 | 6,142/29,370 | RR | 1.60 (1.23, 2.08) | 0.0003933 | 84 (55, 91) | 1.35 (1.14, 1.60) | (0.63, 4.05) | 0.954 | 1.383 (1.087, 1.760) | 4/4.3041 | 0.5273 | Suggestive |
|  |  | Europe | 2 | 17,669/78,686 | RR | 1.31 (1.20, 1.44) | 1.232e-08 | - | 1.31 (1.19, 1.44) | - | - | 1.347 (0.974, 1.864) | 1/1.8968 | 0.1005 | Highly suggestive |
|  | Duan, 2026 | Asian | 3 | 4,673/986,029 | RR | 1.30 (0.80, 2.13) | 0.2939225 | 98 (97, 99) | 0.87 (0.79, 0.95) | (0, 737.24) | 0.013 | 1.032 (0.789, 1.349) | 3/2.3328783 | 1 | Not significance |
|  |  | American | 3 | 1,450/127,197 | RR | 1.31 (1.11, 1.53) | 0.00100836 | 1 (0, 73) | 1.36 (1.15, 1.60) | (0.44, 3.88) | 0.934 | 1.172 (0.865, 1.588) | 1/1.9635336 | 0.2756 | Weak |
| **Periodontitis** | Kesharani, 2022 | America | 7 | 3,212/231,679 | RR | 2.04 (1.62, 2.56) | 9.474e-10 | 88 (77, 92) | 1.83 (1.60, 2.09) | (0.94, 4.40) | 0.752 | 1.527 (1.124, 2.075) | 3/6.9658 | 1.971e-08 | Highly suggestive |
|  |  | Asia | 3 | 1,572/165,177 | RR | 1.32 (1.11, 1.57) | 0.00163242 | 56 (0, 86) | 1.23 (1.09, 1.38) | (0.22, 8.00) | 0.074 | 1.278 (1.008, 1.621) | 0/2.1156 | 0.02562 | Weak |
|  |  | Europe | 2 | 253/13,654 | RR | 1.16 (0.84, 1.60) | 0.36489936 | - | 1.19 (0.80, 1.79) | - | - | 1.161 (0.841, 1.601) | 0/0.2596 | 1 | Not significance |
|  | Wang, 2022 | America | 7 | 3,074/243,469 | RR | 1.38 (1.21, 1.57) | 1.230e-06 | 50 (0, 77) | 1.31 (1.14, 1.51) | (0.98, 1.93) | 0.230 | 1.292 (1.069, 1.561) | 6/5.1673 | 0.6845 | Suggestive |
|  |  | Asia | 6 | 1,491/754,010 | RR | 1.11 (0.77, 1.59) | 0.56613829 | 61 (0, 82) | 1.08 (0.92, 1.27) | (0.41, 2.99) | 0.643 | 1.053 (0.763, 1.455) | 1/0.7412 | 0.5467 | Not significance |
| **Breast cancer** | | | | | | | | | | | | | | | |
| **Periodontal diseases** | Shao, 2018 | America | 4 | 2,786/78,156 | RR | 1.11 (1.03, 1.21) | 0.00899304 | 0 (0, 68) | 1.13 (1.03, 1.23) | (0.93, 1.33) | 0.767 | 1.086 (0.946, 1.247) | 1/0.9774 | 1 | Weak |
|  |  | Europe | 3 | 644/50,127 | RR | 1.92 (0.78, 4.72) | 0.15767998 | 81 (0, 92) | 1.12 (0.75, 1.68) | (0, 69706.92) | 0.202 | 1.162 (0.835, 1.618) | 1/2.3319 | 0.1267 | Not significance |
|  |  | Asia | 3 | 456/44,678 | RR | 1.27 (1.08, 1.50) | 0.0048249 | 6 (0, 74) | 1.23 (1.11, 1.36) | (0.31, 5.19) | 0.176 | 1.319 (0.996, 1.746) | 1/0.868 | 1 | Weak |
|  | Shi, 2018 | America | 3 | 2,552/70,757 | RR | 1.14 (1.05, 1.24) | 0.00304268 | 0 (0, 73) | 1.13 (1.03, 1.23) | (0.65, 1.99) | 0.072 | 1.166 (0.986, 1.378) | 1/1.0516 | 1 | Weak |
|  |  | Europe | 2 | 545/15,095 | RR | 1.50 (0.73, 3.06) | 0.26554727 | - | 1.12 (0.75, 1.68) | - | - | 1.238 (0.752, 2.036) | 1/1.2492 | 1 | Not significance |
|  |  | Asia | 2 | 374/80,431 | RR | 1.47 (0.82, 2.62) | 0.19244039 | - | 1.23 (1.11, 1.36) | - | - | 1.274 (0.936, 1.734) | 1/0.6792 | 1 | Not significance |
|  | Duan, 2026 | America | 6 | 13,105/332,245 | RR | 1.07 (1.02, 1.12) | 0.00587173 | 13 (0, 66) | 1.02 (0.94, 1.10) | (0.98, 1.17) | 0.591 | 1.039 (0.986, 1.094) | 2/0.47577394 | 0.07608 | Weak |
| **Periodontitis** | Wang, 2022 | Asia | 6 | -/79,904 | HR | 1.40 (1.08, 1.82) | 0.01190043 | 84 (63, 91) | 1.23 (1.11, 1.36) | (0.62, 3.17) | 0.475 | 1.097 (0.969, 1.242) | 3/5.8038 | 0.0006492 | Weak |
|  |  | America | 3 | -/147,072 | HR | 1.12 (1.05, 1.19) | 0.00077959 | 0 (0, 73) | 1.13 (1.04, 1.23) | (0.73, 1.70) | 0.043 | 1.103 (0.980, 1.241) | 2/2.9983 | 0.001699 | Suggestive |
| **Esophagus cancer** | | | | | | | | | | | | | | | |
| **Periodontal diseases** | Duan, 2026 | Asia | 3 | 562/938,856 | RR | 0.98 (0.81, 1.19) | 0.82055536 | 0 (0, 73) | 0.88 (0.67, 1.16) | (0.28, 3.40) | 0.788 | 0.978 (0.807, 1.185) | 0/0.48755714 | 1 | Not significance |
|  |  | America | 3 | 349/262,388 | RR | 1.24 (0.98, 1.58) | 0.07723987 | 52 (0, 85) | 1.04 (0.86, 1.26) | (0.10, 14.94) | 0.184 | 1.119 (0.926, 1.351) | 1/0.16883509 | 0.1595 | Not significance |
| **Periodontitis** | Ma, 2020 | America | 3 | -/126,849 | HR | 2.28 (1.16, 4.48) | 0.01678927 | 63 (0, 87) | 1.44 (0.98, 2.11) | (0, 3498.75) | 0.331 | 1.637 (0.978, 2.742) | 2/2 | 1 | Weak |
| **Gastric cancer** | | | | | | | | | | | | | | | |
| **Periodontal diseases** | Aguiar, 2024 | Asia | 6 | 7,127/253,374 | RR | 1.17 (1.00, 1.36) | 0.04509338 | 56 (0, 80) | 1.23 (1.18, 1.29) | (0.77, 1.77) | 0.664 | 1.067 (0.934, 1.219) | 2/2.5253 | 1 | Weak |
|  |  | Europe | 3 | 362/129,577 | RR | 1.18 (0.84, 1.66) | 0.34810514 | 13 (0, 76) | 1.58 (0.94, 2.67) | (0.09, 16.22) | 0.367 | 1.117 (0.786, 1.587) | 0/0.4391 | 1 | Not significance |
|  | Duan, 2026 | Asian | 3 | 7,244/938,856 | RR | 1.10 (1.05, 1.17) | 0.0003956 | 0 (0, 73) | 1.08 (1.00, 1.16) | (0.77, 1.58) | 0.777 | 1.089 (0.987, 1.201) | 2/1.4955354 | 0.6239 | Suggestive |
|  |  | American | 3 | 482/245,639 | RR | 1.42 (1.15, 1.74) | 0.00091559 | 0 (0, 73) | 1.52 (1.13, 2.04) | (0.37, 5.36) | 0.268 | 1.307 (0.955, 1.787) | 2/1.1450372 | 0.5622 | Weak |
| **Periodontitis** | Zhang, 2020 | America | 2 | 169/114,244 | HR | 1.31 (0.93, 1.84) | 0.12713037 | - | 1.13 (0.72, 1.79) | - | - | 1.249 (0.852, 1.829) | 0/0.4716 | 1 | Not significance |
| **Pancreatic cancer** | | | | | | | | | | | | | | | |
| **Periodontal diseases** | Maisonneuv, 2017 | America | 3 | 278/72,308 | RR | 1.72 (1.29, 2.29) | 0.00024517 | 0 (0, 73) | 1.63 (1.19, 2.26) | (0.26, 11.16) | 0.289 | 1.793 (1.029, 3.124) | 1/1.6236 | 0.5969 | Weak |
|  |  | Europe | 2 | 268/35,257 | RR | 2.07 (1.29, 3.33) | 0.00243236 | - | 2.06 (1.14, 3.75) | - | - | 2.079 (0.942, 4.590) | 1/1.9743 | 0.02553 | Weak |
|  | Duan, 2026 | Asia | 4 | 1,226/1,153,746 | RR | 1.08 (0.92, 1.27) | 0.36630839 | 20 (0, 74) | 1.04 (0.85, 1.27) | (0.66, 1.75) | 0.659 | 1.039 (0.902, 1.195) | 1/0.26687298 | 0.2413 | Not significance |
|  |  | America | 4 | 613/224,773 | RR | 1.33 (0.88, 2.00) | 0.17781402 | 85 (53, 93) | 0.89 (0.77, 1.02) | (0.22, 7.93) | 0.254 | 1.210 (0.806, 1.817) | 2/0.50171308 | 0.07935 | Not significance |
| **Colorectal cancer** | | | | | | | | | | | | | | | |
| **Periodontal diseases** | Xuan, 2021 | America | 6 | 9,953/220,423 | RR | 1.00 (0.91, 1.10) | 0.98748066 | 0 (0, 61) | 1.05 (0.90, 1.23) | (0.88, 1.14) | 0.236 | 1.001 (0.912, 1.098) | 0/- | - | Not significance |
|  |  | Asia | 4 | 112,365/375,055 | RR | 1.40 (1.21, 1.61) | 4.126e-06 | 84 (48, 92) | 1.25 (1.15, 1.36) | (0.73, 2.67) | 0.962 | 1.327 (1.060, 1.661) | 4/3.6671 | 1 | Suggestive |
|  | Li, 2021 | America | 5 | 1,430/109,587 | HR | 1.27 (0.98, 1.65) | 0.0676668 | 28 (0, 73) | 1.22 (0.91, 1.63) | (0.67, 2.41) | 0.103 | 1.181 (0.965, 1.446) | 1/2.1561 | 0.3984 | Not significance |
|  | Xu, 2025 | America | 5 | 23,630/121,756 | OR | 1.37 (0.93, 2.04) | 0.11431657 | 82 (45, 90) | 1.06 (0.89, 1.27) | (0.37, 5.10) | 0.557 | 1.087 (0.950, 1.243) | 1/4.6400 | 0.0001266 | Not significance |
|  | Duan, 2026 | Asia | 6 | 3,314/219,013 | RR | 1.26 (0.96, 1.66) | 0.09349928 | 73 (14, 86) | 1.05 (0.90, 1.23) | (0.56, 2.86) | 0.085 | 1.02 (0.902, 1.155) | 2/3.9100873 | 0.1922 | Not significance |
|  |  | America | 4 | 7,703/1,045,343 | RR | 1.17 (0.92, 1.48) | 0.19411354 | 95 (92, 97) | 1.04 (0.96, 1.13) | (0.38, 3.64) | 0.798 | 1.044 (0.974, 1.120) | 2/0.63103179 | 0.1198 | Not significance |
| **Periodontitis** | Wang, 2022 | America | 6 | -/279,035 | HR | 1.10 (0.97, 1.25) | 0.1424952 | 27 (0, 71) | 1.05 (0.90, 1.23) | (0.82, 1.47) | 0.026 | 1.059 (0.957, 1.171) | 1/5.9845 | 6.888e-13 | Not significance |
|  |  | Asia | 6 | -/358,200 | HR | 1.16 (0.94, 1.43) | 0.16947119 | 85 (68, 91) | 1.25 (1.17, 1.34) | (0.63, 2.12) | 0.429 | 1.016 (0.877, 1.177) | 2/6.7738 | 7.006e-07 | Not significance |
|  | Espejo-Carrera, 2025 | America | 6 | 3,017/211,226 | RR | 1.13 (0.97, 1.33) | 0.12426045 | 57 (0, 81) | 1.34 (1.15, 1.55) | (0.72, 1.77) | 0.792 | 1.045 (0.929, 1.175) | 1/2.189 | 0.4262 | Not significance |
| **Bladder cancer** | | | | | | | | | | | | | | | |
| **Periodontal diseases** | Xie, 2018 | America | 2 | 732/114,244 | HR | 1.15 (0.97, 1.36) | 0.10350626 | - | 1.17 (0.96, 1.43) | - | - | 1.143 (0.946, 1.380) | 0/0.5289 | 1 | Not significance |
|  | Duan, 2026 | Asia | 2 | 1,101/913,371 | RR | 1.20 (0.97, 1.50) | 0.0962403 | - | 1.31 (1.08, 1.59) | - | - | 1.127 (0.883, 1.439) | 1/1.8697402 | 0.126 | Not significance |
|  |  | America | 3 | 1,525/159,429 | RR | 1.26 (1.09, 1.47) | 0.00240374 | 26 (0, 79) | 1.43 (1.18, 1.74) | (0.34, 4.64) | 0.527 | 1.180 (0.978, 1.425) | 1/2.3523319 | 0.1197 | Weak |
| **Prostate cancer** | | | | | | | | | | | | | | | |
| **Periodontal diseases** | Wei, 2021 | Asia | 2 | 1,784/230,986 | RR | 1.18 (1.04, 1.34) | 0.01245616 | - | 1.14 (1.01, 1.31) | - | - | 1.171 (0.975, 1.406) | 2/1.3901 | 1 | Weak |
|  |  | America | 3 | 1,138/27,777 | RR | 1.10 (0.94, 1.29) | 0.25042498 | 10 (0, 76) | 1.01 (0.82, 1.23) | (0.32, 3.75) | 0.383 | 1.092 (0.932, 1.279) | 0/0.4491 | 1 | Not significance |
|  |  | Europe | 2 | 630/35,636 | RR | 1.50 (1.08, 2.07) | 0.01545451 | - | 1.47 (1.04, 2.07) | - | - | 1.535 (0.921, 2.558) | 1/1.1759 | 1 | Weak |
|  | Guo, 2021 | Asia | 5 | 1,611/386,851 | HR | 1.51 (1.09, 2.09) | 0.01300463 | 87 (70, 93) | 1.14 (1.01, 1.31) | (0.48, 4.79) | 0.214 | 1.119 (0.934, 1.339) | 4/3.1942 | 0.6599 | Weak |
|  |  | America | 3 | 1,138/38,727 | HR | 1.22 (1.03, 1.45) | 0.02265429 | 0 (0, 73) | 1.17 (0.94, 1.47) | (0.40, 3.69) | 0.107 | 1.218 (1.006, 1.476) | 0/1.3585 | 0.2567 | Weak |
|  | Li, 2022 | America | 3 | 1,138/27,777 | HR | 1.23 (1.06, 1.43) | 0.00576287 | 0 (0, 73) | 1.25 (1.01, 1.53) | (0.47, 3.22) | 0.326 | 1.220 (1.008, 1.476) | 1/1.4302 | 1 | Weak |
|  |  | Europe | 2 | 630/44,008 | HR | 1.20 (0.78, 1.84) | 0.59334916 | - | 1.47 (1.04, 2.07) | - | - | 1.120 (0.740, 1.695) | 1/0.6778 | 1 | Not significance |
|  |  | Asia | 2 | 4,472/201,520 | HR | 1.25 (1.17, 1.33) | 8.206e-12 | - | 1.24 (1.16, 1.32) | - | - | 1.274 (0.977, 1.661) | 2/1.902 | 1 | Highly suggestive |
|  | Duan, 2026 | America | 2 | 1,300/68,308 | RR | 1.17 (1.01, 1.36) | 0.03725091 | - | 1.17 (0.96, 1.43) | - | - | 1.170 (0.987, 1.387) | 0/1.167905 | 0.1731 | Weak |
| **Kidney cancer** | | | | | | | | | | | | | | | |
| **Periodontal diseases** | Duan, 2026 | Asia | 2 | 907/913,371 | RR | 1.06 (0.79, 1.44) | 0.68239735 | - | 0.92 (0.76, 1.12) | - | - | 1.037 (0.774, 1.390) | 0/0.33058287 | 1 | Not significance |
|  |  | America | 2 | 290/114,244 | RR | 1.30 (0.96, 1.76) | 0.09035193 | - | 1.49 (1.12, 1.98) | - | - | 1.182 (0.867, 1.611) | 1/1.2258067 | 1 | Not significance |
| **Hematopoietic and lymphatic cancers** | | | | | | | | | | | | | | | |
| **Periodontal diseases** | Wu, 2020 | America | 4 | 2,042/130,537 | HR | 1.01 (0.84, 1.22) | 0.90290353 | 48 (0, 74) | 1.30 (1.11, 1.51) | (0.64, 1.61) | 0.137 | 1.019 (0.871, 1.191) | 1/0.2585 | 0.2307 | Not significance |
|  | Duan, 2026 | Asia | 2 | 637/242,408 | RR | 1.50 (0.65, 3.46) | 0.34233124 | - | 0.98 (1.11, 1.52) | - | - | 1.175 (0.593, 2.327) | 1/0.10910401 | 0.1061 | Not significance |
|  |  | America | 2 | 899/52,866 | RR | 1.31 (1.12, 1.53) | 0.00063099 | - | 1.30 (1.11, 1.52) | - | - | 1.371 (0.940, 2.001) | 1/1.0973148 | 1 | Weak |

**Abbreviation:** OR, odds ratio; RR, risk ratio; HR, hazard ratio; N, included original studies; ES, estimate size; CI, confidence interval; PI, predictive interval.

**STable 8. Subgroup analysis of periodontal diseases and cancer stratified by adjustment variables.**

| **Exposure** | **Authors, publication year** | **Adjustment variables** | **N studies** | **Cases/Sample size** | **Random-effect model** | | | **I^2^ (%)** | **Largest study**  **ES (95% CI)** | **95% PI** | **Egger’s P value** | **10% Credibility ceiling** | **Excess significance** | | **Evidence grade** |
| --- | --- | --- | --- | --- | --- | --- | --- | --- | --- | --- | --- | --- | --- | --- | --- |
|  |  |  |  |  | **ES** | **ES (95% CI)** | **P value** |  |  |  |  |  | **O/E** | **P value** |  |
| **Head and neck cancer** | | | | | | | | | | | | | | | |
| **Periodontal diseases** | Gopinath, 2020 | Smoking and alcohol | 7 | 1,114/2,464 | OR | 3.19 (2.09, 4.85) | 6.386e-08 | 68 (0, 84) | 4.36 (3.16, 6.01) | (0.93, 10.86) | 0.799 | 2.250 (1.294, 3.914) | 6/6.9455 | 0.05324 | Highly suggestive |
|  | Zeng, 2013 | Adjustment for covariates | 8 | 4,892/71,622 | OR | 2.46 (1.62, 3.74) | 0.00002536 | 88 (77, 92) | 1.33 (1.07, 1.65) | (0.60, 10.08) | 0.280 | 1.416 (1.069, 1.876) | 6/7.892 | 0.004834 | Suggestive |
| **Oral cancer** | | | | | | | | | | | | | | | |
| **Periodontal diseases** | Li, 2023 | Smoking and alcohol | 4 | 1,718/2,338 | OR | 2.51 (1.76, 3.58) | 3.910e-07 | 55 (0, 83) | 2.27 (1.64, 3.13) | (0.63, 9.93) | 0.250 | 2.109 (1.139, 3.904) | 4/3.9929 | 1 | Highly suggestive |
|  | Ma, 2024 | Smoking and alcohol | 6 | 1,280/2,867 | OR | 3.21 (2.11, 4.87) | 4.661e-08 | 57 (0, 80) | 4.36 (3.16, 6.01) | (1.01, 10.23) | 0.755 | 2.238 (1.300, 3.855) | 5/6.9319 | 0.001924 | Highly suggestive |
|  | Yao, 2014 | Smoking and alcohol | 4 | 1,141/3,083 | OR | 2.66 (1.12, 6.36) | 0.02735498 | 93 (86, 96) | 1.22 (0.94, 1.58) | (0.05, 147.66) | 0.659 | 1.294 (0.968, 1.731) | 2/3.9373 | 0.001444 | Weak |
|  | Ye, 2016 | Smoking and alcohol | 9 | 1,270/3,851 | OR | 2.16 (1.29, 3.61) | 0.00348239 | 92 (87, 94) | 1.06 (1.01, 1.13) | (0.35, 13.15) | 0.027 | 1.278 (0.998, 1.638) | 7/8.9461 | 0.001256 | Weak |
| **Lung cancer** | | | | | | | | | | | | | | | |
| **Periodontal diseases** | Zhang, 2023 | Never smokers | 5 | 1,851/120,865 | HR | 1.00 (0.76, 1.31) | 0.98010098 | 7 (0, 61) | 0.89 (0.59, 1.33) | (0.64, 1.56) | 0.670 | 0.949 (0.736, 1.225) | 1/- | - | Not significance |
|  |  | Smoking | 5 | 4,337/8,806 | HR | 1.36 (1.12, 1.64) | 0.00155538 | 58 (0, 81) | 1.13 (1.01, 1.26) | (0.83, 2.22) | 0.113 | 1.206 (1.028, 1.415) | 3/4.3507 | 0.356 | Weak |
|  | Zeng, 2016 | Smoking and alcohol | 4 | 1,406/167,854 | HR | 1.32 (1.18, 1.48) | 7.979e-07 | 0 (0, 68) | 1.25 (1.06, 1.47) | (1.04, 1.69) | 0.293 | 1.340 (1.057, 1.699) | 3/2.5687 | 1 | Highly suggestive |
| **Periodontitis** | Wang, 2022 | Smoking and alcohol | 7 | 2,236/200,548 | RR | 1.40 (1.16, 1.68) | 0.00034072 | 57 (0, 80) | 1.36 (1.16, 1.60) | (0.86, 2.28) | 0.744 | 1.252 (1.015, 1.544) | 5/4.3896 | 1 | Suggestive |
| **Breast cancer** | | | | | | | | | | | | | | | |
| **Periodontal diseases** | Shi, 2018 | Smoking | 3 | 2,464/67,615 | RR | 1.64 (0.91, 2.97) | 0.10269453 | 66 (0, 88) | 1.13 (1.03, 1.23) | (0, 1258.84) | 0.039 | 1.275 (0.860, 1.890) | 2/1.4493 | 0.6128 | Not significance |
| **Periodontitis** | Wang, 2022 | Smoking and alcohol | 4 | -/119,484 | HR | 1.11 (1.01, 1.22) | 0.03228955 | 0 (0, 68) | 1.11 (1.00, 1.23) | (0.90, 1.37) | 0.987 | 1.108 (0.965, 1.273) | 1/3.9946 | 9.832e-09 | Weak |
| **Esophagus cancer** | | | | | | | | | | | | | | | |
| **Periodontitis** | Ma, 2020 | Smoking and alcohol | 2 | -/114,244 | HR | 2.06 (0.93, 4.57) | 0.07573793 | - | 1.44 (0.98, 2.11) | - | - | 1.546 (0.907, 2.634) | 1/2 | 2.2e-16 | Not significance |
| **Gastric cancer** | | | | | | | | | | | | | | | |
| **Periodontitis** | Zhang, 2020 | Smoking and alcohol | 2 | 169/114,244 | HR | 1.31 (0.93, 1.84) | 0.12713037 | - | 1.13 (0.72, 1.79) | - | - | 1.249 (0.852, 1.829) | 0/0.4716 | 1 | Not significance |
| **Pancreatic cancer** | | | | | | | | | | | | | | | |
| **Periodontal diseases** | Maisonneuv, 2017 | Smoking and alcohol | 2 | 268/35,257 | RR | 2.07 (1.29, 3.33) | 0.00243236 | - | 2.06 (1.14, 3.75) | - | - | 2.079 (0.942, 4.590) | 1/1.9743 | 0.02553 | Weak |
| **Colorectal cancer** | | | | | | | | | | | | | | | |
| **Periodontal diseases** | Xuan, 2021 | Smoking and alcohol | 6 | 14,654/257,357 | RR | 1.10 (0.95, 1.27) | 0.19717611 | 71 (5, 86) | 1.25 (1.15, 1.36) | (0.71, 1.71) | 0.477 | 1.019 (0.928, 1.118) | 2/2.7387 | 0.6946 | Not significance |
|  | Li, 2021 | Smoking and alcohol | 4 | 1,120/92,382 | HR | 1.39 (1.13, 1.71) | 0.00159158 | 0 (0, 68) | 1.22 (0.91, 1.63) | (0.89, 2.19) | 0.465 | 1.320 (1.024, 1.701) | 1/2.299 | 0.3186 | Weak |
| **Periodontitis** | Wang, 2022 | Smoking and alcohol | 8 | -/294,318 | HR | 1.21 (1.08, 1.35) | 0.00067318 | 35 (0, 70) | 1.25 (1.17, 1.34) | (0.94, 1.56) | 0.888 | 1.117 (0.999, 1.249) | 3/8 | 2.2e-16 | Suggestive |
|  | Espejo-Carrera, 2025 | Smoking and alcohol | 6 | 2,728/204,796 | RR | 1.15 (0.96, 1.37) | 0.14032832 | 60 (0, 82) | 1.34 (1.15, 1.55) | (0.69, 1.90) | 0.652 | 1.040 (0.914, 1.184) | 1/1.9939 | 0.6708 | Not significance |
| **Bladder cancer** | | | | | | | | | | | | | | | |
| **Periodontal diseases** | Xie, 2018 | Smoking and alcohol | 2 | 717/79,041 | HR | 1.17 (0.96, 1.41) | 0.1130248 | - | 1.17 (0.96, 1.43) | - | - | 1.165 (0.930, 1.459) | 0/0.6256 | 1 | Not significance |
| **Prostate cancer** | | | | | | | | | | | | | | | |
| **Periodontal diseases** | Wei, 2021 | Smoking and alcohol | 3 | 1,913/198,273 | RR | 1.14 (0.98, 1.33) | 0.09670855 | 42 (0, 83) | 1.14 (1.01, 1.31) | (0.25, 5.21) | 0.684 | 1.093 (0.951, 1.255) | 2/1.1247 | 0.5605 | Not significance |
|  | Guo, 2021 | Smoking and alcohol | 4 | 1,980/222,061 | HR | 1.20 (1.07, 1.34) | 0.00148464 | 0 (0, 68) | 1.14 (1.01, 1.31) | (0.94, 1.52) | 0.061 | 1.205 (1.024, 1.419) | 2/1.9412 | 1 | Weak |
|  | Li, 2022 | Smoking and alcohol | 3 | 4,601/139,951 | HR | 1.25 (1.17, 1.33) | 9.576e-13 | 0 (0, 73) | 1.24 (1.16, 1.32) | (0.84, 1.85) | 0.404 | 1.274 (1.023, 1.587) | 3/2.3624 | 1 | Highly suggestive |
| **Hematopoietic and lymphatic cancers** | | | | | | | | | | | | | | | |
| **Periodontal diseases** | Wu, 2020 | Smoking | 3 | 1,167/84,390 | HR | 0.93 (0.76, 1.15) | 0.51081765 | 26 (0, 67) | 1.11 (0.95, 1.30) | (0.60, 1.45) | 0.278 | 0.984 (0.834, 1.161) | 0/0.4818 | 1 | Not significance |

**Abbreviation:** OR, odds ratio; RR, risk ratio; HR, hazard ratio; N, included original studies; ES, estimate size; CI, confidence interval; PI, predictive interval.

**STable 9. Subgroup analysis of periodontal diseases and cancer stratified by the method used to evaluate periodontal diseases.**

| **Exposure** | **Authors, publication year** | **Evaluation method** | **N studies** | **Cases/Sample size** | **Random-effect model** | | | **I^2^ (%)** | **Largest study**  **ES (95% CI)** | **95% PI** | **Egger’s P value** | **10% Credibility ceiling** | **Excess significance** | | **Evidence grade** |
| --- | --- | --- | --- | --- | --- | --- | --- | --- | --- | --- | --- | --- | --- | --- | --- |
|  |  |  |  |  | **ES** | **ES (95% CI)** | **P value** |  |  |  |  |  | **O/E** | **P value** |  |
| **Head and neck cancer** | | | | | | | | | | | | | | | |
| **Periodontal diseases** | Zeng, 2013 | ABL | 3 | 516/48,902 | OR | 2.11 (0.74, 5.98) | 0.1619433 | 91 (73, 95) | 4.36 (3.16, 6.02) | (0, 712994.01) | 0.684 | 1.260 (0.830, 1.915) | 1/2.972 | 0.0002597 | Not significance |
| **Alveolar bone loss** | Vu, 2021 | ABL | 4 | 586/1,303 | OR | 3.71 (2.60, 5.28) | 4.009e-13 | 42 (0, 80) | 4.36 (3.16, 6.02) | (1.04, 13.17) | 0.915 | 3.231 (1.280, 8.161) | 4/3.9967 | 1 | Weak |
| **Oral cancer** | | | | | | | | | | | | | | | |
| **Periodontal diseases** | Yao, 2014 | ABL | 3 | 449/1,030 | OR | 4.19 (2.86, 6.14) | 1.648e-13 | 21 (0, 78) | 4.36 (3.16, 6.02) | (0.15, 114.89) | 0.589 | 2.422 (0.900, 6.514) | 2/2.9992 | 0.0007998 | Weak |
| **Lung cancer** | | | | | | | | | | | | | | | |
| **Periodontal diseases** | Zhang, 2023 | Self-report | 3 | 1,852/160,024 | HR | 1.33 (1.16, 1.52) | 0.00004571 | 0 (0, 68) | 1.31 (1.14, 1.51) | (0.98, 1.79) | 0.089 | 1.397 (1.010, 1.933) | 1/2.6198 | 0.1218 | Suggestive |
|  |  | Clinical examination | 3 | 2,721/768,942 | HR | 1.30 (0.87, 1.93) | 0.20290947 | 78 (0, 91) | 1.13 (1.01, 1.26) | (0.01, 133.49) | 0.730 | 1.130 (0.948, 1.348) | 2/2.0268 | 1 | Not significance |
| **Periodontitis** | Wang, 2022 | Self-report | 5 | 2,795/220,211 | RR | 1.30 (1.20, 1.41) | 1.885e-10 | 0 (0, 64) | 1.31 (1.14, 1.51) | (1.14, 1.49) | 0.128 | 1.259 (1.038, 1.528) | 4/3.8749 | 1 | Highly suggestive |
|  |  | Clinical examination | 9 | 1,834/777,468 | RR | 1.45 (1.04, 2.01) | 0.02834585 | 75 (44, 86) | 1.08 (0.92, 1.27) | (0.53, 3.94) | 0.217 | 1.185 (0.889, 1.579) | 4/4.1609 | 1 | Weak |
| **Breast cancer** | | | | | | | | | | | | | | | |
| **Periodontal diseases** | Shao, 2018 | Clinical examination | 5 | 754/55,285 | RR | 1.42 (1.01, 1.99) | 0.04195989 | 75 (9, 88) | 1.23 (1.11, 1.36) | (0.48, 4.20) | 0.298 | 1.136 (0.924, 1.396) | 3/2.459 | 0.6825 | Weak |
| **Periodontitis** | Wang, 2022 | Self-report | 3 | -/154,939 | HR | 1.12 (1.05, 1.20) | 0.00045501 | 0 (0, 73) | 1.13 (1.04, 1.23) | (0.74, 1.70) | 0.858 | 1.119 (0.996, 1.256) | 2/3 | 2.2e-16 | Suggestive |
|  |  | Clinical examination | 8 | -/87,571 | HR | 1.35 (1.09, 1.68) | 0.00709955 | 80 (57, 88) | 1.23 (1.11, 1.36) | (0.69, 2.65) | 0.536 | 1.094 (0.972, 1.232) | 4/7.2754 | 0.003492 | Weak |
| **Gastric cancer** | | | | | | | | | | | | | | | |
| **Periodontal diseases** | Aguiar, 2024 | Clinical examination | 5 | 1,751/285,303 | RR | 1.12 (0.96, 1.31) | 0.1486552 | 36 (0, 76) | 1.23 (1.18, 1.29) | (0.74, 1.70) | 0.286 | 1.045 (0.888, 1.229) | 1/0.8996 | 1 | Not significance |
|  |  | Self-report | 4 | 5,738/97,648 | RR | 1.28 (0.94, 1.74) | 0.11093339 | 59 (0, 84) | 1.08 (0.87, 1.35) | (0.38, 4.32) | 0.786 | 1.115 (0.919, 1.353) | 1/2.3656 | 0.3116 | Not significance |
| **Colorectal cancer** | | | | | | | | | | | | | | | |
| **Periodontal diseases** | Xuan, 2021 | Self-report | 5 | 9,863/226,953 | RR | 1.04 (0.90, 1.61) | 0.56441463 | 51 (0, 80) | 1.05 (0.90, 1.23) | (0.68, 1.61) | 0.336 | 1.000 (0.908, 1.101) | 1/0.6677 | 0.5116 | Not significance |
|  |  | Clinical diagnosis (ICD-9,523) | 2 | 107,013/329,680 | RR | 1.48 (1.19, 1.84) | 0.00048429 | - | 1.64 (1.50, 1.80) | - | - | 1.379 (0.960, 1.982) | 2/1.9944 | 1 | Suggestive |
|  | Li, 2021 | Medical record | 5 | 3,213/302,628 | HR | 1.54 (1.27, 1.86) | 9.653e-06 | 29 (0, 74) | 1.64 (1.50, 1.80) | (0.94, 2.51) | 0.988 | 1.342 (1.043, 1.728) | 2/3.2091 | 0.3569 | Suggestive |
|  |  | Self-report | 2 | 671/35,26 | HR | 1.28 (0.82, 1.99) | 0.2773539 | - | 1.03 (0.75, 1.39) | - | - | 1.135 (0.788, 1.635) | 1/1.179 | 1 | Not significance |
|  | Wu, 2025 | Self-report | 3 | 15.849/71,165 | OR | 1.23 (0.93, 1.64) | 0.15410862 | 35 (0, 81) | 1.06 (0.89, 1.27) | (0.08, 19.43) | 0.016 | 1.138 (0.920, 1.406) | 0/1.8672 | 0.05384 | Not significance |
|  |  | Clinical examination | 4 | 8,499/62,421 | OR | 1.48 (0.76, 2.91) | 0.25154885 | 84 (43, 92) | 1.11 (0.89, 1.37) | (0.08, 27.57) | 0.767 | 1.124 (0.915, 1.381) | 1/3.8900 | 8.147e-05 | Not significance |
| **Periodontitis** | Wang, 2022 | Self-report | 6 | -/313,981 | HR | 1.15 (1.02, 1.30) | 0.02783682 | 61 (0, 82) | 1.25 (1.17, 1.34) | (0.81, 1.64) | 0.558 | 1.065 (0.966, 1.175) | 2/6 | 2.2e-16 | Weak |
|  |  | Clinical examination | 7 | -/338,587 | HR | 1.20 (0.91, 1.60) | 0.20101162 | 79 (54, 88) | 1.64 (1.49, 1.80) | (0.51, 2.82) | 0.238 | 1.014 (0.868, 1.18) | 2/7.7222 | 4.621e-08 | Not significance |
|  | Espejo-Carrera, 2025 | Self-report | 5 | 2,709/203,459 | RR | 1.13 (0.94, 1.35) | 0.18001867 | 64 (0, 84) | 1.34 (1.15, 1.55) | (0.64, 2.01) | 0.975 | 1.027 (0.910, 1.159) | 1/1.943 | 0.6549 | Not significance |
|  |  | Clinical examination | 3 | 3,551/720,594 | RR | 1.80 (0.96, 3.36) | 0.06760725 | 84 (7, 93) | 2.38 (2.17, 2.60) | (0, 2322.50) | 0.594 | 1.305 (0.851, 2.001) | 1/ 2.1346 | 0.2016 | Not significance |
| **Prostate cancer** | | | | | | | | | | | | | | | |
| **Periodontal diseases** | Wei, 2021 | Self-report | 2 | 1,300/26,894 | RR | 1.26 (1.02, 1.56) | 0.03045305 | - | 1.17 (0.94, 1.47) | - | - | 1.299 (0.968, 1.510) | 1/1.6724 | 0.3008 | Weak |
|  |  | Clinical examination | 5 | 2,252/267,505 | RR | 1.15 (1.02, 1.29) | 0.02000848 | 11 (0, 68) | 1.14 (1.01, 1.31) | (0.90, 1.46) | 0.223 | 1.117 (0.977, 1.276) | 2/1.5095 | 0.6415 | Weak |
|  | Guo,2021 | Self-report | 3 | 1,675/42,732 | HR | 1.25 (1.07, 1.46) | 0.00470436 | 0 (0, 73) | 1.17 (0.94, 1.47) | (0.46, 3.43) | 0.126 | 1.221 (1.013, 1.471) | 1/2.1997 | 0.1755 | Weak |
|  |  | Clinical and radiographic parameters | 3 | 977/194,383 | HR | 1.61 (0.99, 2.61) | 0.05591778 | 82 (0, 92) | 1.14 (1.01, 1.31) | (0.01, 393.22) | 0.276 | 1.245 (0.890, 1.740) | 3/1.3858 | 0.09857 | Not significance |
|  |  | Clinical diagnosis | 3 | 701/203,796 | HR | 1.52 (0.83, 2.79) | 0.17661797 | 90 (67, 95) | 1.00 (0.82, 1.23) | (0, 2217.59) | 0.760 | 1.242 (0.782, 1.973) | 1/2.2981 | 0.1386 | Not significance |
|  | Li, 2022 | Clinical examination | 5 | 4,940/238,039 | HR | 1.24 (1.17, 1.32) | 8.783e-13 | 0 (0, 64) | 1.24 (1.16, 1.32) | (1.13, 1.37) | 0.914 | 1.213 (1.012, 1.454) | 3/2.6483 | 1 | Convincing |
|  |  | Self-report | 2 | 1,300/35,266 | HR | 1.26 (1.02, 1.56) | 0.03045305 | - | 1.17 (0.94, 1.47) | - | - | 1.209 (0.968, 1.510) | 1/1.6724 | 0.3008 | Weak |

**Abbreviation:** OR, odds ratio; RR, risk ratio; HR, hazard ratio; N, included original studies; ES, estimate size; CI, confidence interval; PI, predictive interval.

**STable 9. Subgroup analysis of periodontal diseases and cancer stratified by follow-up duration.**

| **Exposure** | **Authors, publication year** | **Follow-up duration** | **N studies** | **Cases/Sample size** | **Random-effect model** | | | **I^2^ (%)** | **Largest study**  **ES (95% CI)** | **95% PI** | **Egger’s P value** | **10% Credibility ceiling** | **Excess significance** | | **Evidence grade** |
| --- | --- | --- | --- | --- | --- | --- | --- | --- | --- | --- | --- | --- | --- | --- | --- |
|  |  |  |  |  | **ES** | **ES (95% CI)** | **P value** |  |  |  |  |  | **O/E** | **P value** |  |
| **Gastric cancer** | | | | | | | | | | | | | | | |
| **Periodontal diseases** | Duan, 2026 | Short follow-up | 3 | 818/5,133,657 | RR | 1.19 (0.94, 1.52) | 0.15639378 | 46 (0, 84) | 1.04 (0.86, 1.26) | (0.11, 13.28) | 0.753 | 1.081 (0.909, 1.287) | 1/0.19488751 | 0.1825 | Not significance |
|  |  | Long follow-up | 4 | 851/1,109,890 | RR | 1.17 (0.92, 1.48) | 0.21003819 | 57 (0, 84) | 1.083 (0.868, 1.352) | (0.45, 3.01) | 0.228 | 1.083 (0.868, 1.352) | 1/0.71019665 | 0.5424 | Not significance |
| **Lung cancer** | | | | | | | | | | | | | | | |
| **Periodontal diseases** | Duan, 2026 | Short follow-up | 3 | 3,630/863,344 | RR | 1.40 (0.81, 2.40) | 0.22386561 | 96 (93, 98) | 1.13 (1.01, 1.26) | (0, 1258.78) | 0.923 | 1.124 (0.950, 1.330) | 2/2.8546075 | 0.1385 | Not significance |
|  |  | Long follow-up | 4 | 2,507/269,516 | RR | 1.17 (0.81, 1.69) | 0.40223468 | 87 (64, 93) | 0.87 (0.79, 0.95) | (0.26, 5.33) | 0.421 | 1.125 (0.803, 1.576) | 2/1.5343221 | 1 | Not significance |
| **Colorectal cancer** | | | | | | | | | | | | | | | |
| **Periodontal diseases** | Duan, 2026 | Short follow-up | 4 | 4,385/805,892 | RR | 1.38 (0.90, 2.10) | 0.13935591 | 71 (0, 88) | 1.13 (1.03, 1.24) | (0.25, 7.56) | 0.444 | 1.111 (0.941, 1.312) | 2/2.7090424 | 0.599 | Not significance |
|  |  | Long follow-up | 7 | 6,649/478,098 | RR | 1.23 (0.99, 1.53) | 0.06409646 | 92 (86, 94) | 1.04 (0.96, 1.13) | (0.59, 2.58) | 0.987 | 1.037 (0.971, 1.108) | 3/5.0446542 | 0.1008 | Not significance |
| **Pancreatic cancer** | | | | | | | | | | | | | | | |
| **Periodontal diseases** | Duan, 2026 | Short follow-up | 4 | 883/1,019,445 | RR | 1.05 (0.84, 1.30) | 0.69154725 | 40 (0, 79) | 1.01 (0.81, 1.25) | (0.48, 2.27) | 0.730 | 0.987 (0.839, 1.160) | 1/1.5182415 | 1 | Not significance |
|  |  | Long follow-up | 5 | 970/378,708 | RR | 1.45 (1.10, 1.91) | 0.00739591 | 63 (0, 84) | 1.04 (0.85, 1.27) | (0.62, 3.40) | 0.205 | 1.163 (0.945, 1.430) | 3/0.30247391 | 0.002018 | Weak |
| **Prostate cancer** | | | | | | | | | | | | | | | |
| **Periodontal diseases** | Duan, 2026 | Short follow-up | 2 | 1,072/236,309 | RR | 1.15 (1.03, 1.28) | 0.01297789 | - | 1.14 (1.00, 1.30) | - | - | 1.152 (0.988, 1.344) | 0/0.85843102 | 0.51 | Weak |
|  |  | Long follow-up | 2 | 797/39,567 | RR | 1.26 (1.02, 1.55) | 0.0305786 | - | 1.17 (0.94, 1.46) | - | - | 1.209 (0.968, 1.510) | 1/0.73269762 | 1 | Weak |
| **Esophageal cancer** | | | | | | | | | | | | | | | |
| **Periodontal diseases** | Duan, 2026 | Short follow-up | 3 | 1,166/979,240 | RR | 1.15 (0.98, 1.34) | 0.07815699 | 35 (0, 81) | 1.04 (0.87, 1.24) | (0.28, 4.78) | 0.999 | 1.083 (0.938, 1.250) | 1/0.21360841 | 0.1988 | Not significance |
|  |  | Long follow-up | 3 | 1,471/113,194 | RR | 1.30 (1.13, 1.49) | 0.00016447 | 0 (0, 73) | 1.43 (1.18, 1.74) | (0.54, 3.15) | 0.772 | 1.220 (0.974, 1.527) | 1/2.0959944 | 0.2177 | Suggestive |
| **Bladder cancer** | | | | | | | | | | | | | | | |
| **Periodontal diseases** | Duan, 2026 | Short follow-up | 3 | 1,166/979,240 | RR | 1.15 (0.98, 1.34) | 0.07815699 | 35 (0, 81) | 1.04 (0.87, 1.24) | (0.28, 4.78) | 0.999 | 1.083 (0.938, 1.250) | 1/0.21360841 | 0.1988 | Not significance |
|  |  | Long follow-up | 3 | 1,471/113,194 | RR | 1.30 (1.13, 1.49) | 0.00016447 | 0 (0, 73) | 1.43 (1.18, 1.74) | (0.54, 3.15) | 0.772 | 1.220 (0.974, 1.527) | 1/2.0959944 | 0.2177 | Suggestive |
| **Breast cancer** | | | | | | | | | | | | | | | |
| **Periodontal diseases** | Duan, 2026 | Short follow-up | 3 | 4,743/181,844 | RR | 1.16 (1.10, 1.23) | 1.201e-07 | 0 (0, 73) | 1.13 (1.03, 1.23) | (0.81, 1.67) | 0.551 | 1.149 (1.014, 1.303) | 3/1.928934 | 0.5572 | Highly suggestive |
|  |  | Long follow-up | 4 | 8,565/192,639 | RR | 1.02 (0.97, 1.08) | 0.42451864 | 0 (0, 68) | 1.02 (0.94, 1.10) | (0.90, 1.16) | 0.002 | 1.023 (0.967, 1.082) | 0/0.31456493 | 1 | Not significance |
| **Head and neck cancer** | | | | | | | | | | | | | | | |
| **Periodontal diseases** | Duan, 2026 | Short follow-up | 2 | 223/108,107 | RR | 1.20 (1.09, 1.32) | 0.00032487 | - | 1.20 (1.09, 1.33) | - | - | 1.178 (0.920, 1.509) | 1/0.37255083 | 0.3379 | Weak |
|  |  | Long follow-up | 3 | 1,230/961,746 | RR | 0.97 (0.86, 1.11) | 0.69701778 | 0 (0, 73) | 0.97 (0.84, 1.13) | (0.42, 2.25) | 0.681 | 0.975 (0.857, 1.109) | 0/0.19021621 | 1 | Not significance |
| **Hematopoietic and lymphatic cancers** | | | | | | | | | | | | | | | |
| **Periodontal diseases** | Duan, 2026 | Short follow-up | 2 | 962/93,767 | RR | 1.73 (0.99, 3.03) | 0.05465798 | - | 2.30 (1.98, 2.67) | - | - | 1.369 (0.933, 2.007) | 2/1.9900261 | 1 | Not significance |
|  |  | Long follow-up | 2 | 574/201,507 | RR | 0.98 (0.87, 1.10) | 0.69403155 | - | 0.98 (0.87, 1.10) | - | - | 0.977 (0.869, 1.098) | 0/0.10820005 | 1 | Not significance |

**STable 11. AMSTAR-2 assessment of 38 eligible articles.**

| **Author, year** | **PMID** | **Q1** | **Q2** | **Q3** | **Q4** | **Q5** | **Q6** | **Q7** | **Q8** | **Q9** | **Q10** | **Q11** | **Q12** | **Q13** | **Q14** | **Q15** | **Q16** | **Overall** |
| --- | --- | --- | --- | --- | --- | --- | --- | --- | --- | --- | --- | --- | --- | --- | --- | --- | --- | --- |
| Aguiar, 2024 | 38301538 | Y | Y | Y | Y | Y | Y | PY | Y | Y | Y | Y | Y | Y | Y | Y | Y | High |
| Bai, 2023 | 36538375 | Y | N | Y | PY | N | N | PY | Y | Y | N | Y | Y | Y | Y | Y | Y | Low |
| Chen, 2020 | 32802852 | Y | N | Y | Y | Y | Y | PY | Y | Y | Y | Y | Y | Y | Y | Y | Y | Low |
| Espejo-Carrera, 2025 | 40192116 | Y | N | Y | PY | Y | Y | PY | Y | Y | Y | Y | Y | Y | Y | Y | Y | Low |
| Gopinath, 2020 | 32674369 | Y | Y | Y | Y | Y | Y | PY | PY | Y | Y | Y | Y | Y | Y | Y | Y | High |
| Guo, 2021 | 33650836 | Y | N | Y | Y | Y | Y | PY | PY | Y | N | Y | Y | Y | Y | N | Y | Critically Low |
| Kesharani, 2022 | 36686995 | Y | Y | Y | Y | N | N | PY | PY | Y | Y | Y | Y | Y | Y | N | Y | Low |
| Li, 2021 | 33179280 | Y | N | Y | Y | N | N | Y | Y | Y | Y | Y | Y | Y | Y | Y | Y | Low |
| Li, 2022 | 36016605 | Y | Y | Y | Y | Y | Y | PY | Y | Y | Y | Y | Y | Y | Y | Y | Y | High |
| Li, 2023 | 37308334 | Y | Y | Y | Y | Y | Y | Y | Y | Y | Y | Y | N | Y | Y | Y | Y | High |
| Ma, 2020 | 33029095 | Y | N | Y | Y | Y | Y | PY | Y | Y | Y | Y | Y | Y | Y | Y | Y | Low |
| Ma, 2024 | 38742908 | Y | Y | Y | PY | Y | Y | PY | Y | Y | Y | Y | Y | Y | Y | Y | Y | High |
| Mahuili, 2023 | 37398714 | Y | Y | Y | Y | Y | Y | PY | PY | Y | N | Y | N | Y | Y | Y | Y | Moderate |
| Maisonneuve, 2017 | 28453689 | Y | N | Y | Y | Y | Y | N | PY | N | N | Y | N | Y | Y | Y | Y | Critically Low |
| Shao, 2018 | 30619743 | Y | N | Y | PY | Y | Y | PY | Y | N | N | Y | N | Y | Y | Y | Y | Critically Low |
| Shi, 2018 | 29974484 | Y | N | Y | Y | Y | Y | PY | PY | Y | N | Y | N | Y | Y | Y | Y | Low |
| Verma, 2023 | 37090288 | Y | N | N | PY | Y | Y | PY | PY | Y | Y | Y | Y | Y | Y | Y | Y | Low |
| Vu, 2021 | 33876587 | Y | N | Y | PY | N | N | Y | Y | Y | Y | Y | Y | Y | Y | Y | Y | Low |
| Wang, 2020 | 32583879 | Y | N | Y | Y | Y | N | PY | PY | Y | Y | Y | N | Y | Y | Y | Y | Low |
| Wang, 2022 | 36389427 | Y | Y | Y | Y | Y | Y | PY | Y | Y | Y | Y | N | Y | Y | Y | Y | High |
| Wang, 2024 | 39185624 | Y | Y | Y | Y | Y | Y | PY | Y | Y | Y | Y | Y | Y | Y | Y | Y | High |
| Wei, 2021 | 33247563 | Y | N | Y | Y | Y | Y | PY | Y | Y | Y | Y | Y | Y | Y | Y | Y | Low |
| Wu, 2020 | 31880294 | Y | N | N | PY | Y | Y | PY | Y | Y | Y | Y | N | Y | Y | Y | Y | Low |
| Wu, 2025 | 39928298 | Y | Y | Y | Y | Y | Y | N | PY | Y | Y | Y | N | Y | Y | Y | Y | Low |
| Xie, 2018 | 30083109 | Y | N | Y | Y | N | Y | PY | Y | N | Y | Y | N | Y | Y | Y | Y | Critically Low |
| Xuan, 2021 | 33269543 | Y | N | Y | Y | Y | Y | PY | Y | Y | Y | Y | Y | Y | Y | Y | Y | Low |
| Yao, 2014 | 24756759 | Y | N | Y | PY | Y | Y | PY | PY | Y | N | Y | N | N | Y | Y | N | Critically Low |
| Ye, 2016 | 28230025 | Y | N | Y | PY | Y | Y | N | PY | N | Y | Y | N | Y | N | Y | N | Critically Low |
| Zeng, 2013 | 24194957 | Y | N | Y | PY | Y | Y | PY | PY | N | Y | Y | N | Y | Y | Y | Y | Critically Low |
| Zeng, 2016 | 27294431 | Y | N | Y | Y | Y | Y | PY | PY | N | Y | Y | N | Y | Y | Y | Y | Critically Low |
| Zhang, 2020 | 31697412 | Y | N | N | Y | Y | Y | N | Y | Y | Y | Y | Y | Y | Y | Y | Y | Critically Low |
| Zhang, 2023 | 37689446 | Y | Y | N | Y | Y | Y | PY | PY | Y | Y | Y | N | Y | Y | Y | Y | Moderate |
| Chauca-Bajana, 2026 | 41744919 | Y | Y | Y | Y | Y | Y | PY | PY | Y | Y | Y | N | Y | Y | Y | Y | High |
| Duan, 2026 | 41889038 | Y | N | Y | Y | Y | N | PY | PY | Y | Y | Y | N | Y | Y | Y | Y | Low |
| Li, 2026 | 41800498 | Y | Y | Y | Y | Y | Y | PY | Y | Y | Y | Y | Y | Y | Y | Y | Y | High |
| Ridho, 2025 | - | Y | Y | Y | Y | Y | Y | PY | PY | Y | Y | Y | Y | Y | Y | Y | Y | High |
| Cueva, 2026 | 42074956 | Y | Y | Y | Y | Y | Y | PY | Y | Y | Y | Y | N | Y | Y | Y | Y | High |
| Manzaba, 2026 | 42074964 | Y | Y | Y | Y | Y | Y | PY | PY | Y | Y | Y | N | Y | N | Y | Y | Moderate |

**Abbreviation:** Y, yes; PY, partial yes; N, no.

**Key:**

Q1 – Did the research questions and inclusion criteria for the review include the components of PICO? (yes/no)

Q2 – Did the report of the review contain an explicit statement that the review methods were established prior to the conduct of the review and did the report justify any significant deviations from the protocol? (yes/partial yes/no)

Q3 – Did the review authors explain their selection of the study designs for inclusion in the review? (yes/no)

Q4 – Did the review authors use a comprehensive literature search strategy? (yes/partial yes/no)

Q5 – Did the review authors perform study selection in duplicate? (yes/no)

Q6 – Did the review authors perform data extraction in duplicate? (yes/no)

Q7 – Did the review authors provide a list of excluded studies and justify the exclusions? (yes/partial yes/no)

Q8 – Did the review authors describe the included studies in adequate detail? (yes/partial yes/no)

Q9 – Did the review authors use a satisfactory technique for assessing the risk of bias (RoB) in individual studies that were included in the review? (yes/partial yes/no)

Q10 – Did the review authors report on the sources of funding for the studies included in the review? (yes/no)

Q11 – If meta-analysis was performed, did the review authors use appropriate methods for statistical combination of results? (yes/no/no meta-analysis conducted)

Q12 – If meta-analysis was performed, did the review authors assess the potential impact of RoB in individual studies on the results of the meta-analysis or other evidence synthesis? (yes/no/no meta-analysis conducted)

Q13 – Did the review authors account for RoB in individual studies when interpreting/discussing the results of the review? (yes/no)

Q14 – Did the review authors provide a satisfactory explanation for, and discussion of, any heterogeneity observed in the results of the review? (yes/no)

Q15 – If they performed quantitative synthesis, did the review authors carry out an adequate investigation of publication bias (small study bias) and discuss its likely impact on the results of the review? (yes/no/no meta-analysis conducted)

Q16 – Did the review authors report any potential sources of conflict of interest, including any funding they received for conducting the review? (yes/no)

**SFig 1. Schematic overview of overlap management, author’s own meta-analysis, and primary evidence selection.**


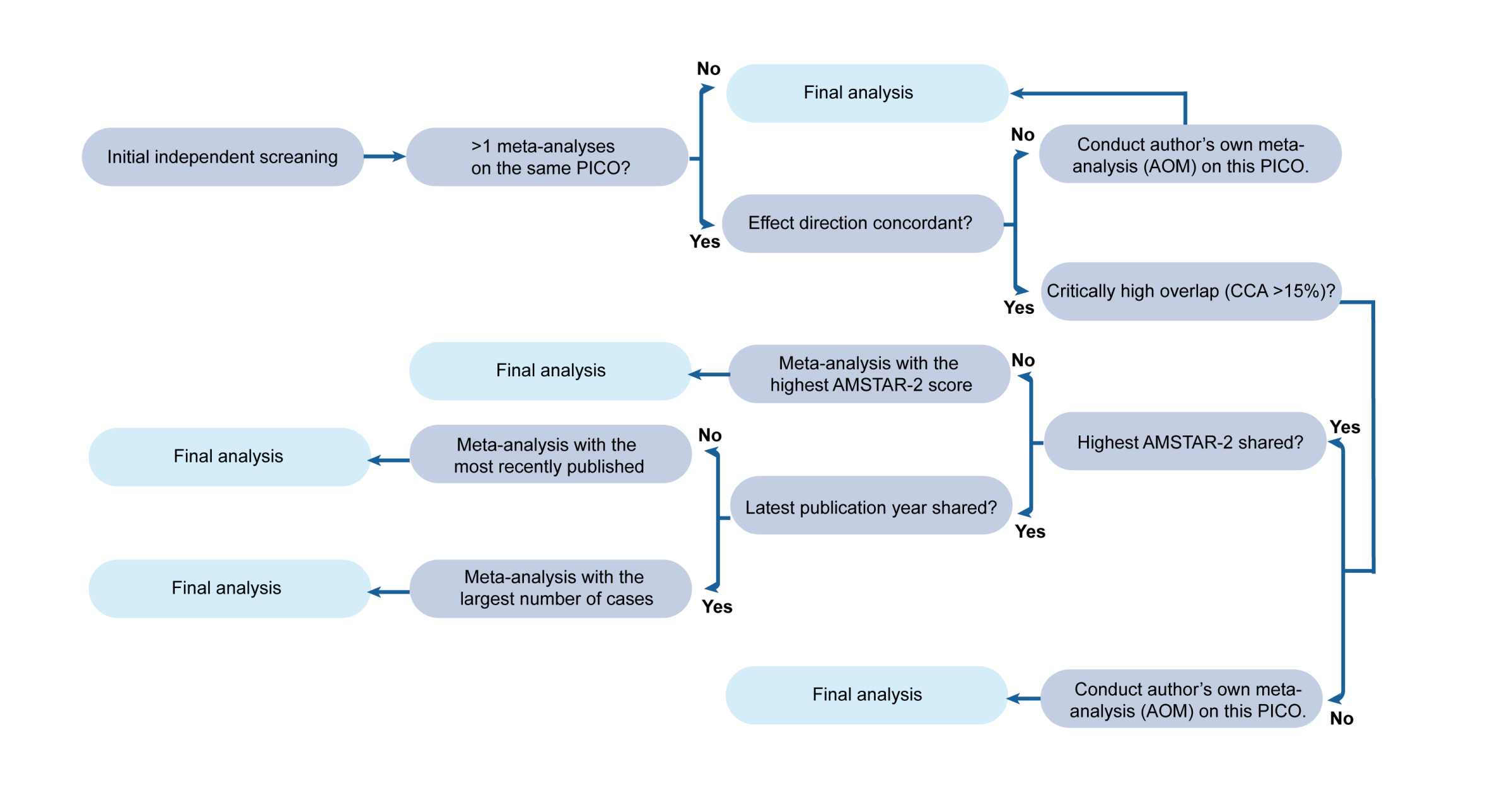


**SFig 2. Characteristics of main associations in periodontal diseases and site-specific cancers.**

**
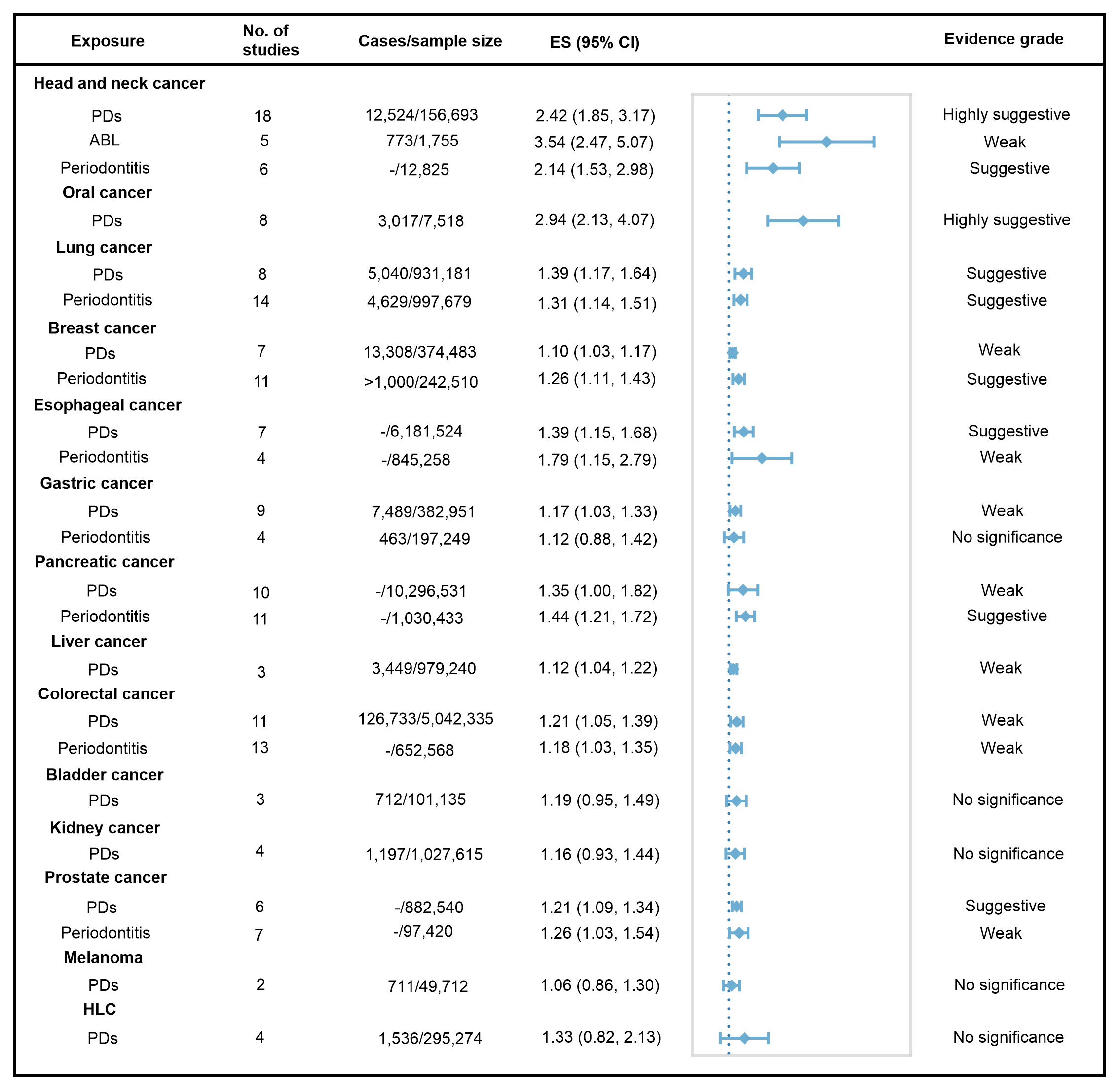
**

Abbreviation: ES, estimate size; PDs, periodontal diseases; ES, estimate size; CI, confidence interval; HLC, hematopoietic and lymphatic cancer.

**SFig 3. Quality assessment of 38 eligible studies using AMSTAR-2 tool.**


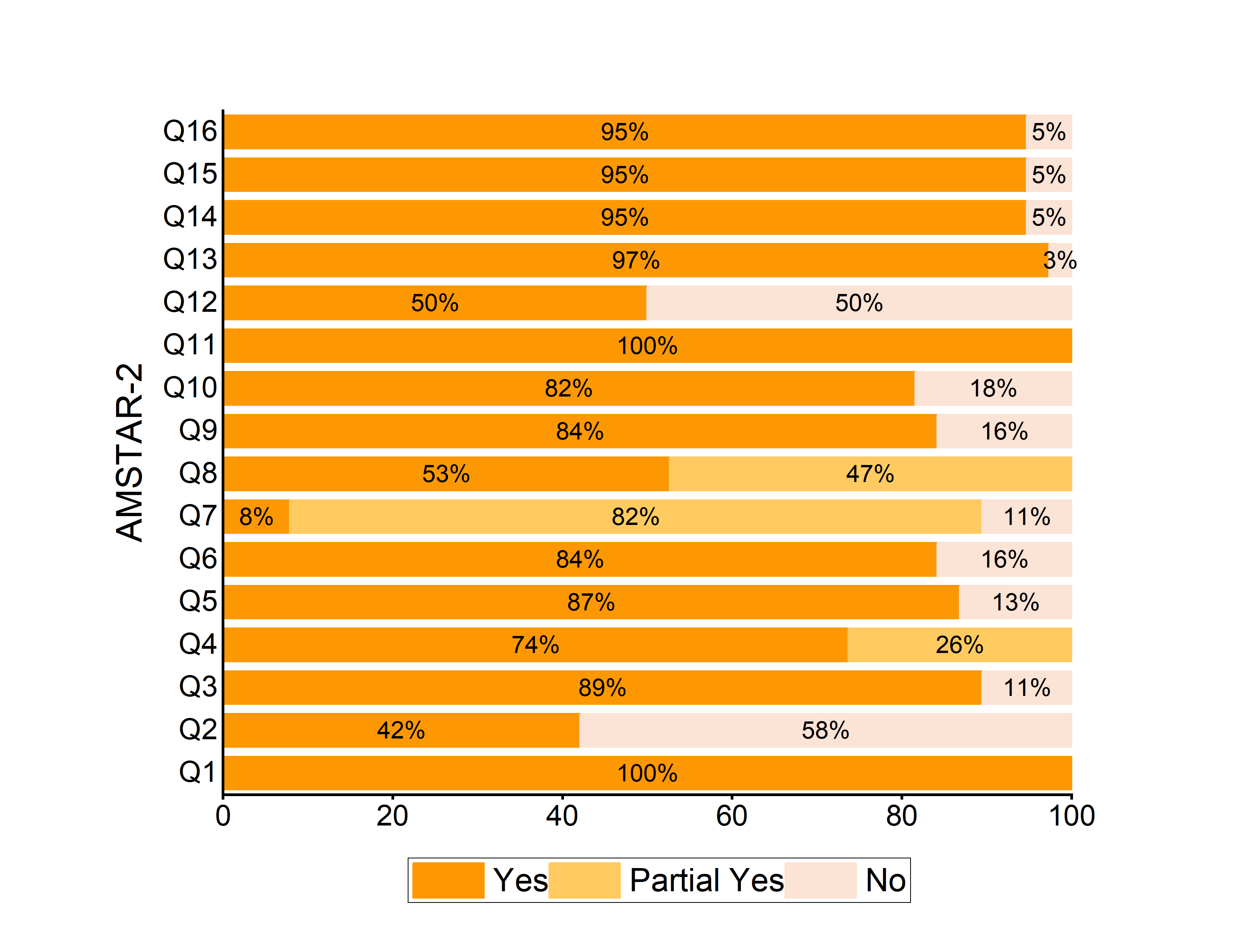

Supplement: Supplementary file 1 [file Table1.docx]
